# Cell-type-specific profiling of loaded miRNAs from *Caenorhabditis elegans* reveals spatial and temporal flexibility in Argonaute loading

**DOI:** 10.1038/s41467-021-22503-7

**Published:** 2021-04-13

**Authors:** Christopher A. Brosnan, Alexander J. Palmer, Steven Zuryn

**Affiliations:** 1 Clem Jones Centre for Ageing Dementia Research, Queensland Brain Institute, The University of Queensland, Brisbane, Australia; 2 Present Address: Queensland Alliance for Agriculture and Food Innovation, The University of Queensland, Brisbane, Australia

**Keywords:** Caenorhabditis elegans, miRNAs

## Abstract

Multicellularity has coincided with the evolution of microRNAs (miRNAs), small regulatory RNAs that are integrated into cellular differentiation and homeostatic gene-regulatory networks. However, the regulatory mechanisms underpinning miRNA activity have remained largely obscured because of the precise, and thus difficult to access, cellular contexts under which they operate. To resolve these, we have generated a genome-wide map of active miRNAs in *Caenorhabditis elegans* by revealing cell-type-specific patterns of miRNAs loaded into Argonaute (AGO) silencing complexes. Epitope-labelled AGO proteins were selectively expressed and immunoprecipitated from three distinct tissue types and associated miRNAs sequenced. In addition to providing information on biological function, we define adaptable miRNA:AGO interactions with single-cell-type and AGO-specific resolution. We demonstrate spatial and temporal dynamicism, flexibility of miRNA loading, and suggest miRNA regulatory mechanisms via AGO selectivity in different tissues and during ageing. Additionally, we resolve widespread changes in AGO-regulated gene expression by analysing translatomes specifically in neurons.

## Abstract (web summary)

*C. elegans* miRNAs associate with AGO proteins ALG-1 and ALG-2. Here the authors provide a map of miRNAs loaded into ALG-1 and ALG-2 from intestine, body wall muscles and nervous system in *C. elegans* providing insights into spatial and temporal AGO loading flexibility.

## Introduction

The development of complex organisms and their adaptation to the surrounding environment requires the implementation of precise gene expression networks. These networks must be tightly controlled and tuned both during time (temporally) and in a cell-type specific manner (spatially). While the core gene expression network is set by RNA polymerase II (Pol II)-driven transcription and dictated by complex transcription factor cohorts, the modulation and fine-tuning of these networks is often achieved post-transcriptionally.

MicroRNAs (miRNAs) are highly conserved modulators of post-transcriptional gene expression. These 21–24 nt class of small RNAs are encoded within longer non-coding RNAs, that form extended foldback structures known as pri-miRNAs and are transcribed by Pol II. The foldback structure is processed by Drosha and DGCR8 (Pasha in nematodes) into a “pre-miRNA”, which is subsequently exported to the cytoplasm^1,2^. Here, Dicer proteins process the pre-miRNA into a mature miRNA duplex^3–5^. Once processed, mature miRNAs are loaded into Argonaute (AGO) proteins, which together constitutes the core of the RNA-induced silencing complex (RISC), whereupon they bind with imperfect base-pair complementary to their target mRNA to elicit their regulatory role. This pairing typically occurs in the 3′ untranslated region of protein coding mRNAs with nucleotides 2–7 at the 5′ end (known as the seed region) of the single-stranded guide miRNA, allowing the direct repression of the target mRNA. This silencing effect is elicited as translational repression, which is often coupled with transcript decay or via direct endonucleolytic cleavage (slicing) catalyzed by AGO itself^6,7^. The sequence specificity of any RNA silencing reaction is conferred by the guide miRNA, but owing to the flexibility in targets granted by the six-nucleotide seed region, single miRNAs can potentially target hundreds of mRNAs^8^. However, molecular evidence of the bulk of these interactions is still largely lacking, with the discovery of miRNAs far outpacing their assignment to targets and cellular functions.

MiRNA-mediated post-transcriptional regulation generates a more complex topology of gene expression from that produced solely from nuclear events, enabling developmental complexity, flexibility, and robustness. MiRNAs are involved at all levels of development from the early stages of embryogenesis to the final distinguishing molecular events that precise terminal differentiation^9–11^. This is exemplified most strikingly in *C. elegans*, where a single miRNA has been found to direct the terminal fate decisions of otherwise identical pairs of neurons, segregating and defining each neuron with a distinct identity, physiology, and function^9,12^. Aside from specifying multicellularity, miRNAs play an essential role in maintaining cellular homeostasis and can act to rapidly, and often reversibly, adjust regulatory networks in response to or in spite of environmental fluctuations^13–15^.

In addition to miRNA expression, processing, and stability, their interaction with effector AGO proteins is the most important step in defining their activity and thus their ultimate functions. There are 25 different AGOs in *C. elegans*, but only three appear to be dedicated exclusively to miRNA pathways. The first two, Argonaute-Like Gene 1 (ALG-1) and ALG-2 are widely expressed and share high sequence similarity (81% at the amino acid level)^16,17^. On the contrary, ALG-5 expression is restricted to germ cells, associating with a subset of germline-enriched miRNAs and being required for normal reproductive development^18^. In many species, similar scenarios exist whereby multiple AGOs are available for miRNA loading within the same somatic cells. However, it remains unclear whether highly individualized miRNA:AGO silencing complexes form within certain cell types, and whether this customizes miRNA function within a particular cellular context. Although genome-wide views of temporal miRNA expression have been a core facet of metazoan research, genome-wide spatial profiling involving fluorescence activated cell sorting (FACS), microdissection, immunoprecipitation, or novel enzymatic techniques^19–23^ fall short of integrating the activities of miRNAs at cell-type resolution. This information would not only aid in our ability to assign biological functions to miRNAs, but also reveal relationships between AGO proteins and miRNAs that may represent new layers of miRNA regulation.

Here, we focussed on miRNAs loaded into silencing complexes by immunoprecipitating the two main somatic AGOs known to bind miRNAs in *C. elegans*, ALG-1, and ALG-2. By expressing tagged versions of these AGOs under cell-type-specific promoters, we generated a genome-wide map of loaded miRNAs across three major tissue types. We identified a large portion of miRNAs with strong associations to either ALG-1 or ALG-2 in the intestine, body wall muscles, or nervous system. Most miRNAs exhibited a highly cell-type-specific loading pattern, with many individual miRNAs also demonstrating AGO-specific preferences within particular cell types. Due to the sensitivity of the technique, we discovered not only multiple novel miRNAs, but also a rich array of miRNA isoforms that exhibited cell- and AGO-specific loading patterns. Finally, we demonstrated at the molecular level that AGOs act with both spatial and temporal loading specificity, and that ALG-2, which we found to modulate widespread translatome changes in the nervous system, can act in a surrogate capacity when the function of ALG-1 is reduced or compromised both genetically or physiologically during ageing.

## Results

### Establishing a system to spatially profile loaded miRNAs

**Fig. 1:**
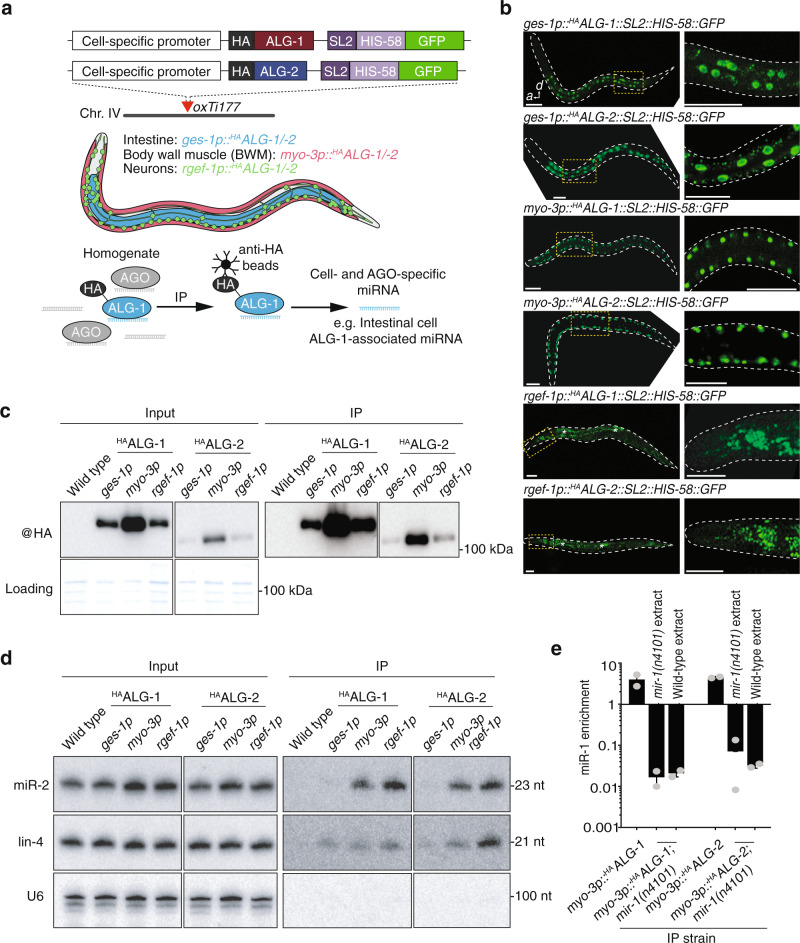
Cell-type-specific profiling of AGO-loaded miRNAs. **a** Overview of cell-type-specific miRNA profiling technique. HA-tagged copies of ALG-1 or ALG-2 were driven by cell-type-specific promoters, allowing immunoprecipitation of AGO-loaded miRNAs from individual tissue types from total worm homogenates. **b** Whole animal fluorescence images of cell-type-specific ^HA^ALG-1 and ^HA^ALG-2 lines demonstrating specific expression from intestine, body wall muscles, or neurons. Scale bars, 50 µm. Asterisks indicate intestine autofluorescence. Images are representative of >50 independent animals. **c** Western blot analysis of input (left) and immunoprecipitated (right) AGO complexes. All images are from the same blot and are equally exposed. Coomassie blue serves as a loading control. **d** RNA gel blot analysis of input (left) and AGO-immunoprecipitated miRNAs (right). U6 serves as a loading control. **e** Quantitative RT-PCR on immunoprecipitated AGO:miRNA complexes showing either enrichment >1 or lack of enrichment <1 of miR-1 immunoprecipitated with ^HA^ALG1 or ^HA^ALG2 in the genetic backgrounds and conditions indicated. Error bars represent +/− s.e.m. of three biological replicates. *P* values were calculated using one-way ANOVA with Dunnett’s multiple comparisons test. All immunoprecipitation experiments were repeated three times with similar results. HA hemagglutinin, SL2 splice leader sequence, GFP green fluorescent protein, IP immunoprecipitation.

To gain insight into the spatial function of miRNAs at a genome-wide level, we generated a cell-type specific-map of miRNAs bound to their effector AGO proteins. To achieve this goal, we constructed a series of transgenic *C. elegans* strains in which either ALG-1 or ALG-2 was labeled with an N-terminal hemagglutinin (HA) epitope tag and expressed exclusively in select cell types (Fig. 1a). The *HA::alg-1* and *HA::alg-2* transgenes were placed under the control of promoters driving expression in three major somatic tissue types, the intestine (*ges-1p*), the body wall muscle (BWM, *myo-3p*), and the nervous system (*rgef-1p*) (Fig. 1a). *Mos1*-mediated single-copy insertion (mosSCI)^24^ was used to target each transgene to a specific integration site on chromosome IV, thereby providing stable and comparable expression. We confirmed correct cell-type-specific expression patterns in live animals by visualizing green fluorescent protein (GFP) signals within nuclei using an *SL2*::*his-58::gfp* cassette (splice leader 2, histone tagged with GFP) incorporated into each *HA::alg-1/2* construct (Fig. 1b). Immunoblotting revealed that each of the ^HA^ALG-1 and ^HA^ALG-2 tissue-specific fusion proteins was expressed and migrated at the expected size, and that it could be successfully purified from populations of whole animals using immunoprecipitation (Fig. 1c). Differences in ALG protein levels between cell types largely reflected the strength of the tissue-specific promoters used to control their expression. Intratissue differences between ^HA^ALG-1 and ^HA^ALG-2 protein levels were most likely attributable to variations in protein stability between the two AGOs, given that the expression criteria (5′ and 3′ *cis*-regulatory sequences, transgene copy number and insertion loci) are identical between them. Indeed, AGO stability positively correlates with miRNA loading^25^, suggesting that a greater proportion of cellular miRNAs associated with ALG-1, a notion supported by the sequencing results below. Although this or other forms of protein turnover may be occurring, neither a priori should affect the ability to normalize our data and thereby accurately profile loaded miRNAs.

Next, we investigated whether immunoprecipitation of ^HA^ALG-1 and ^HA^ALG-2 from homogenized populations of each of the transgenic strains could reveal stably associated miRNAs in a cell-specific and AGO-specific manner. To this end, we first examined their association with two canonical miRNAs, miR-2 and lin-4, by RNA gel blotting (Fig. 1d). In input samples, we noted no obvious change in abundance of either of these miRNAs between wild-type and transgenic populations, suggesting that the AGO transgenes did not affect native miRNA levels. When compared to immunoprecipitations performed on wild-type animals lacking any HA-labeled AGOs, we detected an enrichment of both miRNAs in a range of context-dependent associations with ALG-1 and ALG-2. Specifically, we found that miR-2 stably associated equally with both ALG-1 and ALG-2 but was predominantly enriched with AGOs purified from the BWM and intestine (Fig. 1d). lin-4 was more evenly enriched across tissue types but showed preferential binding for ALG-2 in neurons (Fig. 1d). These results suggest first, that the epitope-labeled AGOs were functional in their ability to bind mature miRNAs, and second, that we were able to resolve cell-type and AGO-type differences in these associations that hinted at the complexity of miRNA activity.

The primary principle underlying our approach is its ability to purify miRNAs from specific cell or tissue types for direct comparison. During homogenization, it is conceivable that miRNAs released from one tissue type could non-specifically interact with AGOs from another tissue type and undermine the analysis of cell-type-specific miRNA:AGO complexes. To confirm that only genuine, in vivo assembled miRNA:AGO complexes were immunoprecipitated, we crossed the muscle-specific ^HA^ALG-1 and ^HA^ALG-2 lines (*myo-3p::*^*HA*^*ALG-1* and *myo-3p::* ^*HA*^*ALG-2*) to a genetic background lacking the miR-1 miRNA [*mir-1(n4101)*]. miR-1 regulates retrograde signaling at neuromuscular junctions and is expressed exclusively in several muscle lineages, including BWM^26^. Consistent with this notion, we found that miR-1 was strongly enriched in immunoprecipitates of both ^HA^ALG-1 and ^HA^ALG-2 expressed in BWM in wild-type animals, but was undetectable in *mir-1(n4101)* backgrounds (Fig. 1e). Demonstrating that miR-1:AGO complexes did not assemble post-homogenization and that our approach represents an accurate in vivo capture of the cellular context of miRNA:AGO interaction, we found that supplementation of exogenous miR-1 into *mir-1(n4101);*^*HA*^*alg-1/-2* lysates did not result in the immunoprecipitation of miR-1::^HA^ALG-1 or miR-1::^HA^ALG-2 complexes (Fig. 1e and Supplementary Fig. 1).

### A map of cell-type specific, loaded *C. elegans* miRNAs

**Fig. 2:**
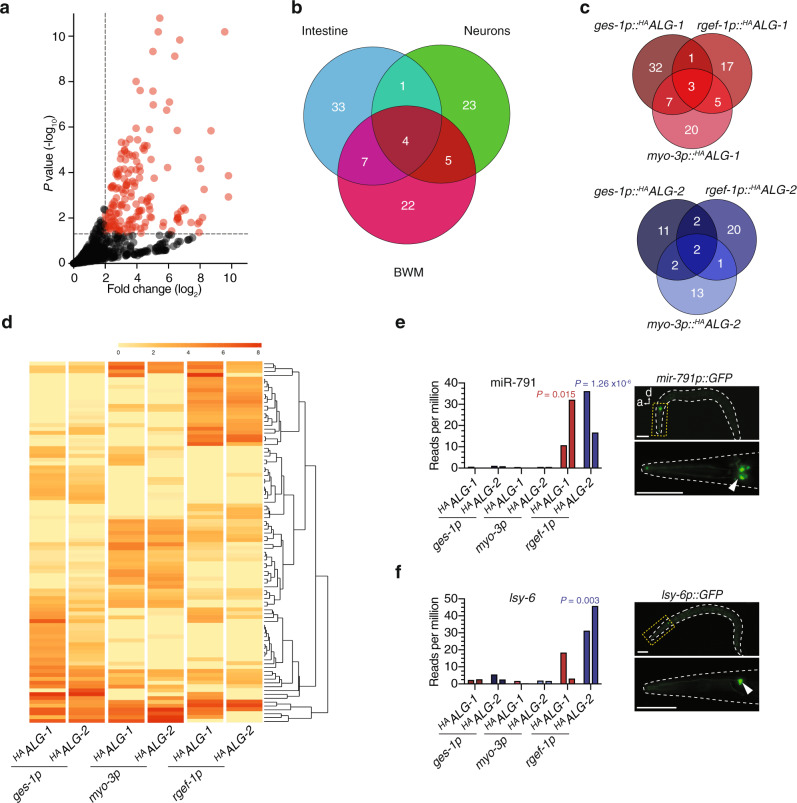
Genome-wide profiling of cell-type- and AGO-specific miRNAs. **a** Volcano plot showing all reference miRNAs enriched (log_2_ fold change > 2, *P* < 0.05 relative to wild type) in immunoprecipitations of ^HA^ALG-1 or ^HA^ALG-2 in one or more cell-type (red). *P* value was calculated by fitting a two-tailed negative binomial model to processed read counts, according to edgeR pipeline for pairwise comparisons between multiple groups. **b** Venn diagram representation of all AGO-loaded (either ALG-1 or ALG-2) cell-type-specific miRNAs in intestine (blue), neurons (green) or body wall muscles (BWM, pink). **c** Venn-diagram depicting cell-type-specific and AGO-type-specific loading of miRNAs (ALG-1—top and ALG-2—bottom). **d** Heatmap showing unsupervised clustering of ALG-1 or ALG-2 cell-type-specific loading of miRNAs. **e** Sensitivity of cell-type-specific immunoprecipitation demonstrated by detection of miR-791 expressed from three neurons (right—fluorescence image of *miR-791p::GFP*). Scale bars, 50 µm. **f** Enhanced sensitivity of cell-type-specific immunoprecipitation demonstrated by detection of *lsy-6* expressed from a single neuron (right—fluorescence image of *lsy-6p::GFP*). **e**, **f** Graph shows reads per million in indicated cell types of two biological replicate experiments, two bars. Scale bars, 50 µm.

Having validated our ability to accurately profile cell-type-specific miRNA:AGO interactions, we next performed small RNA sequencing of the immunoprecipitated samples to generate a genome-wide view of loaded miRNAs. Deep-sequencing yielded at least 10 million reads per library (two biological replicates for each sample), including that of non-transgenic wild-type samples that had undergone an identical immunoprecipitation protocol. The wild-type non-transgenic populations were analysed in order to provide a baseline level of background miRNAs, that were non-specifically enriched during immunoprecipitation of AGO complexes, to which all other samples could be compared. Overall, we detected 95 miRNAs (over one third of the total known *C. elegans* miRNAs) that were significantly associated (log_2_ fold change > 2, *P* < 0.05) with either ALG-1 or ALG-2 in the intestine, BWM, or nervous system (Fig. 2a and Supplementary Data 1). Indicatory of a high level of cell-type-specific miRNA function, the vast majority of miRNAs exclusively loaded into AGOs in a single cell-type (Fig. 2b). Intestinal cells contained the highest number of these miRNAs (33), with neurons and muscle cells containing near identical numbers (23 and 22, respectively). The sharing of miRNAs between two tissues was a more common feature between intestine and muscle (7), and neurons and muscles (5), with only one miRNA in common between the intestine and nervous system (Fig. 2b). Overall we found that more miRNAs loaded into ALG-1 than ALG-2, although both ALGs displayed high levels of cell-type-specific miRNA loading, albeit in different proportions (Fig. 2c, d).

To assess the sensitivity of our approach, we chose to focus on neuron-specific miRNAs, which are often expressed in only a few cells within the total population of 300 neurons in each animal^27^. miR-791 is expressed exclusively in three pairs of carbon dioxide sensory neurons (BAG, AFD, and ASE; Fig. 2e), acting to specifically silence target mRNAs that would otherwise disrupt normal neuronal physiology^28^. We were readily able to detect a strong association of miR-791 with both ALG-1 and ALG-2 in neurons but not in any other tissue type (Fig. 2e), demonstrating the ability of our approach to accurately uncover miRNA:AGO interactions occurring in just six cells of the whole animal. Impressively, the *lsy-6* miRNA, which during development is restricted to only a single neuron (ASEL) in the whole animal (Fig. 2f), where it directs left-right neuronal asymmetry^12^, was also efficiently detected in neurons where it associated more strongly with ALG-2 than ALG-1 (Fig. 2f). Three other neuronal miRNAs (miR-790, miR-793, and miR-1821), which also display highly specialized cell-type-specific expression patterns within the ASE sensory neuron pair^19,28^, were also strongly detected in association with neuronal AGOs (Supplementary Fig. 2). Together, these results suggest that our technique is both sensitive and accurate at single-cell resolution in whole animals.

### miRNAs display preferential, flexible, and temporally dynamic AGO loading

**Fig. 3:**
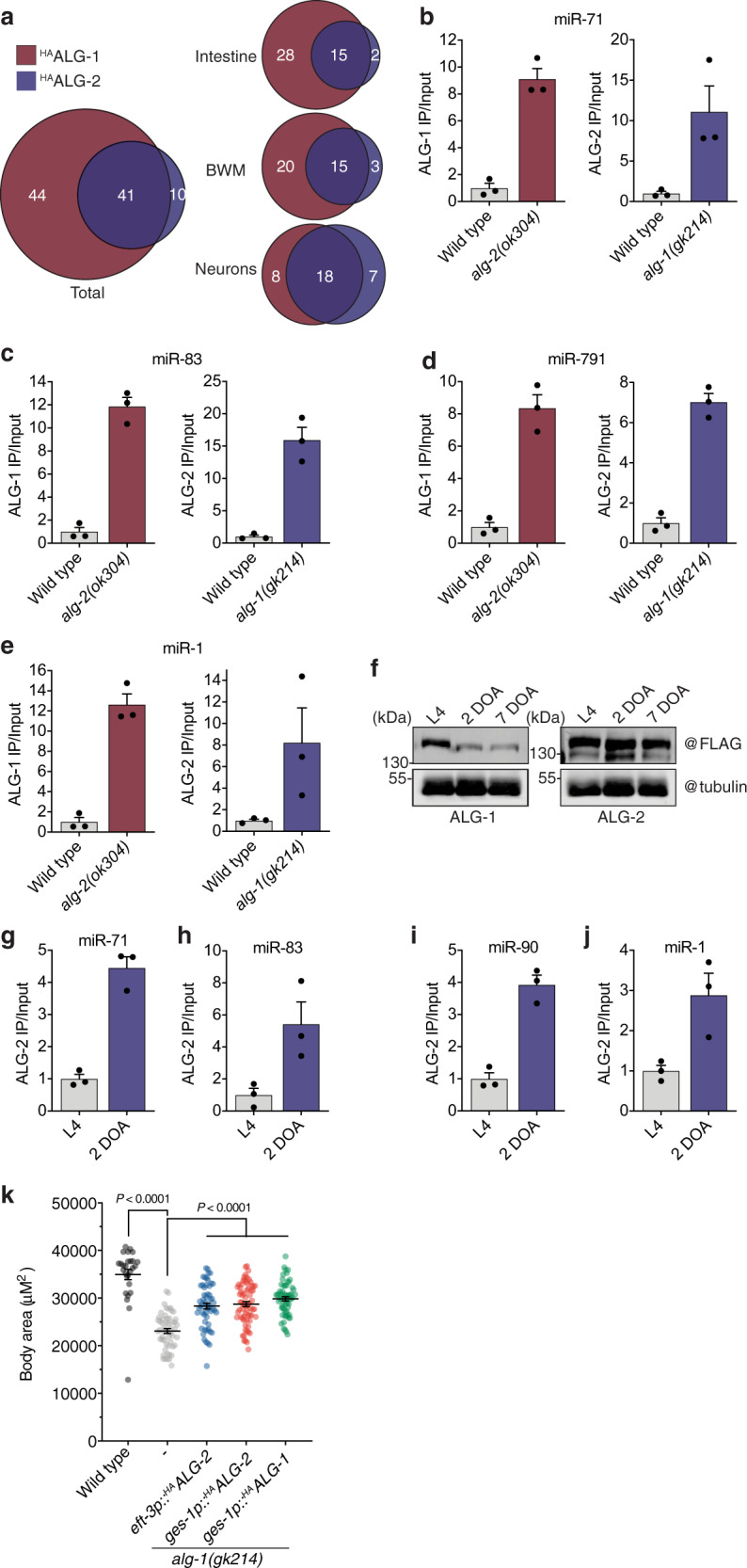
Preferential, flexible and temporally dynamic AGO loading. **a** Proportion of miRNAs loaded into ^HA^ALG-1 or ^HA^ALG-2 in combined (left—total) or separate tissue types (right). **b** Quantitative RT-PCR of miR-71 loading into 3×FLAG::GFP::ALG-1 (left) or 3×FLAG::RFP::ALG-2 (right) in the indicated genetic backgrounds. Each dataset represents the ratio of IP normalized to input. **c**–**e** The same as shown in **b** for the intestine-specific miR-83 (**c**), neuronal-specific mir-791 (**d**), and muscle-specific miR-1 (**e**). **f** Western blot analysis of 3×FLAG::GFP::ALG-1 (left) or 3×FLAG::RFP::ALG-2 (right) at L4, 2-day-old adult (2 DOA) and 7-day-old adult (7 DOA). Tubulin represents loading control. Western blots for ALG levels during aging were repeated at least three times with similar results. **g** Quantitative RT-PCR of miR-71 loading into 3×FLAG::RFP::ALG-2 at the developmental stage indicated. Each dataset represents IP values normalized to input. **h**–**j** The same as shown in **g** for intestine-specific miR-83 (**h**), neuronal-specific mir-90 (**i**), and muscle-specific miR-1 (**j**). Error bars for every column graph represent +/− s.e.m. of three biological replicate immunoprecipitations. **k** Body size quantification of the indicated genotypes after recovery from starvation. Error bars represent +/− s.e.m. *P* values calculated using one-way ANOVA with Tukey’s multiple comparisons test. *n* ≥ 27 biologically independent animals for each strain tested.

When we focussed on AGO associations amongst the total pool of enriched miRNAs, a strong preference was observed for miRNA:ALG-1 specific interactions (44) over miRNA:ALG-2 specific interactions (10), with 41 miRNAs displaying overlapping association with both AGOs (Fig. 3a). Within individual tissues, this trend held true for the intestine and BWM, but varied dramatically in the nervous system. In neurons, individual miRNAs were evenly distributed in their propensity to load exclusively into either ALG-1 or ALG-2 (Fig. 3a), suggesting that ALG-2 plays a more significant role in miRNA-mediated repression in the nervous system than in either of the other major tissues studied here. Although these results demonstrated a preference for AGO-type-specific miRNA interactions within individual cell types, given the high homology, lack of nucleotide preference for miRNA loading, and shared subcellular localization of ALG-1 and ALG-2^16,17,29^, we questioned whether miRNAs could re-load between AGO proteins to provide regulatory flexibility under cellular situations in which AGO availability was altered. Using CRISPR-Cas9, we engineered endogenous ALG-1 with an N-terminal 3xFLAG::GFP tag in an *alg-2(ok304)* null background. miR-71, which is associated with both ALG-1 and ALG-2 in most major tissue types, was 8-fold more enriched with ALG-1 in *alg-2(ok304)* mutants than in wild-type animals (Fig. 3b), suggesting a surrogate role for ALG-1, which increased in abundance in the absence of *alg-2* (Supplementary Fig. 3). To determine whether ALG-1 could compensate for ALG-2 in individual cell-types, we focussed on those miRNAs whose loading was restricted to a specific tissue. The intestine-enriched miR-83, neuron-specific miR-791, and muscle-specific miR-1 all displayed greater association with ALG-1 in *alg-2(ok304)* backgrounds (Fig. 3c–e), suggesting that the surrogate role of ALG-1 was not tissue-dependent. Indeed, this general trend held true for other tissue-specific miRNAs (Supplementary Fig. 4). In the reciprocal experiment, where we used an endogenously labeled 3xFLAG::RFP::ALG-2 strain^16^ harboring a deletion in *alg-1(gk214)*, we observed a converse effect whereby ALG-2 compensated for the lack of ALG-1 by associating more strongly with multiple miRNAs (Fig. 3b–e and Supplementary Fig. 4), even though the abundance of ALG-2 was not increased by a loss of *alg-1* (Supplementary Fig. 3).

Under certain physiological contexts, AGOs may change in cellular abundance, resulting in potential switches in miRNA pathway regulation. Consistent with previous findings^16^, we found that ALG-1 protein levels decreased during the onset of adulthood and continued to decline during ageing, with ALG-2 levels remaining relatively stable over time (Fig. 3f). Although the molecular determinants of this downregulation are unknown, probing miRNA:AGO interactions over time revealed that individual miRNAs became more associated with ALG-2 as animals aged (Fig. 3g–j and Supplementary Fig. 5). This included miRNAs with widespread loading patterns, such as miR-71 and miR-35 (Fig. 3g and Supplementary Fig. 5), as well as intestine- (miR-83, miR-63, miR-243), neuron (miR-90, miR-60) and muscle-specific (miR-1) miRNAs (Fig. 3h–j and Supplementary Fig. 5). Whether this temporally regulated interchangeability between AGO types serves a biological function during ageing remains to be determined. However, *alg-1* has been shown to promote longevity whereas *alg-2* restricts longevity in an insulin/IGF-1 signaling-dependent manner^16^, suggesting that their functions can be antagonistic later in life.

Taken together, these results suggest that individual miRNAs preferentially associate with specific AGOs under particular cellular contexts. However, the system maintains flexibility when the AGO abundance or cellular context changes, indicating their capacity to act as mutual surrogates for each other’s activities. In support of this idea, we found that *alg-2* could functionally compensate for the removal of *alg-1* under certain contexts. For example, developmental rates under multiple growth conditions (fed and temporarily starved) were reduced in *alg-1(gk214)* mutants when compared to their wild-type counterparts (Fig. 3k and Supplementary Fig. 6). This defect could be partially rescued by the overexpression of *alg-2* under the ubiquitous *eft-3* promoter (Fig. 3k). Indeed, selective overexpression of *alg-2* in the intestine (*ges-1p::alg-*2) had the equivalent rescuing ability to overexpression of *alg-1* in the intestine (*ges-1p::alg-1*), demonstrating that the AGO proteins were functionally interchangeable in this tissue during development (Fig. 3k).

### Comparison of miRNA expression, abundance, and AGO loading

**Fig. 4:**
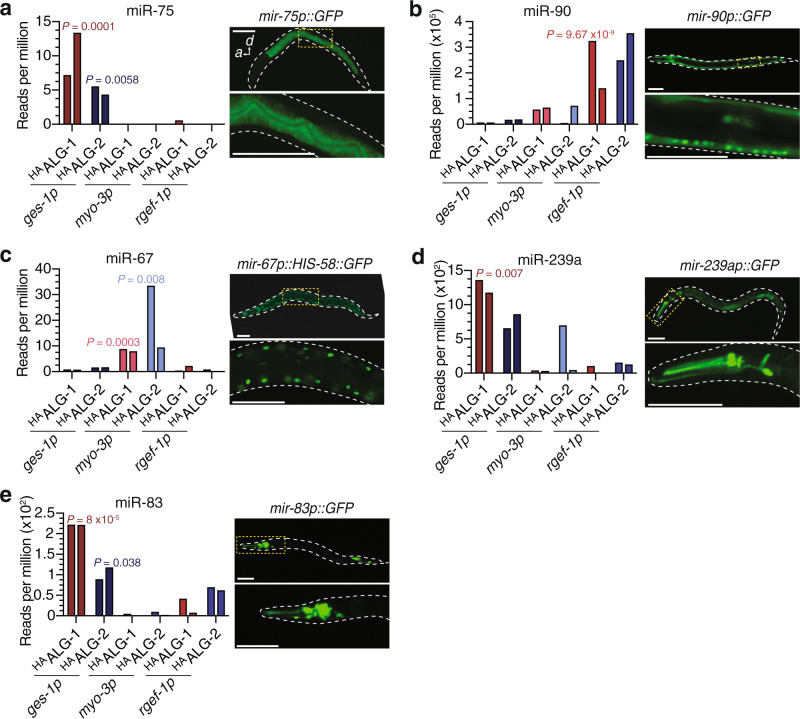
Comparison of expression and cell-type-specific loading of miRNAs. **a** Read counts per million in individual AGO- and cell-type-specific libraries for miR-75 showing intestine enrichment. Live animal image (right) of the promoter of miR-75 fused to GFP, showing tight correlation of expression with loading profile. **b** Cell-type-specific read counts and promoter GFP fusion for neuron-enriched miR-90. **c** Cell-type-specific read counts and promoter GFP fusion for muscle-specific miR-67. **d** Intestine-specific loading of miR-239a shown by read counts in individual cell-type libraries. Promoter expression of miR-239a showing enrichment of fluorescence in neurons. **e** Intestine-enriched loading of miR-83 as compared to neuron-enriched expression. Graphs show reads per million in indicated cell types of two biological replicate experiments, two bars. Scale bars, 50 µm.

MiRNA loci fall under the same Pol II transcriptional control as protein coding genes, but how miRNA activity is regulated post-transcriptionally, particularly during the assembly of effector miRNA:protein complexes, remains relatively unexplored, especially from a spatial perspective. We compared patterns of expression, abundance, and assembly with AGOs to resolve potential points of post-transcriptional regulation of individual miRNAs in single cell-types. By analysing transcriptional reporters of miRNA promoters (with the assumption that they faithfully recapitulate endogenous expression), as well as specific examples of mature miRNA abundance obtained through Hen1 cell-type-specific profiling^19^, we found spatial correlations between all three levels of miRNA regulation, as observed for the sensory neurons described above (Fig. 2e, f, and Supplementary Data 2). For example, miR-75 was associated with both ALG-1 and ALG-2 exclusively in the intestine, which mirrored the expression pattern of a *mir-75p::GFP* transgene (Fig. 4a) as well as the reported enrichment of mature miR-75^19^. Indeed, multiple examples of intestinal miRNAs (miR-77, miR-238 and miR-243) matched this profile (Supplementary Fig. 7). The same was also true for the largely neuron-specific miR-90 (Fig. 4b) and the muscle-specific miR-67 (Fig. 4c).

Despite a frequently tight correlation, we also identified specific examples whereby the expression pattern of a miRNA and/or its cellular abundance diverged spatially from its association with AGOs and therefore potential activity. The vast majority of miR-239a, for instance, was found to be loaded into intestinal ALG-1 (Fig. 4d), despite a *mir-239ap::GFP* expression pattern indicating predominantly neuronal expression in the head (Fig. 4d) and mature miR-239a enrichment in neurons, intestine, and muscle^19,30^. Likewise, miR-83 was significantly enriched within the intestine in both ALG-1 and ALG-2, but was expressed in neurons and the intestine (Fig. 4e), with mature miR-83 being reported in intestine, neurons, and body wall muscle^19^. Although examples of post-transcriptional pathways that influence pri-miRNA or pre-miRNA processing, or stability between different tissue types and/or developmental states have been identified^31,32^, our results suggest that the formation of miRNA:AGO effector complexes can in some cases be uncoupled from the abundance of mature miRNAs within a cell, and may therefore represent a previously unappreciated control point for the regulation and segregation of miRNA activities between distinct cellular lineages.

### Detection of miRNA isoforms (isomiRs) and their differential AGO loading profiles to reference miRNAs

**Fig. 5:**
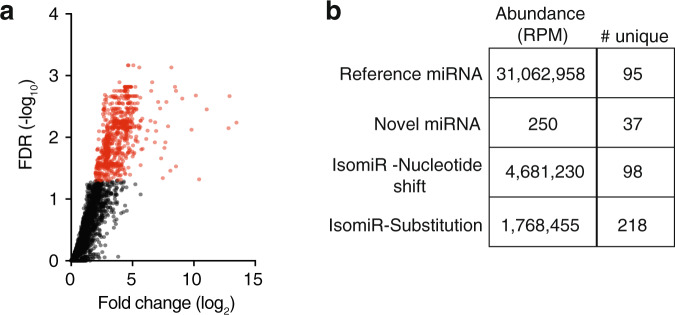
Discovery and detection of non-reference miRNAs. **a** Volcano plot showing all non-reference miRNAs enriched (log_2_ fold change > 2, FDR < 0.05 relative to wild type). **b** Table showing abundance (reads per million, RPM) and number of unique sequences (# unique) which make up each class of reference miRNA, novel candidate miRNA or isomiR (nucleotide shift or substitution).

Within our dataset of total miRNAs, ~17% of reads were distinguishable from miRNA with reference sequences (Fig. 5a, b and Supplementary Data 3). These often consisted of single nucleotide substitutions or nucleotide shifts at either the 3′ or 5′ end of the sequence. Such miRNA isoforms, termed isomiRs, can be derived as a result of RNA editing^33,34^, the activities of terminal-nucleotide transferases and 3′-exonucleases^35–37^, or imprecise or multiple cleavage events mediated by Drosha or Dicer during miRNA biogenesis. These “isomiRs” are often discarded as misprocessed artifacts and as such their biological roles remain unclear. Indeed, it is yet to be determined whether their function deviates from that of the reference miRNAs usually produced by the loci, even though the sequence alterations may influence target recognition and miRNA processing and stability. The highly significant read counts obtained in our AGO-immunoprecipitation samples suggested that the isomiRs revealed here were genuine and biologically relevant (Fig. 5a, b). Consistent with this concept, we found that many isomiRs displayed cell-type and AGO-type specificity that indicated function (Supplementary Fig. 8). In most instances, these patterns correlated with those of the reference miRNAs, supporting the notion that isomiRs act cooperatively with these miRNAs to target common biological pathways^38^. However, we also identified isomiRs (e.g., those derived from miR-71) with spatially divergent loading patterns, suggesting that they had distinct biological functions to their reference miRNA counterparts (Supplementary Fig. 9). Moreover, a fraction of isomiRs harbored nucleotide changes within their seed sequence, which could have a profound effect on target recognition and therefore biological function (Supplementary Fig. 9 and Supplementary Data 4). Together, these results suggest that isomiRs could greatly diversify the functionality of individual miRNAs through alterations in cellular loading patterns and seed sequences. Furthermore, the sensitivity of detection combined with the cell-type-specific resolution of our approach also enabled the discovery of multiple new candidate miRNA loci (Supplmentary Note 1).

### Cell-type-specific miRNA loading can help predict biological function

The identification of new miRNAs in virtually every species has far outpaced their assignment to biological roles, creating a void between discovery and function. In *C. elegans*, knockouts of individual miRNAs often have no obvious phenotype^39^, and it is only in sensitized (e.g., *alg-1* mutant) backgrounds that some miRNA-dependent phenotypes are revealed^40^. Having developed a cell-type-specific map of miRNA:AGO interactions, we predicted that individual miRNA functions could be derived by focussing on the cells in which they were loaded. In support of this idea, miR-1, which was expressed and loaded into AGOs in muscle cells (Supplementary Fig. 10a), has been shown to play a key role in retrograde signaling at neuromuscular junctions^26^. Likewise, miR-234, which was expressed and AGO-loaded exclusively in the nervous system (Supplementary Fig. 10b), was recently demonstrated to regulate genes involved in neuropeptide release^41^.

**Fig. 6:**
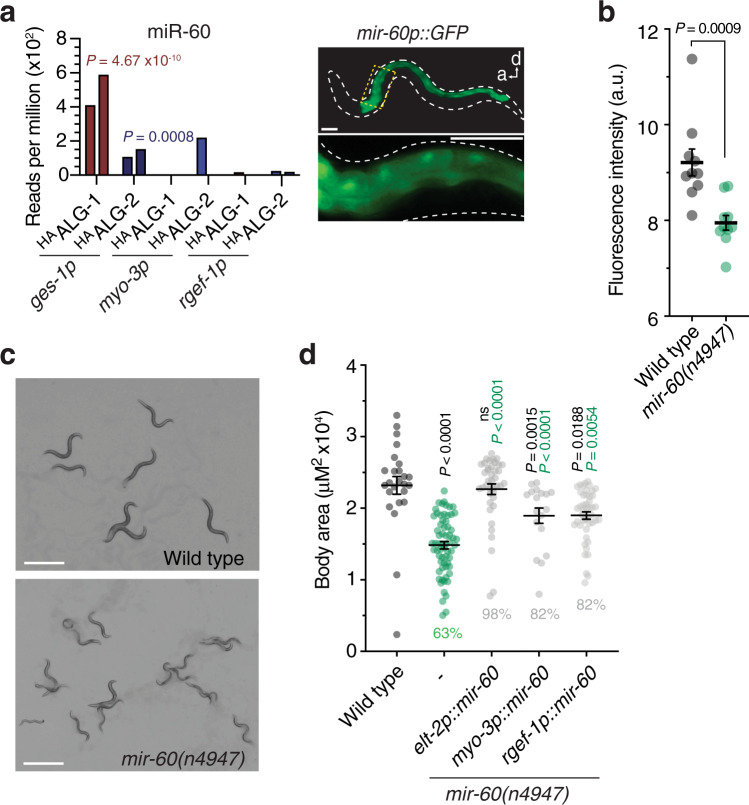
Functionality of intestine-specific miRNA. **a** Read counts showing intestine-specific loading of miR-60 and promoter GFP fusion showing intestine-specific expression. Graph shows reads per million in indicated cell types of two biological replicate experiments, two bars. Scale bars, 50 µm. **b** Quantification (fluorescence intensity) of fat content using Oil Red O staining on wild-type and *mir-60(n4947)* starvation-recovered worms. Error bars represent +/− s.e.m of ten independent animals. *P* value represents a two-way unpaired Students *t*-test. a.u., arbitrary units. **c** Wild type or *mir-60(n4947)* animals after recovery from starvation. Scale bars, 1 mm. **d** Quantification of body area of cell-type-specific complemented *mir-60(n4947)*. Percentages shown are the relative average body areas of animals compared to wild type. Error bars represent +/− s.e.m. *P* values represent one-way ANOVA with Tukey’s multiple comparisons test compared to wild type (black values) or *mir-60(n4947)* animals (green values). *n* ≥ 18 biologically independent animals for each strain tested.

The miRNAs miR-75 and miR-60 were associated with ALG-1 and ALG-2 exclusively in the intestine (Figs. 4a and 6a). To test whether these miRNAs were involved in intestinal-related functions, we studied them in the context of starvation, given that the primary function of the intestine is to absorb and process food-derived nutrients. Interestingly, we found that body fat content was significantly reduced in *mir-60(n947)* and *mir-75(n4472)* mutants (Fig. 6b and Supplementary Fig. 11a). Moreover, a temporary starvation period incurred during early development (Supplementary Fig. 6a) exacerbated developmetal rate defects in *mir-60(n947)* mutants and revealed that miR-60, but not miR-75, was required for full recovery to normal body size in adulthood (Fig. 6c, d and Supplementary Fig. 6b and 11b). This deficiency in the *mir-60(n947)* null mutant could be fully rescued, when we selectively expressed *mir-60* in the intestine (*elt-2p::mir-60*) as a single copy insertion (Fig. 6d), suggesting that miR-60 operates in the gut to promote recovery following periods of low food availability in early life, possibly through fat storage regulation. Moreover, these results suggest that the miRNA loading map generated here provides functionally relevant activity-based associations of miRNAs with AGOs. Interestingly, we also observed recovery from starvation, albeit partially, when we expressed *mir-60* exclusively in either the BWM (*myo-3p::mir-60*) or nervous system (*rgef-1p::mir-60*) of *mir-60(n947)* animals (Fig. 6d). Because a *mir-60p::gfp* reporter is expressed only in the intestine (Fig. 6a; Martinez et al.^42^), the same tissue type in which miR-60 exclusively interacted with ALG-1 and ALG-2, our results suggest that miR-60 could either act in other cell types, where it is not normally active to promote recovery from starvation, or spread to the intestine from distal tissues via a cell non-autonomous action similar to that recently reported for miR-83^43^.

### ALG-2 is required in the nervous system for widespread changes in the translatome

In addition to providing information on miRNA function, our results are also useful in revealing the biological activities of individual AGOs. Our data indicate that an increased proportion of miRNAs are associated with ALG-2 in neurons when compared with intestinal cells and muscle cells (Figs. 2c and 3a). Indeed, while we observed that the expression of GFP::ALG-1 was largely ubiquitous, RFP::ALG-2 was enriched in the nervous system of late larval and adult stages (Supplementary Fig. 12), consistent with previous findings^16,17^. Global misregulation of miRNA target regulation has previously been observed in animals deficient in *alg-1* but not *alg-2*, suggesting that ALG-1 serves as the primary AGO for the miRNA pathway during development, with the contribution of ALG-2 being mostly redundant^44,45^. However, because our results suggested that ALG-2 may play an important regulatory role in neurons, we investigated the impact on the neuronal translatome in animals lacking *alg-2*.

**Fig. 7:**
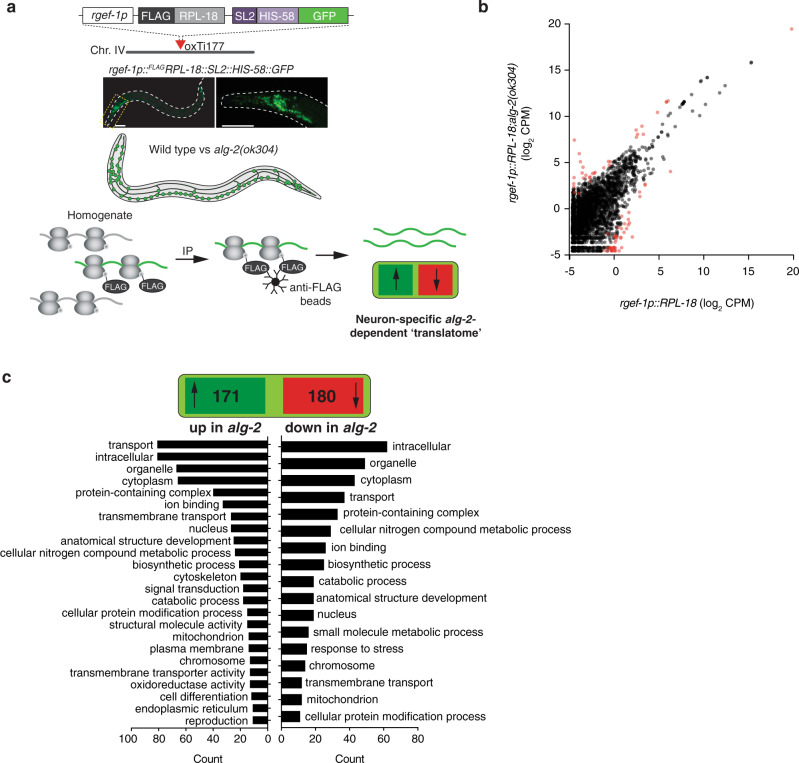
Specific effects of *alg-2* on the neuronal “translatome”. **a** Schematic representation of cell-type-specific polysome immunoprecipitation setup. Single copy FLAG-tagged RPL-18 protein driven by the neuron-specific *rgef-1* promoter allows purification of neuron-specific translating mRNAs from either wild-type or *alg-2* mutant backgrounds. Scale bars, 50 µm. **b** Scatterplot showing expression levels (log_2_ CPM, counts per million) of mRNAs immunoprecipitated with polysomes from neurons in *alg-2* mutant compared to wild-type worms. Red points represent significantly differentially upregulated or downregulated genes with a FDR value of <0.01. Fold change and significance was calculated by fitting a two-sided negative binomial model to processed read counts, according to edgeR pipeline for pairwise comparisons between multiple groups. **c** Broad gene ontology categorization of mRNAs upregulated (left) or downregulated (right) in neurons of *alg-2(ok304)*.

We adopted a polysome immunoprecipitation approach (Fig. 7a) in which we engineered strains expressing a FLAG epitope-tagged version of RPL-18 (a component of the small subunit of 80S ribosomes and polysomes) under the control of the neuron-specific promoter *rgef-1p* (Fig. 7a). MosSCI was used to generate single-copy insertions of the transgene and we confirmed pan-neuronal expression of the construct by following *SL2::his-58::GFP* fluorescence (Fig. 7a). After immunoprecipitation from populations of whole animals, mRNAs actively translating exclusively on neuronal polysomes were purified and sequenced. Differential analysis comparisons between wild-type and *alg-2(ok304)* backgrounds revealed that 171 polysome-associated transcripts were upregulated, whereas 180 were downregulated (Fig. 7b, c). The vast majority of these had known neuronal expression and functions (Supplementary Data 6). Gene ontology (GO) term analysis indicated a broad range of functional categories of both upregulated and downregulated transcripts (Fig. 7c and Supplementary Data 6) suggesting that ALG-2, via the miRNA interactions we established in neurons, markedly influences neural gene-regulatory networks in a wide range of cellular functions.

## Discussion

The development of an embryo into a complex multicellular organism requires the setting of specific transcriptional networks that not only direct the development of, but also maintain the correct identity and function of specific cell-types under a multitude of environmental influences. MiRNA-mediated repression is a central mechanism of gene regulation, that can direct and more often fine-tune these networks at the post-transcriptional level. Typically, genome-wide temporal miRNA expression profiles are achieved at the whole organism level, with spatial information then incorporated on a case-by-case basis through detailed expression studies of individual miRNA reporters that are driven by putative promoter sequences. However, these approaches are laborious and assume that (i) the *cis*-regulatory sequences and transgenic methods adopted recapitulate endogenous expression, and (ii) miRNA expression correlates precisely with loading and thus potential function. Post-transcriptional regulation of miRNA biogenesis, stability, and AGO loading, represent points at which expression and activity may diverge^31,32^. One recent approach to overcome many of these issues was the use of cell-specific expression of the *Arabidopsis* methyltransferase Hen-1 to methylate, and therefore chemoselectively protect and facilitate cloning of miRNAs for high-throughput sequencing^19^. This highly sensitive approach yielded valuable insights into the cell-type-specific abundance of mature miRNAs in *C. elegans*, but was not able to define those that constitute a silencing complex and are therefore presumed to be active. Physical isolation of cells by either laser dissection or fluorescence activated cell sorting followed by miRNA sequencing^20,23,46,47^ is also limited in its inability to resolve miRNA loading under non-invasive conditions. In addition, although AGO pulldown experiments and subsequent analyses of miRNA associations have been performed at a whole-animal level^16–18,29^, subtle and likely biologically relevant interactions have been missed because of the lack of cellular resolution afforded by these approaches.

To obtain a more robust molecular understanding of miRNA biological function and regulation, we systematically probed cell-specific interactions between miRNAs and their AGO effector proteins, which constitute the central component of the terminal silencing complex. By implementing and expanding upon approaches that we previously used in *Arabidopsis* root cell layers^48^, we have combined AGO immunoprecipitiation with cell-type specificity to reveal miRNA loading in a range of cell and tissue types from whole animals. In addition to validating the robustness of this approach against intertissue contamination, we found that it was also highly sensitive and could detect distinct miRNA:AGO interactions originating from a single cell within the whole animal. Despite this, there are several potential caveat’s associated with a miRNA:AGO immunoprecipitation based approach to address cell-type specific miRNA loading patterns. The inherent background that we observed (i.e., detection of miRNAs in wild type animals upon immunoprecipitation), while addressed in this current work, could provide a barrier to the true potential of the technique. Additionally, although stable miRNA:AGO associations are likely to represent an active or poised miRNA state, it is possible that loaded miRNAs may themselves be subject to further regulation before initiating target recognition and repression. For example, the seed nucleotides of the guide strand are not readily accessible without considerable conformational changes within the Piwi-Argonaute-Zwille (PAZ) domain of AGO proteins^49^. It also remains a possibility that the AGO proteins, which initially load miRNAs in their duplex form, do not automatically return to a conformational ground state that promotes expulsion of the non-guide strand (miRNA*) to form the mature silencing complex^50,51^. However, we observed a strong bias for guide strand loading in our datasets, indicating that the majority of miRNA:AGO complexes detected were based on single-stranded and therefore actively loaded miRNAs. As such, and to our knowledge, this represents the most precise and direct map of cell-type-specific miRNA loading at a genome-wide scale in animals. Indeed, the resolution provided by cell-type-specific analyses is exemplified by the discovery of hundreds of isomiRs and 37 new candidate miRNAs, as well as their spatial and AGO-loading patterns. This essentially reveals that even in one of the most well-characterized models of miRNAs^52,53^, a multitude of hidden and rich layers of miRNA biology remain to be elucidated.

Importantly, our results demonstrate that AGO binding itself can be uncoupled from the cellular abundance of mature miRNAs, representing experimental evidence of a layer of miRNA regulation that can spatially segregate activity. Indeed, studies have shown that the availability of AGOs is limited, and that at any time in a cell there is a several-fold excess of unbound miRNAs relative to miRNA:AGO silencing complexes^54–56^. Comparisons between tissue-specific mature miRNA abundance^19^ and tissue-specific miRNA:AGO loading (Supplemental Data 2) suggest that this is also true in nematodes. Moreover, our results reveal preferential interactions between specific miRNAs and either of the two AGO types within the same cell type at the same time (Supplemental Data 2), suggesting another dimension of miRNA regulation directed by AGO selection that was previously too subtle to appreciate in whole animal studies. Interestingly, in other species, such as *Drosophila* and potentially mammals, the identity of the AGO protein associated with a miRNA can dictate the mechanism that will lead to mRNA repression^57^. This is also true in *C. elegans* where the assembly of different miRNA RISCs (miRISCs) in the germline and soma affect targeted mRNAs distinctively^58^. In particular, somatic ALG-1 has been shown to regulate both mRNA stability and/or translation whereas ALG-2 appears to act more exclusively through translational repression^58^. Although ALG-1 and ALG-2 are highly similar in sequence and structure, they appear to form distinct protein complexes in vivo^29^. For example, ALG-1, but not ALG-2, can interact with the receptor for activated C-kinase (RACK1), which mediates miRISC recruitment to polysomes, the active site of translation^59,60^. This suggests that distinct miRNAs, via their preferential interaction with either ALG-1 or ALG-2, can direct distinct mRNA repression mechanisms within a specific cellular context. However, this system still appears to be intrinsically flexible if, for instance, one of the AGO proteins is either not expressed, degraded or downregulated. These distinctions also appear to be dynamic over time and could underlie biological purpose. For instance, the gradual decline in ALG-1 protein levels during ageing and concomitant increase in miRNA:ALG-2 associations, suggest a possible biological function for this exchange across lifelong physiological transitions. This could be purposely coupled with the primarily translational inhibitory function of ALG-2^58^ to afford fast, yet reversible post-transcriptional gene regulation during ageing where longer-term changes in gene expression, such as those invoked by developmental cues, are no longer needed. Indeed, it is suspected that modes of post-transcriptional regulation used by miRNAs in early embryogenesis differ from those used in late embryogenesis in a range of species^61–63^. The apparent functionality of AGO selection is also revealed when making comparisons between somatic tissue types. In neurons, we showed that ALG-2 is more abundant and associates proportionately with more miRNAs than in intestinal and muscle cells, when compared to ALG-1. Although it is not clear why ALG-2 is especially relevant to the nervous system, we found that it is required to regulate, whether directly or indirectly, ribosome-associated mRNA levels of a wide array of genes in this tissue type.

Because of their multitarget potential, individual miRNAs can act pleiotropically in many biological processes, broadening while at the same time complicating our ability to define distinct functions. As demonstrated with miR-60 and to a lesser degree miR-75 (in addition to the other published examples crossed with our data), defining the cell-type-specific activities of miRNAs can guide interrogation of their biological function. Identifying their direct targets is a goal that can also be aided by knowledge of cell-type-specific miRNA activity. Despite many years of active research, algorithmic predictions, and the implementation of a variety of different biochemical and sequencing advances, the identification and confirmation of miRNA targets remains one of the primarily unresolved areas of miRNA research in metazoans. A combination of imperfect complementarity required for the short miRNA seed sequence, and the primary action of miRNAs on translation rather than exclusively mRNA stability provides additional challenges to the accurate prediction and confirmation of miRNA targets. The use of CLIP or CLASH techniques^64,65^ can provide great insight into miRNA:target associations, but at present, the lack of spatial resolution and high background associated with these techniques likely hinders their true value in deciphering and accurately predicting miRNA-mediated target regulation. Direct evidence of increased protein levels upon miRNA depletion are limited by case-by-case, single target analyses, the lack of full genome coverage, and cell-type-specific adaptations currently afforded by proteomic approaches^66–68^. Cell-type-specific polysome immunoprecipitation by itself could assay differences in steady-state translating mRNAs, either between wild-type and mutant backgrounds as we demonstrated here or by incorporating other cell types to distinguish cell-type-specific translatomes in the future. Despite potentially providing accurate cell-type-specific information on steady-state mRNAs, polysome immunoprecipitation may not accurately pick up miRNA targeting at the translational repression level. That being said, by adding a ribosome footprinting step to the technique^61,69,70^, combined with our miRNA:AGO loading map, we could, in principle, gain significant insights into miRNA target confirmation and downstream regulation at previously unattainable levels of resolution.

The transgenic toolkit of strains developed here could also be useful in assessing miRNA and AGO loading in a range of settings not tested in the current study, such as during specific developmental stages, ageing, or under a wide range of environmental conditions, providing insight into miRNA-directed biological processes. Moreover, the universal Mos1-mediated single-copy insertion vector backbones constructed for this study could be readily modified with alternative promoter sequences to achieve a genome-wide view of miRNA functionality in any cell or tissue of interest in *C. elegans*. A final more controversial area of miRNA biology, which could potentially be investigated by using an extended version of our data is that of miRNA movement. Although miRNA movement between developmentally distinct cell types has been recently demonstrated in animals^43^, it would appear, and indeed would be logical to presume, that this movement would be more frequent within a subset of cells in a defined tissue type^48,71^. A priori, derivations of our technique could be used to address these issues, and enhance the resolution of our understanding of the spatial and temporal dynamics of miRNAs in animals.

## Methods

### *C. elegans* stains and culture

Strains VT2084 *mals352[miR-71p::GFP*+*unc-119(+)]*, VT1665 *mals251[miR-1p::GFP; unc-119(+)]*, OH9729 *otIs302[lsy-6::GFP(fosmid)]*, VT1598 *mals227[miR-90p::GFP*+*unc-119(+)]*, VT621 *wwls16[miR-75p::GFP*+*unc-119(+)*, VT396 *wwEx29[miR-83p::GFP*+*unc-119(+)]*, VT1474 *mals177[miR-243p::GFP*+*unc-119(+)]*, MLC56 *lucEx43[miR-791(fosmid)::GFP*+*txx-3p::mCherry]*, VT1494 *maIs197[miR-234p::GFP*+*unc-119(+)]*, MT16471 *miR-60(n4947) II*, MT18037 *miR-75(n4472) X*, MT16311 *miR-77(n4286) II*, MT15022 *miR-83(n4638) IV*, MT12983 *miR-238(n4112) III*, MT15454 miR243(n4759) IV, VC446 *[alg-1 (gk214)]*, RB574 *[alg-2 (ok304)]*, PQ583 *alg-2(ap432[3xfag::mKate2::alg-2]) II; alg-1(ap423[3xfalg::gfp::alg-1])X* and wild-type (N2) were provided by the *Caenorhabditis* Genetics Center (CGC), which is funded by the National Institutes of Health (NIH) Office of Research Infrastructure Programs (P40 OD010440). Transgenic strains generated in this study are shown in Supplementary Table 1. *C. elegans* culture and maintenance were performed using standard techniques^72^.

### Molecular cloning

All plasmids were cloned using a modified version of pCFJ150 using either the 3-way gateway or standard PCR-based techniques. Promoter sequences (*eft-3p*, *ges-1p*, *rgef-1p*, and *myo-3*p) were amplified using the oligos listed in Supplementary Table 2 (eft3p-F and eft3p-R, ges-1p-F and ges-1p-R, rgef-1p-F and rgef-1p-R, myo-3p-F and myo-3p-R, unc-25p-F and unc-25p-R) and then subcloned into a modified restriction-compatible version of pDONR4-1r (Invitrogen). Full-length cDNA versions of *alg-1* and *alg-2* incorporating an N-terminal HA tag were amplified using the oligos listed in Supplementary Table 2, and cloned into the entry vector pDONR221 using gateway technology. A PCR product containing SL2:His-58:GFP was amplified using the oligos SL2-2-F and tbb2-3-R and recombined into the entry vector pDONR P2R-P3 using gateway technology. The subsequent entry clones (cell-specific promoters, HA:ALG1/2 and SL2:His58:GFP) were recombined into the destination vector modified pCFJ150. Subsequent plasmids pSZ77 (*eft-3p::HA::ALG-2::SL2::HIS-58::GFP::tbb-2 3*′*UTR*), pSZ83 (*ges-1p::HA::ALG-1::SL2::HIS-58::GFP::tbb-2 3*′*UTR*), pSZ86 (*rgef-1p::HA::ALG-1::SL2::HIS-58::GFP::tbb-2 3*′*UTR*), pSZ89 (*unc-25p::HA::ALG-1::SL2::HIS-58::GFP::tbb-2 3*′*UTR*), pSZ92 (*myo-3p::HA::ALG-1::SL2::HIS-58::GFP::tbb-2 3*′*UTR*), pSZ78 (*eft-3p::HA::ALG-2::SL2::HIS-58::GFP::tbb-2 3*′*UTR*), pSZ84 (*ges-1p::HA::ALG-1::SL2::HIS-58::GFP::tbb-2 3*′*UTR*), pSZ87 (*rgef-1p::HA::ALG-2::SL2::HIS-58::GFP::tbb-2 3*′*UTR*), (unc-25p*::HA::ALG-2::SL2::HIS-58::GFP::tbb-2 3*′*UTR)*, and pSZ93 (*myo-3p::HA::ALG-2::SL2::HIS-58::GFP::tbb-2 3*′*UTR*) were sequenced to authenticate.

pSZ180 (p*rgef-1p::FLAG::RPL-18::SL2::HIS-58::GFP::tbb-2 3*′*UTR*) was cloned by amplifying RPL18 with an N-terminal FLAG tag using the oligos F-RPL18-F and RPL18-SL2-R, and substituting the HA:ALG-1 sequence of pSZ86 using this amplicon and the oligos F-RPL18-F and rgef-1p-R.

pSZ176 (ALG1::FP-SEC-pDD282) was generated essentially as described in the ref. ^73^ using the oligos CB-76 and CB-77 for the 5′ HDR and the oligos CB-78 and CB-79 for the 3′ HDR. Both fragments were cloned in the *Spe*I digested vector pDD282 using Gibson assembly.

pSZ178 (*alg-1*::sgRNA) was generated using reverse PCR-based amplification using the oligos Cas9-sg-ALG1-F and Cas9-sg-ALL-R on the template vector pDD162 to yield a sg-RNA with the sequence; AGCGCUUUCAAUCCCUCUCAUGG.

pSZ246 (*elt-2p::mir-60::SL2:HIS-58::GFP::tbb-2 3*′*UTR)* was cloned using pSZ247 as a template for reverse PCR using the oligos CD-3 and pri-miR-60-F. This plasmid was subsequently digested with *Not*I and *Nae*I to clone the *elt-2* promoter, which was amplified with the oligos elt-2p-NotI-F and elt-2p-NaeI-R.

pSZ247 (*rgef-1p::miR-60::SL2:HIS-S58::GFP::tbb-2 3*′*UTR*) was cloned by amplifying pri-miR-60 using the oligos pri-miR-60-F and pri-miR-60-SL2-R. This product was used to replace the HA::ALG-1 sequence of pSZ86 using the oligos pri-miR-60-F and rgef-1p-R.

pSZ248 (*myo-3p::miR-60::SL2:HIS-58::GFP::tbb-2 3*′*UTR*) was cloned by amplifying pri-miR-60 using the oligos pri-miR-60-F and pri-miR-60-SL2-R. This product was used to replace the HA::ALG-1 sequence of pSZ92 using the oligos pri-miR-60-F and myo-3p-R.

pSZ249 (*miR-67p::SL2::HIS-58::GFP::tbb-2 3*′*UTR*) was made by amplifying the putative promoter region ~4 kb upstream of the pri-miRNA with the oligos miR-67p-NotI-F and miR-67p-PacI-R and cloning into a modified version of the vector pSZ246, which contained a multicloning site upstream of the *SL2::HIS-58:GFP:tbb2* expression cassette (amplified with CD-3 and SL2-F) using *Not*I and *Pac*I.

pSZ256 was cloned by amplifying the upstream putative promoter region of miR-77 to the end of the stemloop with the oligos miR-77p-NotI-F and miR-77-NheI-R and cloning into a modified version of the vector pSZ246, which contained a multicloning site upstream of the *SL2::HIS-58::GFP::tbb-2 3*′*UTR* expression cassette (amplified with CD-3 and SL2-F), using *Not*I and *Nhe*I.

pSZ257 was cloned by amplifying the upstream putative promoter region ~4 kb upstream of of *mir-238* to the end of the stemloop with the oligos miR-238p-NotI-F and miR-238-NheI-R and cloning into a modified version of the vector pSZ246, which contained a multicloning site upstream of the *SL2::HIS-58::GFP::tbb-2 3*′*UTR* expression cassette (amplified with CD-3 and SL2-F), using *Not*I and *Nhe*I.

### Transgenic strains

DNA constructs were injected to generate single copy insertion lines using the mosSCI method^24^. A complete list of transgenic strains used in this study is provided in Supplementary Table 1. Transgene insertions were confirmed via genotyping using the oligos sz-6, sz-13, and sz-14 (Supplementary Table 2).

To make the CRISPR-Cas9 strain SJZ845 (*3xflag::GFP::alg-1*), young adult N2 worms were injected with the following mix: 10 ng/µL pSJZ176 (*alg-1::FP-SEC-pDD282*), 50 ng/µL pSJZ178 (*alg-1::sgRNA*), 10 ng/µL pGH8 (*rab-3p::mCherry*), 5 ng/µL pCFJ104 (*myo-3p::mCherry*), and 2.5 ng/µL pCFJ90 (*myo-2p::mCherry*). Subsequent selection steps were performed as described by Dickenson et al.^73^. The strain was backcrossed two times to N2 to generate the strain SJZ845. The pSZ78 and pSZ83 constructs were injected into the gonad of N2 worms at a concentration of 10 ng/µL together with the co-injection marker *odr-1p::DsRed* (50 ng/µL) and subsequently crossed to the *alg-1(gk214)* mutant to generate the extrachromosomal array lines SJZ1036 and SJZ1037. The plasmids pSZ249, pSZ256 and pSZ257 were injected into the gonads of N2 worms at a concentration of 10 ng/µL together with the co-injection marker *odr-1p::DsRed* (50 ng/µL) to generate the lines SJZ769, SJZ1048 and SJZ1051, respectively. PCR fusions containing the putative promoter of the novel miRNAs shown in Fig. 5 were generated by fusion PCR as described above and injected at a concentration of 5 ng/µL together with the co-injection marker *odr-1p::DsRed* (50 ng/µL) to generate the lines SJZ783 (S00855633), SJZ786 (S01133666) and SJZ832 (S00703534).

### ALG immunoprecipitation

Immunoprecipitation of miRNA:AGO complexes was performed essentially as described by^49^ but adapted for *C. elegans*. Briefly, approximately 50,000–100,000 synchronized L4-staged worms were grown on 4 × 100 mm nematode growth medium (NGM) plates seeded with OP50 *Escherichia coli* bacteria. Worms were harvested and washed three times in 10 mL of M9 buffer in a 15 mL falcon tube. Samples were briefly centrifuged at ~500×*g* for 2 min, before the supernatant was removed. The worm pellets were then flash frozen in liquid N2 and stored at −80 °C or used immediately for subsequent experiments. Worm pellets were ground to a fine powder in liquid N2, resuspended and lysed in 2–3 v/v of immunoprecipitation buffer (IP buffer; 50 mM Tris-HCl, pH 7.5, 150 mM NaCl, 10% glycerol, 0.1% NP40) containing 1 tablet/10 mL complete protease inhibitor cocktail (Roche) for ~10 min with intermittent mixing by inversion. All subsequent steps were performed at 4 °C. Lysates were cleared by centrifugation at 14,000×*g* for 10 min. Cleared lysates were normalized by protein quantification using a modified Lowery procedure with the DC^TM^ Protein Assay Kit (Bio-Rad). Five to ten percent of this lysate was kept as input fraction. Lysates were then pre-cleared with 15 µL of protein A/G magnetic beads (Pierce Scientific) for 1–2 h with rotation at 4 °C. Fifteen microliter of either FLAG or HA (depending on transgenic strain background) conjugated beads were subsequently added to the mixture, followed by 2–3 h incubation with rotation at 4 °C. The beads were washed two times for 15 min each in ice cold IP buffer, followed by two subsequent washes in “high salt” IP buffer (containing 300 mM NaCl), also for 15 min each. After these washes, TRIzol reagent (Invitrogen) was added to the beads and RNA extracted from the aqueous phase and protein from the organic phase, according to manufacturer’s instructions. For FLAG-tagged CRISPR lines, immunocomplexes were eluted by vigorous shaking in 150 µL of lysis buffer supplemented with 200 ng/µL of FLAG peptide (Sigma) at 4 °C for 30 min. RNA and protein were subsequently extracted as described above.

### Small-RNA sequencing and analysis

AGO-bound RNA extracted using TRIzol reagent (Invitrogen) was processed into sequencing libraries using adapted Illumina protocols and sequenced at the ETH Functional Genomics Center using the Illumina NextSeq 500 sequencer. Raw small-RNA-seq data were processed by clipping 3′ adapters (TGGAATTCTCGGGTGCCAAGG) and filtering based on expected miRNA read length (18–30 nt) using cutadapt (v2.10)^74^. Small-RNA reads were subsequently mapped to the *C. elegans* reference genome (WBcel235) with bowtie2 (v2.3.4.3)^75,76^, and annotated to known mature miRNAs (miRbase, v21) using FeatureCounts (Subread v2.0.0)^77^. Pairwise comparisons of miRNAs between argonautes and tissues were performed with edgeR (v3.11)^78,79^ using default normalization and dispersion parameters. To control for lowly-expressed reads, only miRNAs with cpm ≥ 1 in at least two independent libraries were considered for statistical analysis.

Novel candidate miRNAs were annotated using miRDeep2 (v2.0.0.8) as described^80^. To ensure robust identification of novel miRNAs, only candidates with a valid pri-miRNA hairpin structure and score > 3 were considered for further validation and analysis. Novel miRNA target prediction was performed by running miRanda (v3.3a)^81^ in a local environment (Ubuntu 16.04.5 LTS), where mature novel miRNA sequences were compared to the *C. elegans* reference genome (WBcel235). Hits were validated by observing binding energy and base matching between 5′ region of novel miRNAs and known *C. elegans* mRNAs.

### RNA gel blot analysis

Total or immunoprecipitated RNA was separated on 17.5% polyacrylamide-urea denaturing gels, then transferred to Hybond-NX nitrocellulose membranes (GE Healthcare), and chemically cross-linked via 1-ethyl-3-(3-dimethylaminopropyl) carbodiimide-mediated cross-linking^81^. Oligonucleotides used for probes were complements of the respective miRNA sequences, and were end-labeled using T4 PNK (Thermo Scientific) with [γ-32P] dATP. The sequences of all probes are listed in Supplementary Table 2.

### Real-time qRT-PCR analysis

Total (input) or AGO-immunoprecipitated RNA was reverse transcribed^82^. Briefly, 100–500 ng of input or IP RNA was reverse transcribed in a final volume of 10 µL containing 2 µL 5× ProtoScript II reaction buffer (NEB), 25 µM ATP, 25 µM dNTPs, 50 µM RT primer (IK-44), 1 unit of poly(A) polymerase (Invitrogen) and 20 units of protoscript II reverse transcriptase (NEB). Reactions were incubated at 42 °C for 1 h followed by enzyme inactivation at 95 °C for 5 min. Real-time quantitative reverse transcriptase PCR (RT qPCR) was performed using a LightCycler 480 II (Roche) with SensiFAST SYBER No-ROX (Bioline Meridian Biosystems) using the gene-specific primers listed in Supplementary Table 2. PCR was carried out in technical triplicates using the following cycling conditions: 95 °C for 3 min, followed by 45 cycles of denaturation at 95 °C for 10 s, annealing at 60 °C for 10 s, and elongation at 72 °C for 20 s. A melting curve was generated at the end of the amplification in every run to confirm primer specificity. Threshold cycle (*C*_t_) values were determined by calculating the second derivative maximum of three technical triplicates for each sample. Data were analysed using Prism-GraphPad Software v8.4.0.

### Western blot analysis

Total proteins were extracted via lysis during immunoprecipitation experiments or by boiling 50–100 staged worms in 1× sample buffer. Proteins were resolved on SDS-PAGE gels, and electro-transferred to Immobilon-P PVDF membranes (Millipore). After blocking for 30 min in 1× PBS + 0.1% Tween-20 supplemented with 5% skim milk powder or 3% BSA, subsequent antibody incubations were carried out overnight at 4 °C in the same solution. Primary anti-HA (Sigma H6533) and anti-FLAG (Sigma F3165) antibodies were diluted 1/5000. Membranes were washed four times in 1× PBS + 0.1% Tween-20, and then incubated for 1 h at room temperature with horseradish peroxidase-conjugated goat anti-rabbit (Abcam ab6721) or goat anti-rat (Cell Signaling 7077S), diluted 1/10,000. After washing again four times in 1× PBS + 0.1% Tween-20, detection was performed using the ECL Western Blotting Detection Kit (GE Healthcare) and revealed either by exposure to film or using the ChemiDoc ^TM^ Touch imaging system (Bio-Rad). Equal loading was confirmed either by using alpha-tubulin (Sigma T6074) as described above diluted 1/5000, or by staining the membranes with Coomassie blue.

### Polysome immunoprecipitation

Immunoprecipitation of pan-neuronal polysome complexes was performed essentially as described in the ref. ^49^ but adapted for *C. elegans*. Briefly, approximately 200,000–300,000 synchronized L4-staged worms were grown on 8–10 × 100 mm NGM plates seeded with OP50 *E. coli* bacteria. Worms were harvested at the L4 stage and washed three times in 10 mL of M9 buffer in a 15 mL falcon tube. Centrifuged worm pellets were then flash frozen in liquid N2 and stored at −80 °C or used immediately for subsequent experiments. Worm pellets were ground to a fine powder in liquid N2, resuspended and lysed in 2–3 v/v of polysome extraction buffer (PEB; 20 mM Tris-HCl pH 7.9, 140 mM KCl, 1.5 mM MgCl_2_, 0.5% NP40, 2% PTE (polyoxyethylene 10 tridecyl ether), 1% sodium deoxycholate, 1 mM DTT, 25 mM EGTA, 50 µg/mL cyclohexamide) containing 1 tablet/100 mL complete protease inhibitor cocktail (Sigma) for ~20 min with intermittent mixing by inversion. Lysates were cleared by centrifugation for 10 min at 14,000×*g* at 4 °C. All subsequent steps were performed either on ice or at 4 °C unless otherwise stated. Lysates were then incubated with 10–15 µL of washed protein A/G magnetic beads with rotation for 1–2 h. Pre-cleared lysates were incubated with 15 µL of anti-FLAG M2 magnetic beads (Sigma M8823) for 2–3 h with rotation. Immunocomplexes were washed once in PEB for 15 min and then three subsequent times each for 15 min in wash buffer (20 mM Tris-HCl pH7.9, 140 mM KCl, 1.5 mM MgCl_2_, 0.1% NP40, 1 mM DTT, 25 mM EGTA, 50 µg/mL cycloheximide) containing 1 tablet/100 mL complete protease inhibitor cocktail (Sigma). Polysome:mRNA complexes were eluted by vigorous shaking in 150 µL of wash buffer (20 mM Tris-HCl pH 7.9, 140 mM KCl, 1.5 mM MgCl_2_ 1 mM DTT, 25 mM EGTA, 50 µg/mL cyclohexamide) at 4 °C for 30 min supplemented with 200 ng/µL of FLAG peptide (Sigma). RNA was extracted using TRIzol reagent as described above.

### mRNA polysome sequencing and analysis

Total RNA was isolated as described above during polysome immunoprecipitation and sequenced at GENEWIZ (http://www.genewiz.com, China) using adapted Illumina protocols. Libraries were prepared using ~1 ng of total RNA using NEBNext Single Cell/Low Input RNA Library Prep Kit for Illumina (NEB E6420S) and sequenced as paired-end 150 nt reads on an Illumina HiSeq 2000 platform. Raw mRNA-seq data were trimmed by clipping 3′ adapters (read 1: AGATCGGAAGAGCACACGTCTGAACTCCAGTCA; read 2: AGATCGGAAGAGCGTCGTGTAGGGAAAGAGTGT) using cutadapt (v2.10)^74^. Reads were further mapped to the *C. elegans* genome (WBcel235) with HISAT2 (v2.10)^82^ and annotated to known mRNAs (ensembl, release 96) using FeatureCounts (Subread v2.0.0)^77^. Differential expression between samples was performed using edgeR (v3.11)^78,79^ as described above in small-RNA-seq analysis. Gene ontology of significant mRNAs was annotated using biomaRt (2.46.0)^82,83^.

### Live imaging

Transgenic animals carrying fluorescent transcriptional or translational fusion reporters were immobilized in 1 mM of levamisole (Sigma) and mounted on a 2% agarose pad attached to a glass slide. Fluorescence was visualized using either a Zeiss Z2 imager microscope equipped with a Zeiss Axiocam 506 mono Camera with Zen2 (version 2.0.0.0) software or a Zeiss 780 confocal microscope. The images shown in all figures are representative of consistent results obtained in multiple independent experiments.

### Recovery from starvation assays

Gravid egg-laying adult animals were bleached and eggs allowed to hatch in M9 medium with rotation. L1 worms were starved for 72 h (or not, for non-starved animals) in M9 with rotation prior to seeding, and recovery on NGM plates seeded with OP50 *E. coli* and grown until wild type animals reached L4 (~40 h) or young adults (~48 h) at 20 °C. Body areas of either L4 or adult worms were measured after washing the animals onto NGM plates containing no food. Videos of ~5 s were recorded using a Nikon SMZ745T stereomicroscope and a TrueChrome IIS camera (Tucsen). Animal body area was quantified using WormLab tracking software (MBF Bioscience).

### Oil Red O staining

Starved L4 stage worms were collected in 1× PBS + 0.1% Tween-20 and washed a minimum of two times in the same solution. After the final wash worms were left in 400 µL of 1× PBS + 0.1% Tween-20 to which 500 µL of 2x MRWB (160 mM KCl, 40 mM NaCl, 14 mM EGTA, 0.4 mM Spermine, 30 mM PIPES (pH 7.4), 0.2% 2-mercaptoethanol) and 100 µL 20% paraformaldehyde were added. Fixation was performed with rotation for 1 h at room temperature. Fixed worms were subsequently washed twice in 1 mL of 100 mM Tris-HCl (pH 7.4). Worms were resuspended in 100 µL of 100 mM Tris-HCl (pH 7.4) and combined with 900 µL of reduction buffer (100 mM Tris-HCl (pH 7.4), 10 mM DTT). After mixing by inversion for 30 min, worms were washed once with 1× PBS and aspirated to 300 µL. Seven hundred microliter of isopropanol was added and samples were mixed with rotation for 1 h. Worms were pelleted and resuspended in Oil Red O solution (0.5 g Oil Red O in 100 mL isopropanol) diluted 1.5× in water and filtered through a 0.2 µM unit. Samples were incubated with rotation for 2 h at room temperature and then washed twice with 1× PBS + 0.1% Tween-20 prior to imaging. Quantification was performed using Image J fluorescence intensity on at least 10 independent worms.

### Reporting summary

Further information on research design is available in the Nature Research Reporting Summary linked to this article.

## Supplementary information

Supplementary information

Supplementary Data 1

Supplementary Data 2

Supplementary Data 3

Supplementary Data 4

Supplementary Data 5

Supplementary Data 6

Description of Additional Supplementary Files

Reporting Summary

## Data Availability

Raw sequencing data and supplementary files are available on the NCBI Gene Expression Omnibus (GEO) (G6). All other relavent data are available from the corresponding authors on request. Source data are provided with this paper.

## Code availability

A complete bioinformatic pipeline including all relevant shell and R scripts may be found at (https://github.com/Alexander-Palmer/smallRNA-polymRNA).
