# Supplementary material for: Cell-type-specific profiling of loaded miRNAs from Caenorhabditis elegans reveals spatial and temporal flexibility in Argonaute loading
Source: Nat Commun. 2021 Apr 13;12:2194. doi: 10.1038/s41467-021-22503-7 (PMC8044110; doi:10.1038/s41467-021-22503-7)
Supplement: Supplementary file 1 — Supplementary information [file 41467_2021_22503_MOESM1_ESM.pdf]

Cell-type-specific profiling of loaded miRNAs from *Caenorhabditis elegans* reveals spatial and temporal flexibility in Argonaute loading

Christopher A Brosnan[1,2,*], Alexander Palmer[1] and Steven Zuryn[1,*].

Supplementary Information:

Supplementary Figure 1
Supplementary Figure 2
Supplementary Figure 3
Supplementary Figure 4
Supplementary Figure 5
Supplementary Figure 6
Supplementary Figure 7
Supplementary Figure 8
Supplementary Figure 9
Supplementary Figure 10
Supplementary Figure 11
Supplementary Figure 12
Supplementary Figure 13
Supplementary Figure 14
Supplementary Figure 15

Supplementary Table 1
Supplementary Table 2

Supplementary Note 1

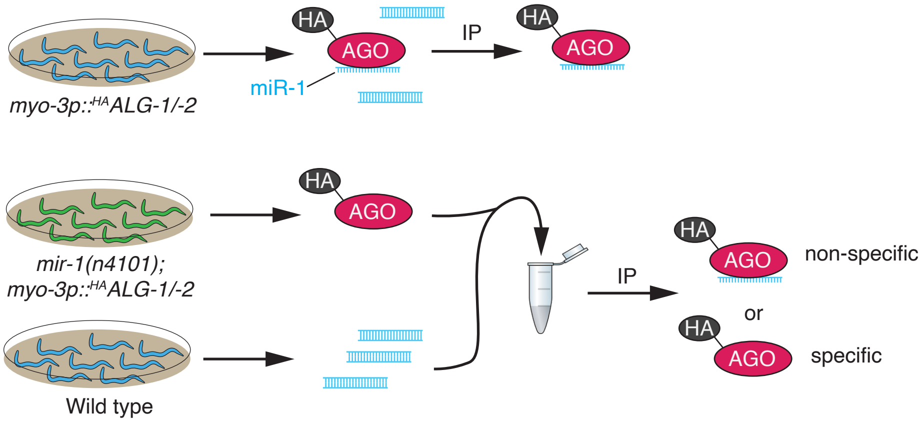

Supplementary Figure 1. Schematic representation of experimental design from Figure 1e to demonstrate intact and unaltered *in vivo* AGO:miRNA complexes during immunoprecipitation process. Standard AGO:miRNA IP is shown at the top with expected loading of miR-1 when either ^{HA}ALG-1 or ^{HA}ALG-2 is immunoprecipitated specifically from the body wall muscles. *mir-1(n4101)*;*myo-3p::^{HA}ALG-1/2* were homogenized and combined at a ratio of 1:1 with miR-1-expressing wild-type homogenates. After immunoprecipitation, the two possible scenarios are shown on the right as either non-specific or specific complexes. Figure 1e clearly demonstrates that specific complexes are formed.

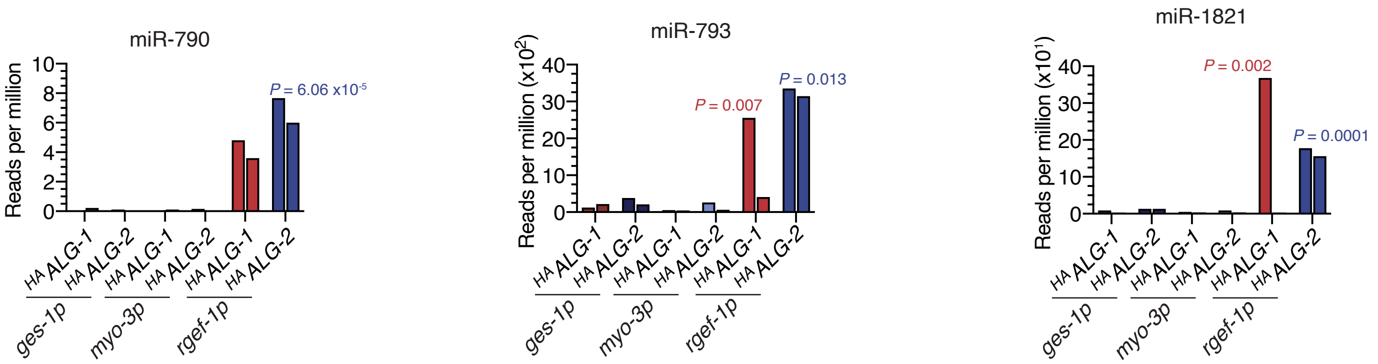

Supplementary Figure 2. Sensitivity of cell-type-specific AGO loading as illustrated in neurons. Plot of reads counts per million in individual cell types of miR-790 (left), miR-793 (middle) and miR-1821 (right) in either ALG-1 or ALG-2. Biologically independent replicate libraries are shown separately, two bars. *P* values are shown for significant loading and were calculated by fitting a two-tailed negative binomial model to processed read counts, according to edgeR pipeline for pairwise comparisons between multiple groups.

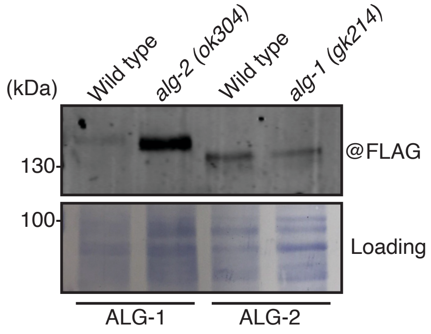

Supplementary Figure 3. Expression of ALG-1 and ALG-2 in corresponding mutant backgrounds. Western blot analysis of CRISPR generated $^{CRISPR\text{-}Cas9}$*3XFLAG::GFP::ALG-1* (ALG-1) in a wild-type or *alg-2*(*ok304*) mutant background. The same is shown for $^{CRISPR\text{-}Cas9}$*3XFLAG::RFP::ALG-2* (ALG-2) in a wild-type or *alg-1*(*gk214*) background. Loading represents total protein stained with Coomassie blue. ALG levels were confirmed by western blots on two separate occasions with identical results.

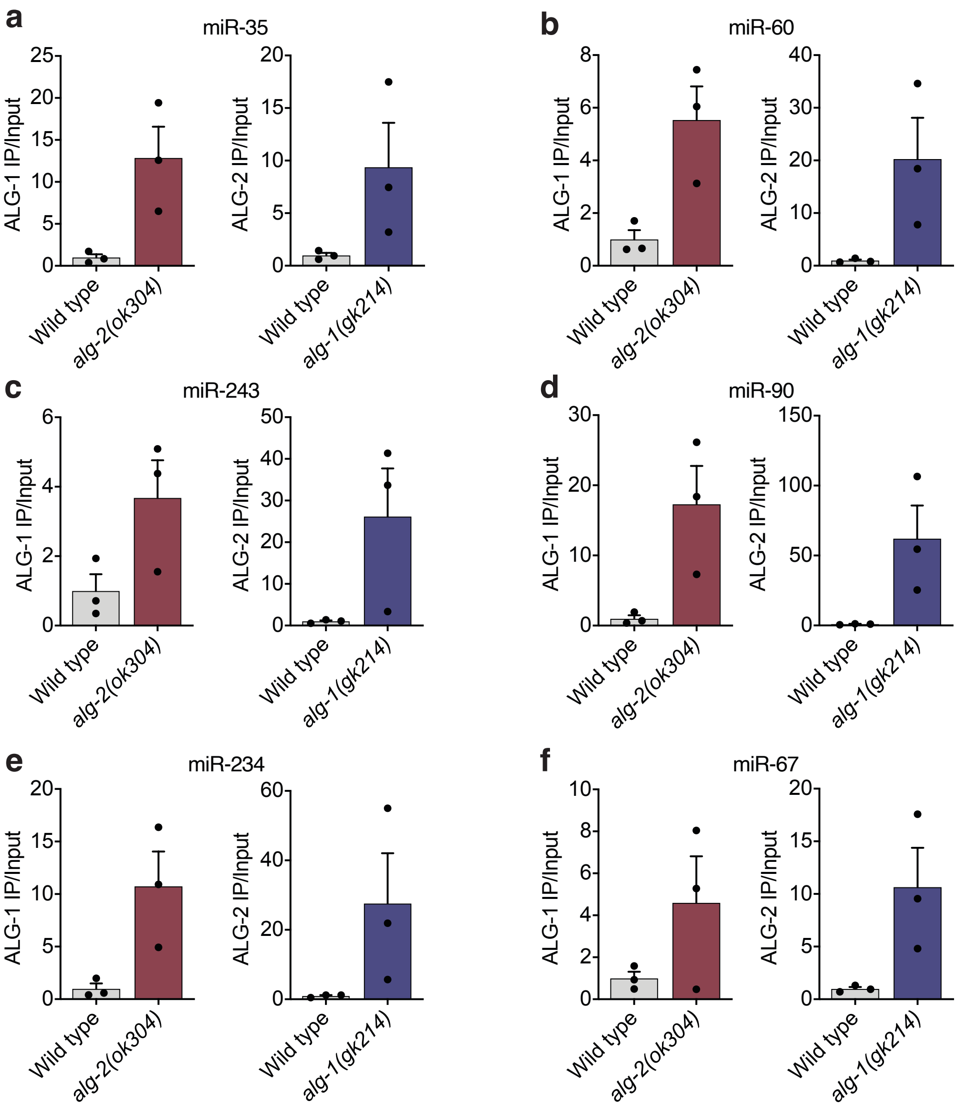

Supplementary Figure 4. Quantification of miRNA re-loading between ALG-1 and ALG-2. (a) Re-loading of the constitutively expressed miR-35 into ALG-1 in an *alg-2(ok304)* mutant (left – grey vs red) or into ALG-2 in an *alg-1(gk214)* mutant (right – grey vs blue). **(b)** Re-loading of the intestine-specific miRNA miR-60. **(c)** Re-loading of intestine-specific miR-243. **(d)** Neuronal-enriched miR-90 re-loading between ALG-1 and ALG-2. **(e)** Neuronal-specific miR-234 re-loading between ALG-1 and ALG-2. **(f)** Re-loading of muscle-specific miR-67. Each bar represents the average enrichment of immunoprecipitation over input for the indicated miRNA in the indicated genetic back-ground. Error bar represent +/- s.e.m. for 3 biological immunoprecipitation replicates.

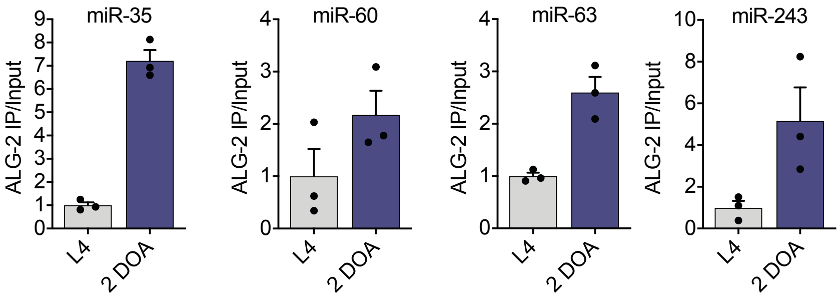

Supplementary Figure 5. Reloading of miRNAs into ALG-2 during ageing.
qRT-PCR of the indicated miRNAs in either ALG-2 immunoprecipitated L4 (grey) or 2-day-old adult (2 DOA) animals. Each bar represents the average enrichment of immunoprecipitation over input for the indicated miRNA at either L4 or 2 DOA animals. Error bar represent +/- s.e.m. for 3 biological immunoprecipitation replicates.

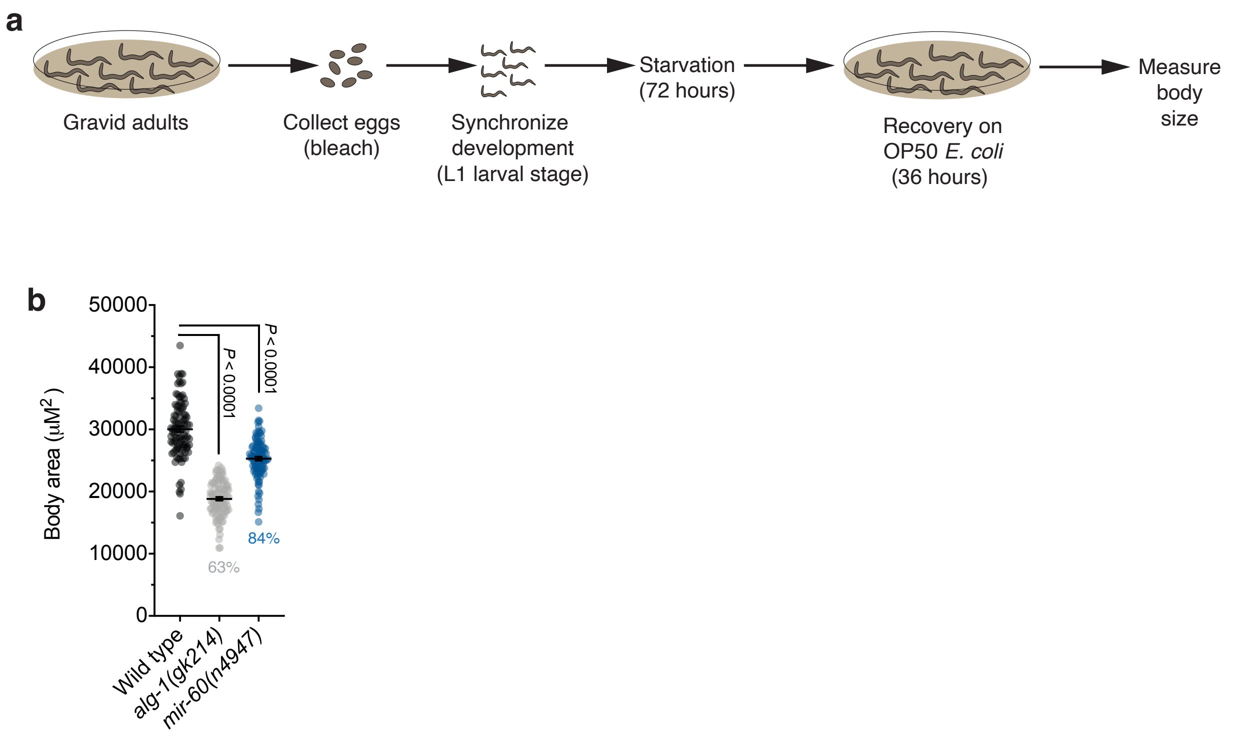

Supplementary Figure 6. Recovery from starvation stress. (**a**) Scheme of workflow used to assay recovery from starvation stress. Animals were hatched in M9 buffer and deprived of food for 72 hours. L1 larvae were then allowed to recover for ~36 hours prior to measuring body size. (**b**) Quantification of body area of non-starved animals (no 72 hour starvation period). Percentages shown are the relative average body areas of animals compared to wild type. Error bars represent +/- s.e.m. *P* values represent one-way ANOVA with Tukey's multiple comparisons test. n>30 biologically independent animals for each strain tested.

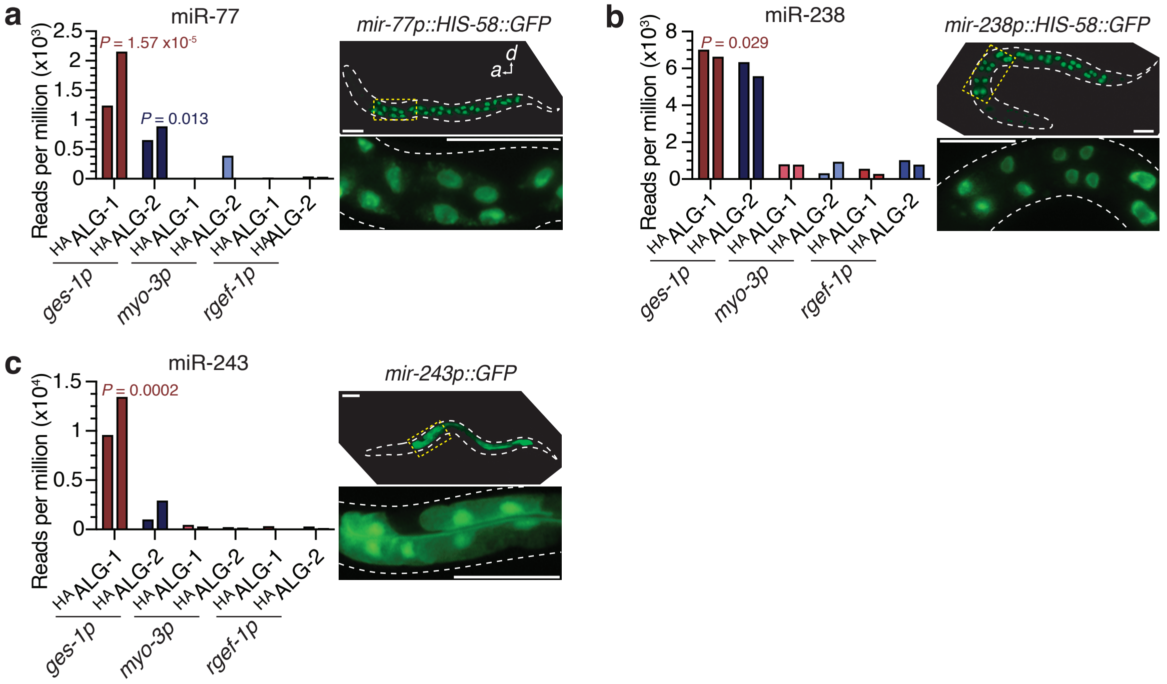

Supplementary Figure 7. Confirmation of intestine-expressed and AGO-loaded miRNAs. (a) Intestine-specific loading of miR-77 shown by plots of read counts per million in the indicated cell-type-specific AGO libraries (left). Promoter GFP fusion image (right) shows intestine-specific expression of miR-77. **(b)** Intestine-specific loading of miR-238 shown by plots of read counts per million in the indicated cell-type-specific AGO libraries (left). Promoter GFP fusion image (right) shows intestine-specific expression of miR-238. **(c)** Intestine-specific loading of miR-243 shown by plots of read counts per million in the indicated cell-type-specific AGO libraries (left). Promoter GFP fusion image (right) shows intestine-specific expression of miR-243. Graphs show two biological replicate experiments, two bars. Scale bars, 50 μm.

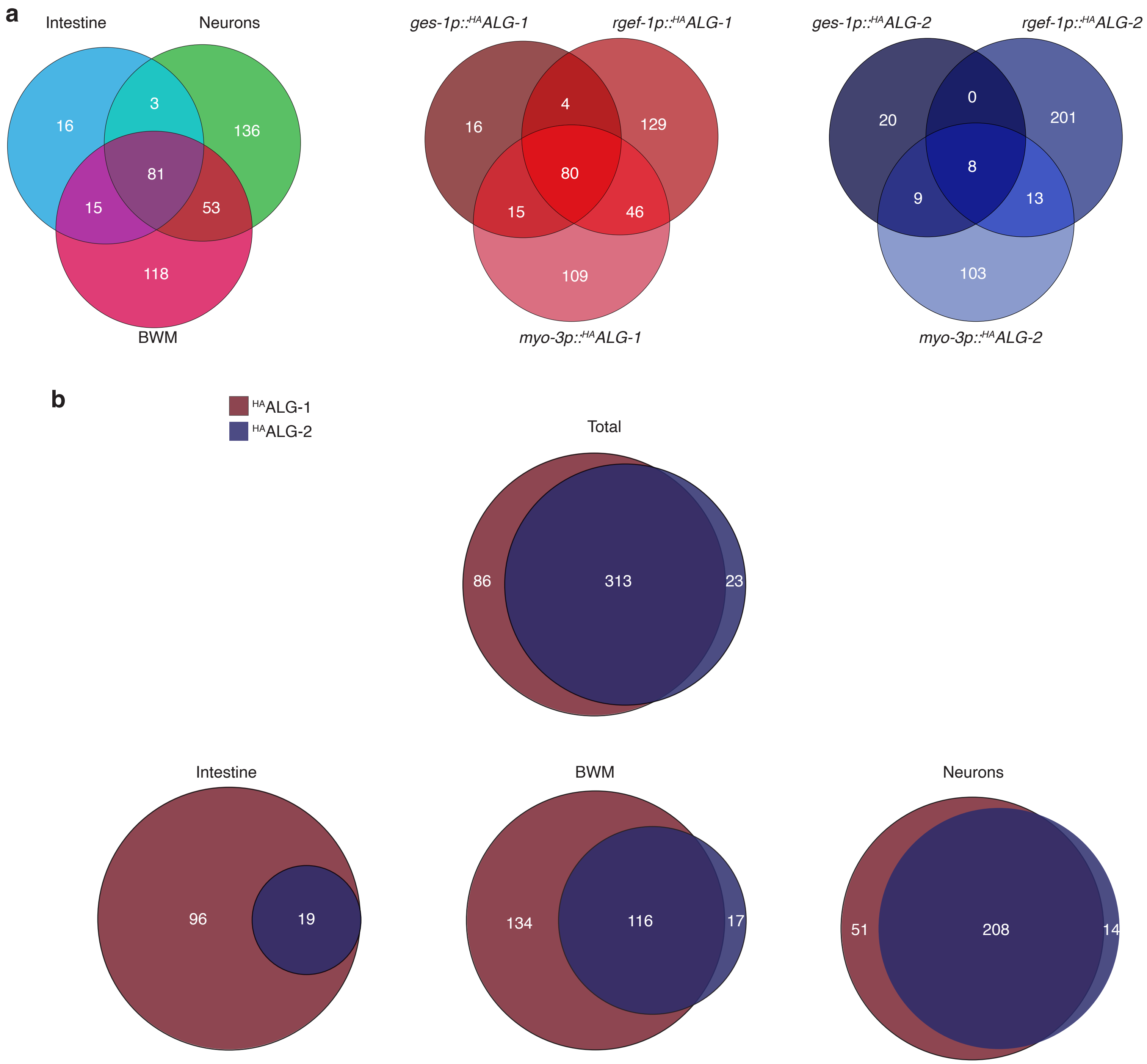

Supplementary Figure 8. Cell- and AGO-specific distribution of loaded isomiRs. **(a)** Venn diagram representation of cell-type specific isomiRs (left), ALG-1 loaded cell-type specific (middle) or ALG-2 loaded cell-type specific (right) isomiRs. **(b)** AGO-specific loading of isomiRs independent of cell-type (top) or AGO-specific loading with the indicated cell-types (bottom). BWM, body wall muscle.

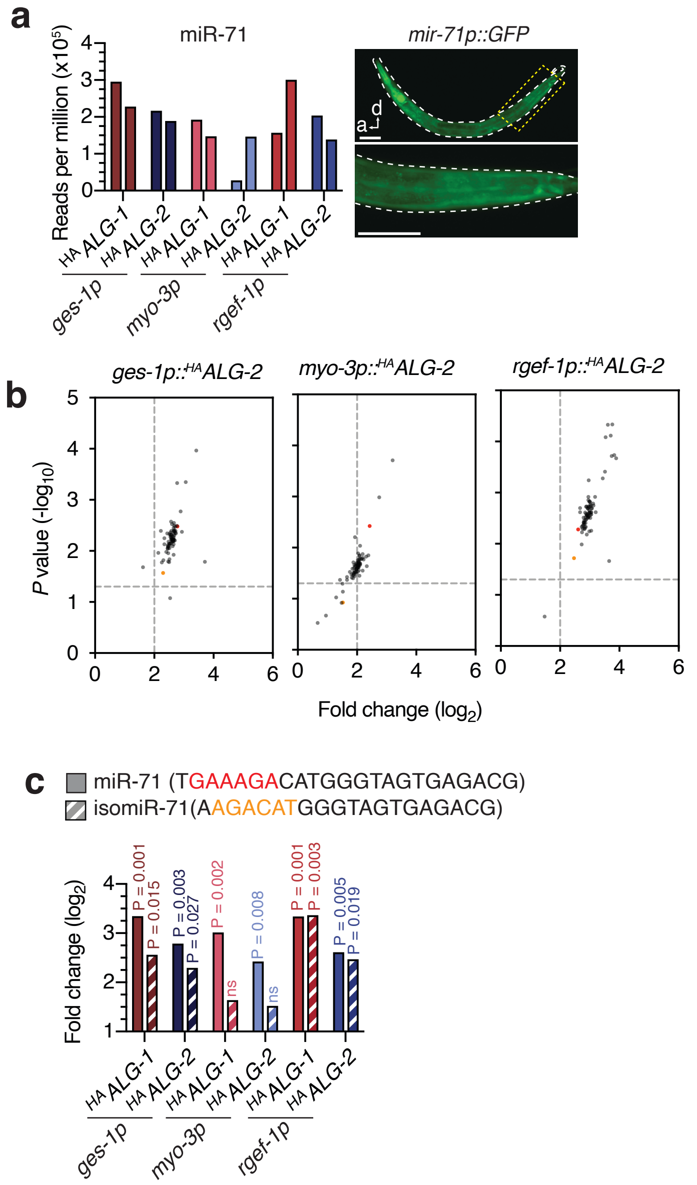

Supplementary Figure 9. miR-71 and isomiR variations in cell-type-specific loading. (a) Read counts per million in individual AGO- and cell-type-specific libraries for miR-71 showing ubiquitous enrichment. Live animal image (right) of the promoter of miR-71 fused to GFP showing ubiquitous expression. Scale bars, 50 µm. **(b)** Volcano plots of miR-71 (red) and miR-71 isomiRs (black and orange) in individual ALG-1- and cell-type-specific libraries. Orange dot corresponds to specific isomiR-71 shown in (c). P values were calculated by fitting a two-tailed negative binomial model to processed read counts, according to edgeR pipeline for pairwise comparisons between multiple groups. **(c)** AGO- and cell-type-specific log_2 fold changes of miR-71 and isomiR-71, which unlike miR-71 is not enriched in body wall muscle and has an alternative seed sequence (highlighted in red and orange font). For (a) and (c), graphs show two biological replicate experiments, two bars.

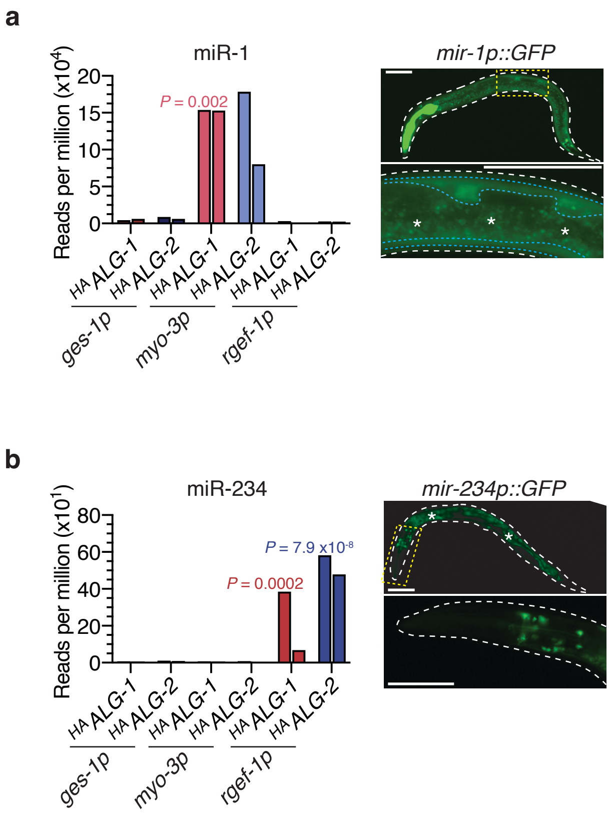

Supplementary Figure 10. Cell-type-specific loading indicates cell-type-specific function. (a) Muscle specific loading of miR-1 shown by plots of read counts per million in the indicated cell-type-specific AGO libraries (left). Promoter GFP fusion image (right) shows body wall muscle (outlined in dashed blue line in close up image), in addition to pharyngeal expression of miR-1. **(b)** Neuron-specific loading of miR-234 shown by plots of read counts per million in the indicated cell-type-specific AGO libraries (left). Promoter GFP fusion image (right) shows neuron-specific expression of miR-1. Graphs show two biological replicate experiments, two bars. Scale bars, 50 μm. Asterisks indicate intestine autofluorescence.

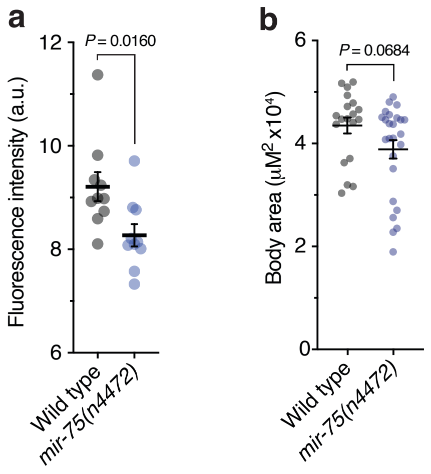

Supplementary Figure 11. Fat content and starvation recovery of *mir-75(n4472)*.
(a) Fat content of intestine of wild-type and *mir-75(n4472)* animals, as assessed by Oil Red O staining. a.u., arbitrary units. n=10 independent animals. **(b)** Body area measurement of starvation-recovered wild-type and *mir-75(n4472)* worms. n≥19 independent animals. Error bars represent +/- s.e.m. *P* values represent two-way unpaired Student's t-test.

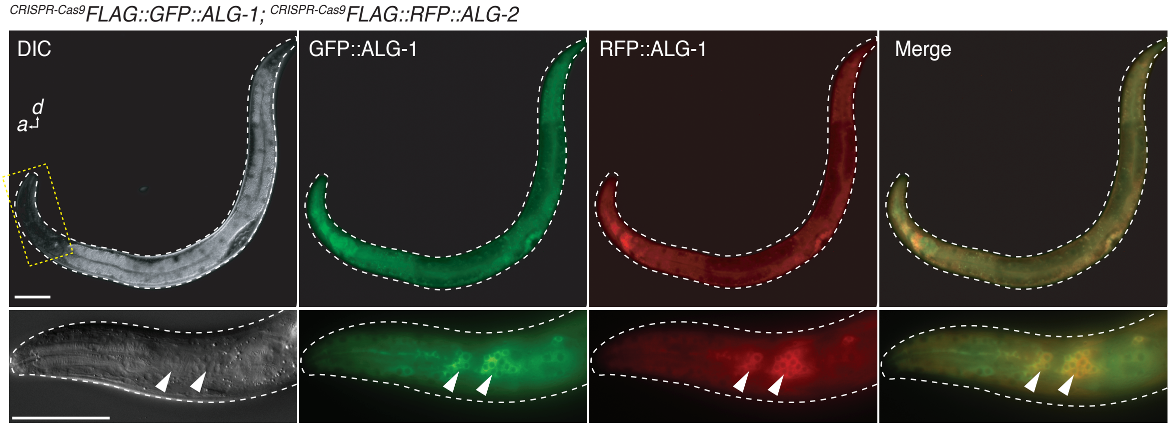

Supplementary Figure 12. Enrichment of ALG-2 expression in the neurons of the head. Representative confocal images of $^{CRISPR\text{-}Cas9}$*3XFLAG::GFP::ALG-1*; $^{CRISPR\text{-}Cas9}$*3XFLAG::RFP::ALG-2* transgenic L4 stage worms. Arrows indicate head ganglia. Scale bars, 50 µm. Images represent what was seen in at least 4 different imaged animals on multiple imaging sessions.

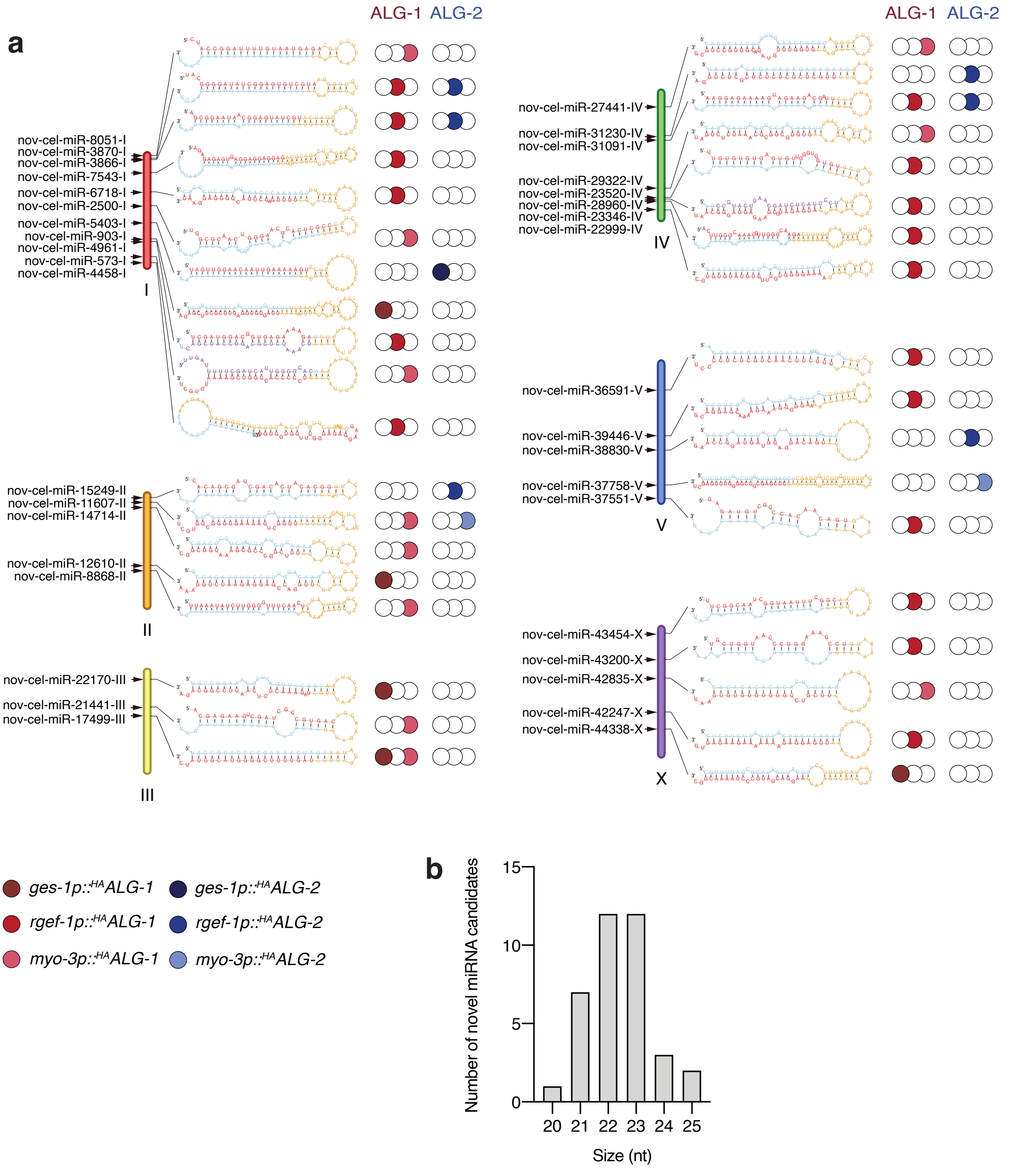

Supplementary Figure 13. Genomic location, pre-miRNA structure, cell-type-specific loading and AGO-specific loading of novel miRNA candidates. (a) Novel miRNA candidate name and genomic location (left). Predicted secondary structure of novel pre-miRNA transcript (middle). Colors (see key at bottom) indicate ALG-1 cell-type-specific (left) or ALG-2 cell-type-specific mature novel miRNA candidate loading patterns. **(b)** Size distribution of novel miRNA candidates.

Provisional ID : I_8051
Score total : 2.1
Score for star read(s) : -1.3
Score for read counts : 0
Score for mfe : 1.8
Score for randfold : 1.6
Score for cons. seed :
Total read count : 153
Mature read count : 153
Loop read count : 0
Star read count : 0

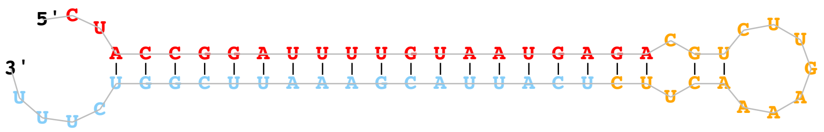

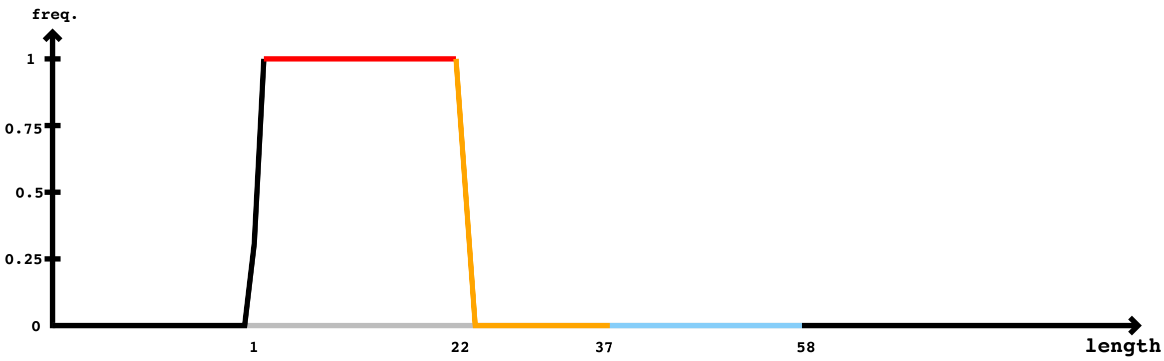

Mature Star

```
5'- uggaggcaaacuguccccaacuaccggauuuuguaaugagacgucuugaaaacuucucauuacgaaauucggucuuuuuggggcauuuuagucuaauagagcaaaaauuga   -3'    exp
    ...(((((((.((.((((((..(((((((((((((((((((.((.......)).)))))))))))))))))))....)))))))).))).)))).................   reads      mm          sample
    ...................acuaccggauuuuguaaugaga......................................................................   47         0           seq
    ....................cuaccggauuuuguaaugaga......................................................................   106        0           seq
```

Supplementary Figure 14. Stem loop structure, read counts and miRNA annotation as determined by miRDeep2 software of novel miRNA candidates.

Provisional ID	:	I_3870
Score total	:	2.6
Score for star read(s)	:	-1.3
Score for read counts	:	0
Score for mfe	:	2.3
Score for randfold	:	1.6
Score for cons. seed	:	
Total read count	:	244
Mature read count	:	244
Loop read count	:	0
Star read count	:	0

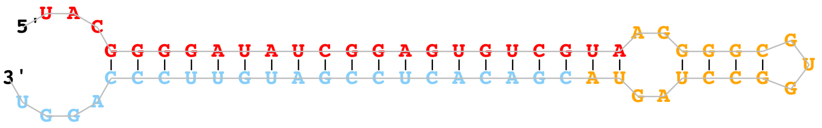

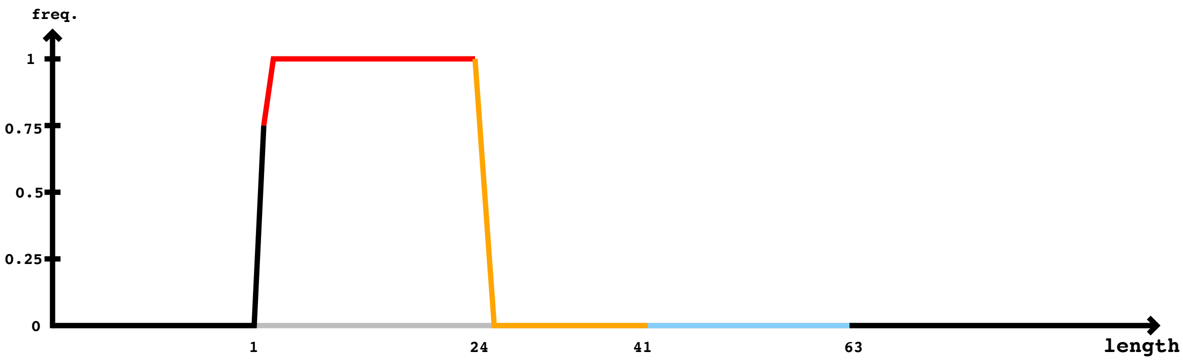

Mature Star

```
5'- ggaaaacauuggaaacuggcuacggggauaucggagugucguaaggggcguggccuaguacgacacuccgauguucccaggucuaaaauucgugaaaaguagugcuauucacu   -3'   exp
    ..........(....).((((..((((((((((((((((((((..((((...))))..))))))))))))))))))))).))))........(((((.((.....)).))))).   reads   mm   sample
    ....................uacggggauGucggagugucgua......................................................................   12      1    seq
    ....................uacggggauaucggagugucgua......................................................................   171     0    seq
    .....................acggggauaucggagugucgua......................................................................   61      0    seq
```

Supplementary Figure 14. Stem loop structure, read counts and miRNA annotation as determined by miRDeep2 software of novel miRNA candidates.

Provisional ID	:	I_3866
Score total	:	2
Score for star read(s)	:	-1.3
Score for read counts	:	0
Score for mfe	:	1.7
Score for randfold	:	1.6
Score for cons. seed	:	
Total read count	:	89
Mature read count	:	89
Loop read count	:	0
Star read count	:	0

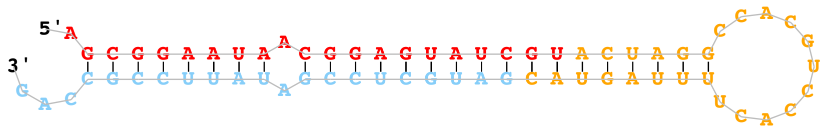

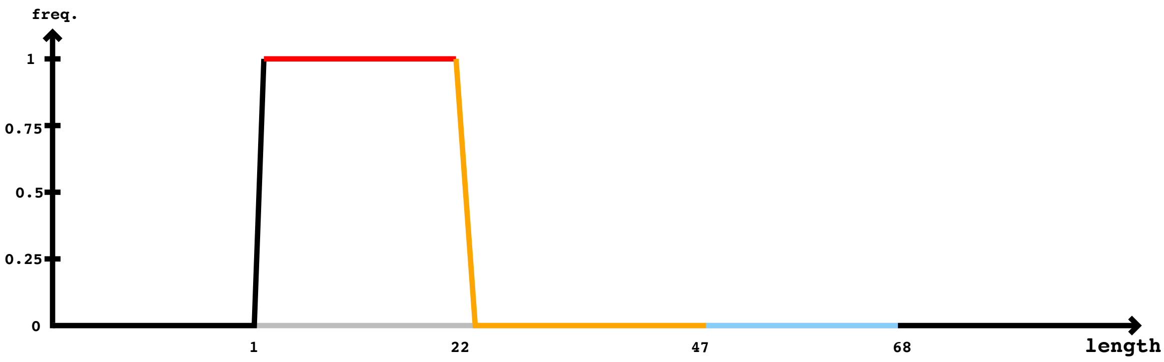

Mature   Star

```
5'- acuacaagaaaauuccagccagcggaauaacggaguaucguacuaggccacguccacuuuuaguacgaugcuccgauauuccgccaguaaauuuuaagaauuucaaaaaau   -3'    exp
    ........((((((...((..((((((((.(((((((((((((((((...........))))))))))))))))).))))))))..)).))))))................   reads    mm    sample
    ....................agcggaauaacggaguaucgu..........................................................................   89       0     seq
```

Supplementary Figure 14. Stem loop structure, read counts and miRNA annotation as determined by miRDeep2 software of novel miRNA candidates.

Provisional ID	:	I_7543
Score total	:	2.2
Score for star read(s)	:	-1.3
Score for read counts	:	0
Score for mfe	:	1.9
Score for randfold	:	1.6
Score for cons. seed	:	
Total read count	:	78
Mature read count	:	78
Loop read count	:	0
Star read count	:	0

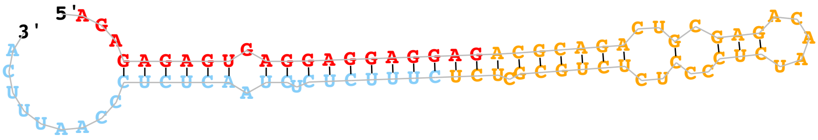

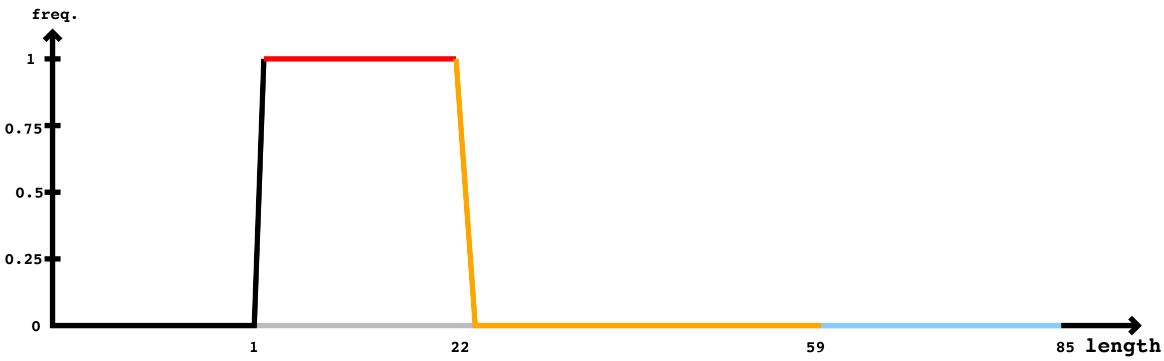

Mature **Star**

```
5'- aaacuugaaguucaagugcgagagagagugaggaggaggagacgcagacugcgagacaaucuccccucucugcgcucucuuucucucuaacucucccaauuucaaacccuc   -3'    exp
    ..(((((.....)))))..((((((((((.((((((((((((((((((..(.(((.....)))..)..)))))).)))))))))).)).))))))....))))........    reads     mm     sample
    ....................agagagagugaggaggaggag......................................................................    78        0      seq
```

Supplementary Figure 14. Stem loop structure, read counts and miRNA annotation as determined by miRDeep2 software of novel miRNA candidates.

Provisional ID	:	I_6718
Score total	:	2.1
Score for star read(s)	:	-1.3
Score for read counts	:	0
Score for mfe	:	1.8
Score for randfold	:	1.6
Score for cons. seed	:	
Total read count	:	250
Mature read count	:	250
Loop read count	:	0
Star read count	:	0

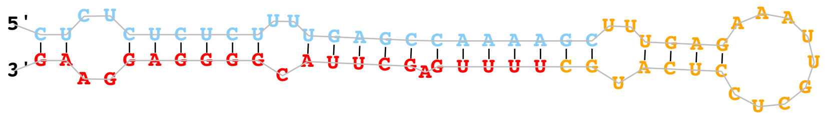

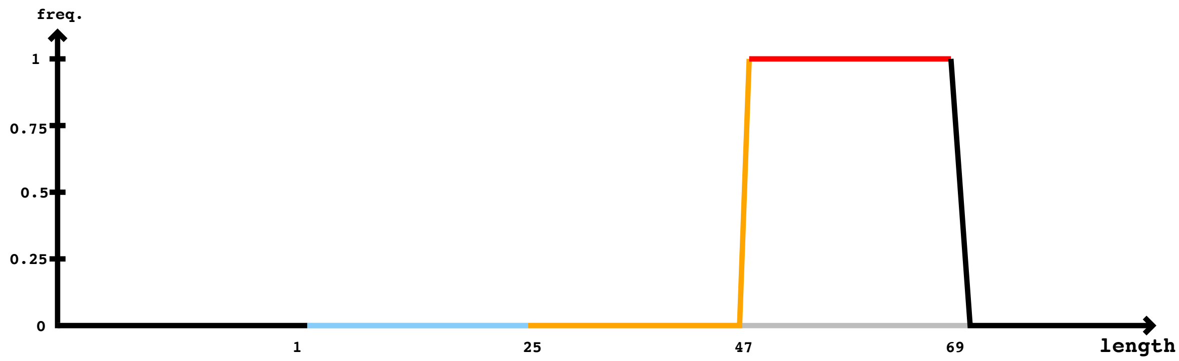

Star Mature

```
5'- gcagcagcagcaguauguugcucucucucucucuuuugagccaaaagcuuugagaaauugcuccucaugcuuuugagcuuacggggaggaaggcgucuauugaggaaguugc   -3'    exp
    ((((..(..(((((.(.(((.((..((((((....(((((((((((..((((.........)))).)))))))).))))).))))))..))))).))))))..)..)))))    reads      mm         sample
    ......................................................................uuuugagcuuacggggaggaag....................    99         0          seq
    ......................................................................uuuugagcuuacggggaggaaU....................    74         1          seq
    ......................................................................uuuugagcuuacggggaggaagg...................    77         0          seq
```

Supplementary Figure 14. Stem loop structure, read counts and miRNA annotation as determined by miRDeep2 software of novel miRNA candidates.

Provisional ID	:	I_2500
Score total	:	2
Score for star read(s)	:	-1.3
Score for read counts	:	0
Score for mfe	:	1.7
Score for randfold	:	1.6
Score for cons. seed	:	
Total read count	:	85
Mature read count	:	85
Loop read count	:	0
Star read count	:	0

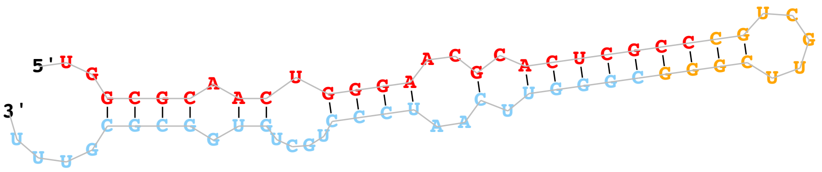

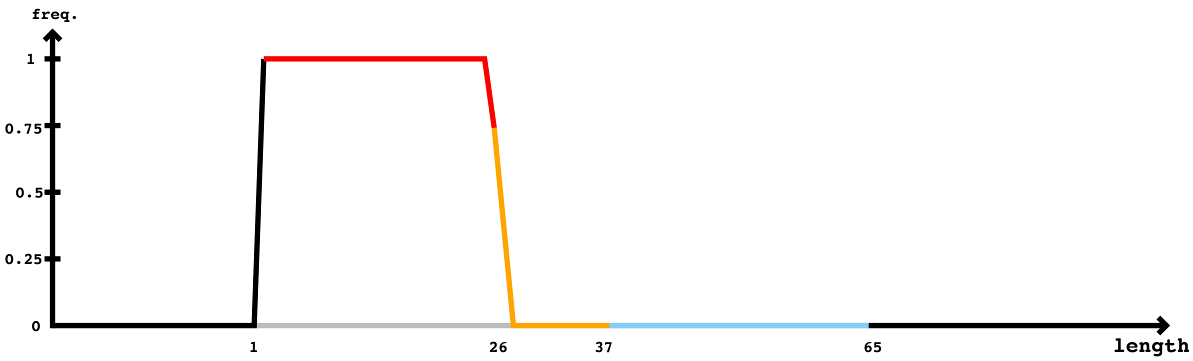

Mature Star

```
5'- ucuauggaaaauugacucgauggcgcaacugggaacgcacucgcccgucguucgggcggguucaaucccugcuguggcgcguuuuccacaugaugauacaaccauagcaugagc  -3'   exp
    ....((((((((..........((((.((.((((..(.(((((((((.....))))))))).)..))))....)).))))))))))))((((((.......)))..))))...   reads    mm      sample
    ....................uggcgcaacugggaacgcacucgc......................................................................   22       0       seq
    ....................uggcgcaacugggaacgcacucgcc.....................................................................   63       0       seq
```

Supplementary Figure 14. Stem loop structure, read counts and miRNA annotation as determined by miRDeep2 software of novel miRNA candidates.

Provisional ID	:	I_5403
Score total	:	2.1
Score for star read(s)	:	-1.3
Score for read counts	:	0
Score for mfe	:	1.8
Score for randfold	:	1.6
Score for cons. seed	:	
Total read count	:	43
Mature read count	:	43
Loop read count	:	0
Star read count	:	0

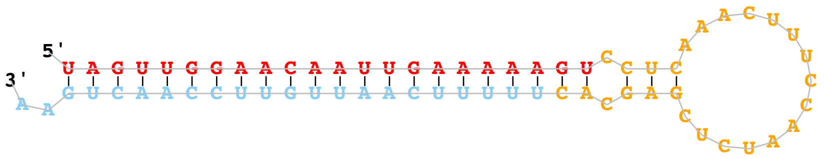

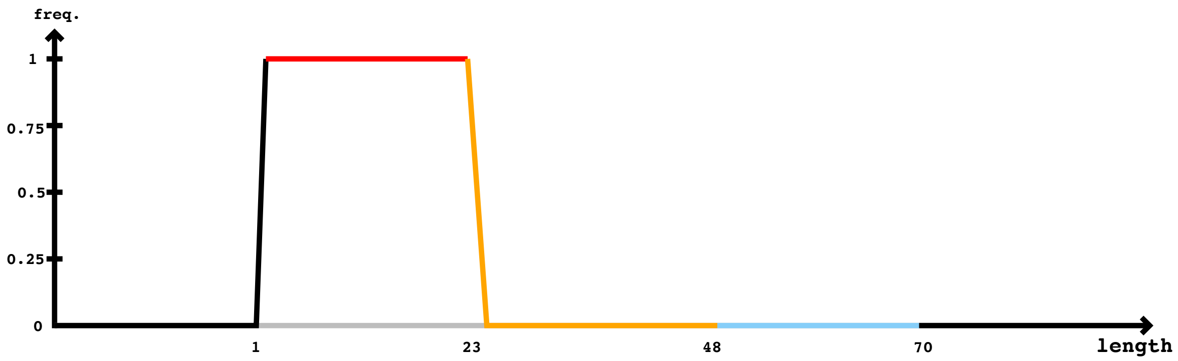

Mature Star

```
5'- ucuagaaaaugagccgaauuuaguuggaacaauugaaaaaguccucaaacuuuccaaucucgagcacuuuuucaauuguuccaacugaaaaaaguuauuuucagagaauuca  -3'   exp
    (((.((((((((......((((((((((((((((((((((((.(((...............))).))))))))))))))))))))))))).....))))))))))).......   reads     mm       sample
    ....................uaguuggaacaauugaaaaagu......................................................................   43        0        seq
```

Supplementary Figure 14. Stem loop structure, read counts and miRNA annotation as determined by miRDeep2 software of novel miRNA candidates.

Provisional ID	:	I_903
Score total	:	2
Score for star read(s)	:	-1.3
Score for read counts	:	0
Score for mfe	:	1.7
Score for randfold	:	1.6
Score for cons. seed	:	
Total read count	:	93
Mature read count	:	68
Loop read count	:	25
Star read count	:	0

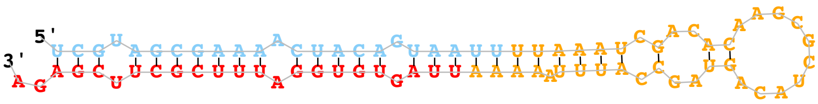

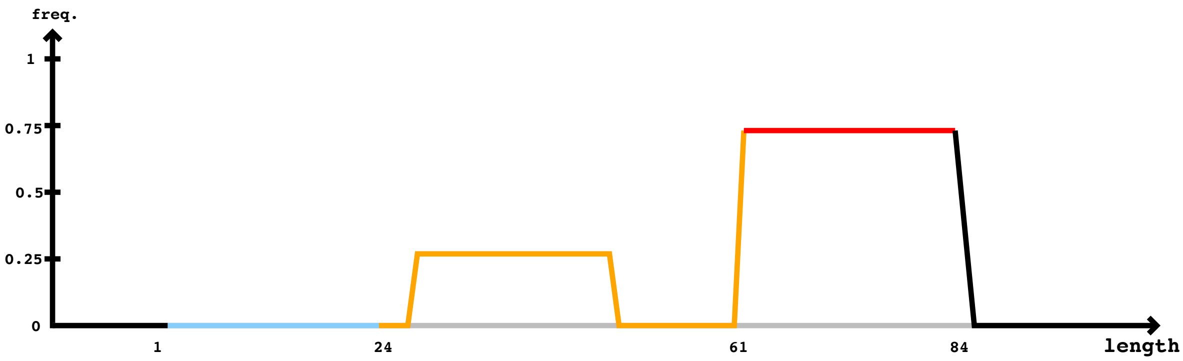

Star Mature

```
5'- gcaaaguaucucguagcgaaaacuacaguaauuuuaaaaucgacacaagcgcuacaguagccauuuaaaaauuaguguggauuucgcuucgagauauuuuguacgucaaauaug   -3'    exp
    (((((((((((((.(((((((.(((((.((((((((((.(..((...........))..).)))).))))))).))))).))))))).))))))))))))))............    reads    mm    sample
    .....................................aaucgacacaagcgcuacagu.......................................................    25       0     seq
    .......................................................................uuaguguggauuucgcuucgaga....................    68       0     seq
```

Supplementary Figure 14. Stem loop structure, read counts and miRNA annotation as determined by miRDeep2 software of novel miRNA candidates.

Provisional ID	:	I_4961
Score total	:	2.7
Score for star read(s)	:	-1.3
Score for read counts	:	0
Score for mfe	:	2.4
Score for randfold	:	1.6
Score for cons. seed	:	
Total read count	:	72
Mature read count	:	61
Loop read count	:	0
Star read count	:	11

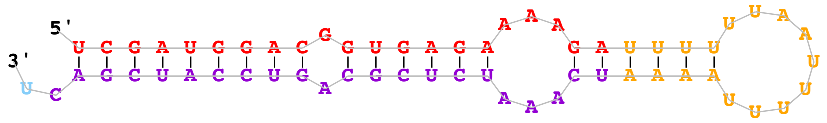

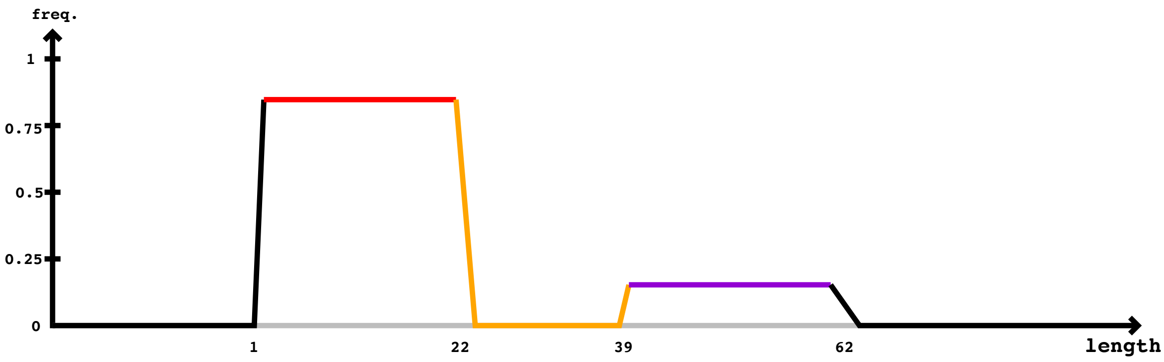

Mature Star

```
5'- ucgauuuagguauuuuaaagucgauggacggugagaaaagauuuuuuaauuuuuuaaaaucaaaucucgcaguccaucgacuuuaaaaauaccuaaaucgaguugaaaaugca  -3'   obs
    ucgauuuagguauuuuaaagucgauggacggugagaaaagauuuuuuaauuuuuuaaaaucaaaucucgcaguccaucgacuuuaaaaauaccuaaaucgaguugaaaaugca         exp
    ((((((((((((((((((((((((((((.((((((...((((((.........))))))...)))))).))))))))))))))))))))))))))))...........   reads    mm      sample
    ....................ucgauggacggugagaaaaga.......................................................................   61       0       seq
    ...........................................................ucaaaucucgcaguccaucgac..............................   11       0       seq
```

Supplementary Figure 14. Stem loop structure, read counts and miRNA annotation as determined by miRDeep2 software of novel miRNA candidates.

Provisional ID	:	I_573
Score total	:	1.4
Score for star read(s)	:	-1.3
Score for read counts	:	0
Score for mfe	:	1.1
Score for randfold	:	1.6
Score for cons. seed	:	
Total read count	:	3593
Mature read count	:	1301
Loop read count	:	0
Star read count	:	2292

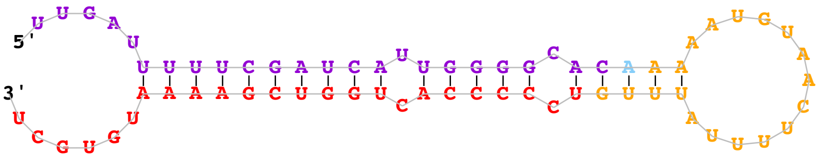

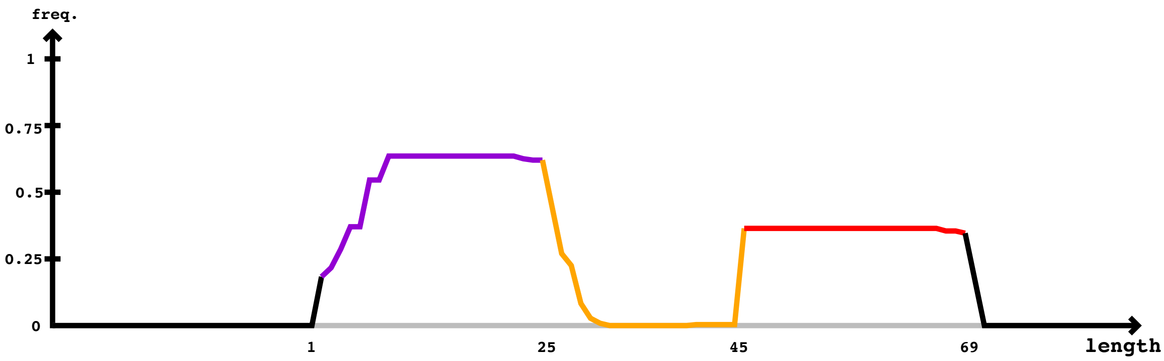


```
                          Star                                          Mature
5'- uuggucuuuuauuagacguugauuaguugauuuuucgaucauuggggcacaaaaauguaacuuuuauuugucccccacuggucgaaaaugugcuuacauuaacgaauaaau   -3'   obs
    uuggucuuuuauuagacguugauuaguugauuuuucgaucauuggggcacaaaaauguaacuuuuauuugucccccacuggucgaaaaugugcuuacauuaacgaauaaau         exp
    ...........(((..(((((((.(((....(((((((((.(((((.(((((.............))))).))))).)))))))))...)))...)))))))..)))..   reads   mm   sample
    ..........................uugauuuuucgaucauugggg................................................................   36      0    seq
    ..........................uugauuuuucgaucauuggggc...............................................................   19      0    seq
    ..........................uugauuuuucgaucauuggggcac.............................................................   592     0    seq
    ..........................uugauuuuucgaucauuggggcacaU...........................................................   13      1    seq
    ...........................ugauuuuucgaucauuggggcac.............................................................   75      0    seq
    ...........................ugauuuuucgaucauuggggcaca............................................................   47      0    seq
    ............................gauuuuucgaucauuggggcac.............................................................   89      0    seq
    ............................gauuuuucgaucauuggggcaca............................................................   162     0    seq
    .............................auuuuucgaucauuggggcac.............................................................   26      0    seq
    .............................auuuuucgaucauuggggcaca............................................................   215     0    seq
    .............................auuuuucgaucauuggggcacaU...........................................................   62      1    seq
    ................................uuucgaucauuggggcacaa...........................................................   86      0    seq
    ................................uuucgaucauuggggcacaaa..........................................................   513     0    seq
    ................................uuucgaucauuggggcacaaaU.........................................................   33      1    seq
    ..................................ucgaucauuggggcaca............................................................   58      0    seq
    ..................................ucgaucauuggggcacaaaa.........................................................   167     0    seq
    ..................................ucgaucauuggggcacaaaaa........................................................   68      0    seq
    ..................................ucgaucauuggggcacaaaaau.......................................................   31      0    seq
    .................................................................auuugucccccacuggucgaaaaugugcu.................   13      0    seq
    ......................................................................ucccccacuggucgaaaaugu....................   35      0    seq
    ......................................................................ucccccacuggucgaaaaugugc..................   30      0    seq
    ......................................................................ucccccacuggucgaaaaugugcu.................   1236    0    seq
```

Supplementary Figure 14. Stem loop structure, read counts and miRNA annotation as determined by miRDeep2 software of novel miRNA candidates.

Provisional ID	:	I_4458
Score total	:	1.6
Score for star read(s)	:	-1.3
Score for read counts	:	0
Score for mfe	:	1.3
Score for randfold	:	1.6
Score for cons. seed	:	
Total read count	:	58
Mature read count	:	58
Loop read count	:	0
Star read count	:	0

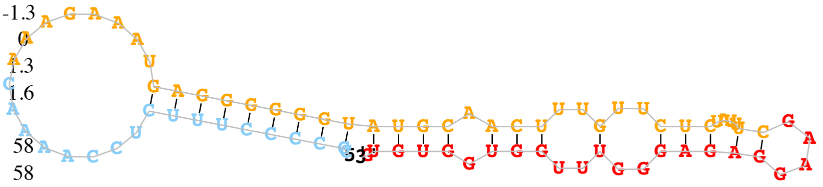

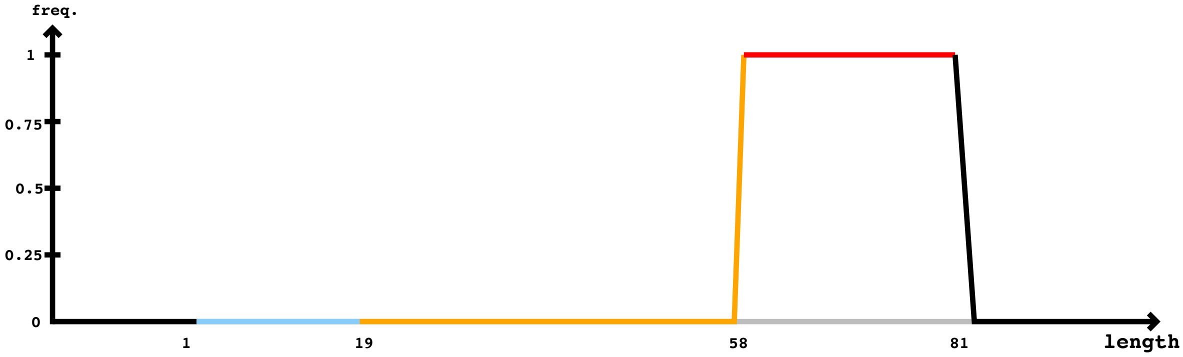

Star Mature

```
5'- gagcaccacaaaacgccccuuucuccaaaacaaagaaaugaggggguaugcaacuuuguucucuauuucgaaggagaggguuugguggugugguggugcugguauuaaucg   -3'    exp
    .((((((((...((((((((((((((........(((((..((((..((........))..)))))))))...)))))))....)).))))).))))).)))...........    reads      mm         sample
    ..................................................................gaaggagaggguuugguggugug...................    58         0          seq
```

Supplementary Figure 14. Stem loop structure, read counts and miRNA annotation as determined by miRDeep2 software of novel miRNA candidates.

Provisional ID	:	II_15249
Score total	:	2
Score for star read(s)	:	-1.3
Score for read counts	:	0
Score for mfe	:	1.7
Score for randfold	:	1.6
Score for cons. seed	:	
Total read count	:	201
Mature read count	:	201
Loop read count	:	0
Star read count	:	0

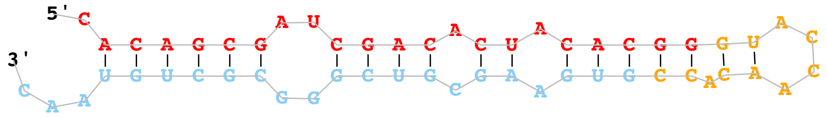

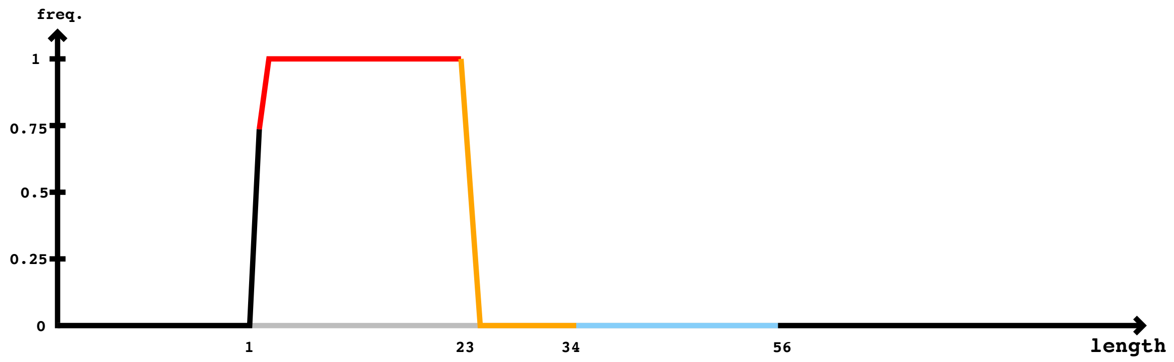

Mature Star

```
5'- ugucggcugcuuugacuuucacagcgaucgacacuacacggguaccaacaccgugaagcgucgggcgcuguaacuucaagaacuuugagcuuaauuuuuaaaguuuaaggu   -3'    exp
    .(((((.....)))))....((((((..((((.((.(((((((....)).))))).)).))))..))))))..........(((((((((((........)))))))))))    reads     mm        sample
    ...................cacagcgaucgacacuacacgg......................................................................    148       0         seq
    ....................acagcgaucgacacuacacgg......................................................................    53        0         seq
```

Supplementary Figure 14. Stem loop structure, read counts and miRNA annotation as determined by miRDeep2 software of novel miRNA candidates.

Provisional ID	:	II_11607
Score total	:	2.8
Score for star read(s)	:	-1.3
Score for read counts	:	0
Score for mfe	:	2.5
Score for randfold	:	1.6
Score for cons. seed	:	
Total read count	:	1371
Mature read count	:	1371
Loop read count	:	0
Star read count	:	0

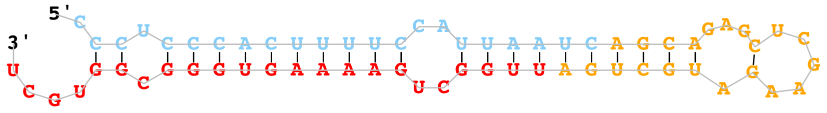

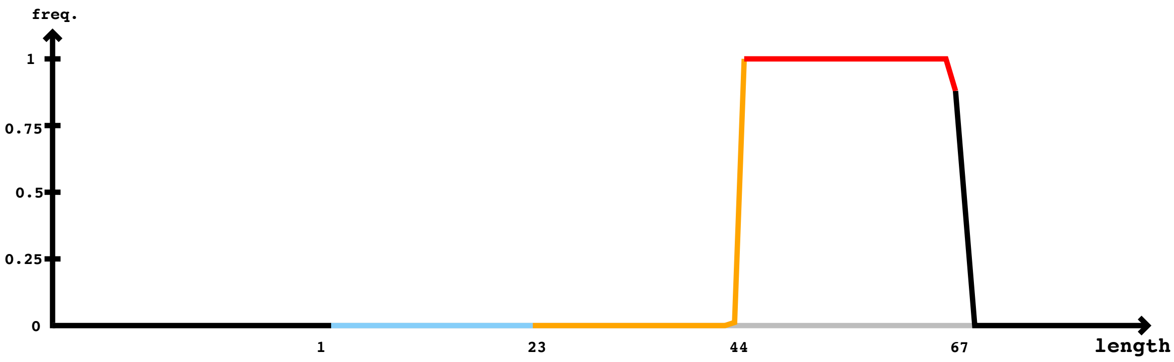


```
                                     Star                                      Mature
5'- aacaucgcccgaccaaucagcgauucgcccucccacuuuuccauuaaaucagcagagcucgaagaugcugauuggcugaaaagugggcggugcuaaucgcugauuggucaguc   -3'    exp
    ..........(((((((((((((((.((((.((((((((((..((((((((((...(.....).))))))))))..)))))))))).)).)).)))))))))))))))....    reads    mm     sample
    ............................................................................auuggcugaaaagugggcggugcu.....................  16       0      seq
    .............................................................................uuggcugaaaagugggcggugc......................  165      0      seq
    .............................................................................uuggcugaaaagugggcggugcu.....................  1126     0      seq
    .............................................................................uuggcugaaaagugggcggugcuU....................  64       1      seq
```

Supplementary Figure 14. Stem loop structure, read counts and miRNA annotation as determined by miRDeep2 software of novel miRNA candidates.

Provisional ID	:	II_14714
Score total	:	2.1
Score for star read(s)	:	-1.3
Score for read counts	:	0
Score for mfe	:	1.8
Score for randfold	:	1.6
Score for cons. seed	:	
Total read count	:	838
Mature read count	:	838
Loop read count	:	0
Star read count	:	0

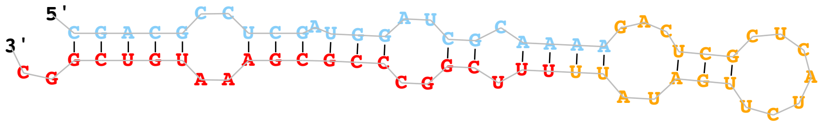

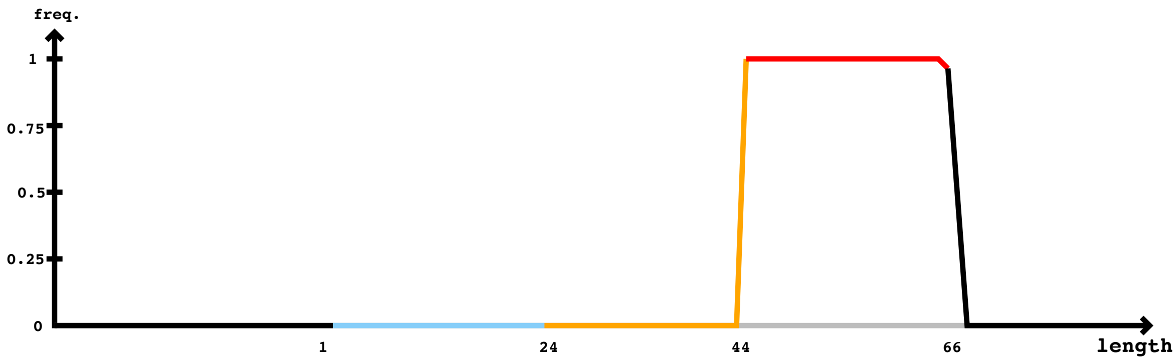


```
                                         Star                                         Mature
5'- uauuuuuucuccgagcuuccuugcagccgacgccucgauggaucgcaaaagacucgcucaucuugauauuuuucggcccgcgaaaugucggcugccaggcaagcgugcaaau   -3'    exp
    ..............((((((.((((((((((..(((.(((..((.((((...(((.......)))..)))).))..))))))..))))))))))).))).)))).......    reads    mm    sample
    ......................................................................uuucggcccgcgaaaugucgg....................    30       0     seq
    ......................................................................uuucggcccgcgaaaugucggc...................    696      0     seq
    ......................................................................uuucggcccgcgaGaugucggc...................    112      1     seq
```

Supplementary Figure 14. Stem loop structure, read counts and miRNA annotation as determined by miRDeep2 software of novel miRNA candidates.

Provisional ID	:	II_12610
Score total	:	1.3
Score for star read(s)	:	-1.3
Score for read counts	:	0
Score for mfe	:	1
Score for randfold	:	1.6
Score for cons. seed	:	
Total read count	:	24
Mature read count	:	24
Loop read count	:	0
Star read count	:	0

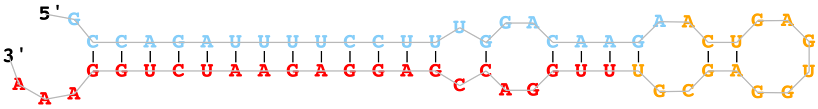

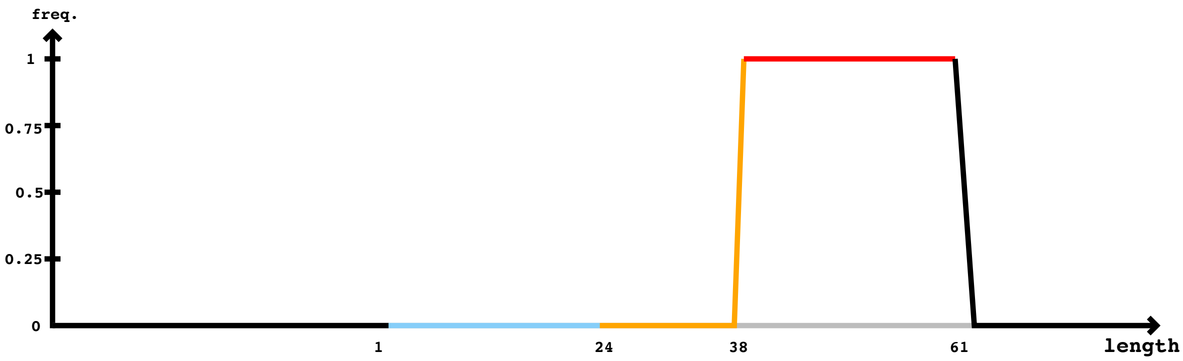

Star Mature

```
5'- cgacggaccaaaugcgauagaauggucacauuagccagauuuuccuuuggacaagaacugaguggagcguuuggaccgaggagaaucuggaaauauaucaaugcuaaacuccc   -3'   exp
    ....(((...((((.(((......))).))))..(((((((((((((.(..((((..((......))..))))..).)))))))))))))...................))).    reads   mm   sample
    ......................................................................uuggaccgaggagaaucuggaaa....................    24      0    seq
```

Supplementary Figure 14. Stem loop structure, read counts and miRNA annotation as determined by miRDeep2 software of novel miRNA candidates.

Provisional ID	:	II_8868
Score total	:	1.8
Score for star read(s)	:	-1.3
Score for read counts	:	0
Score for mfe	:	1.5
Score for randfold	:	1.6
Score for cons. seed	:	
Total read count	:	22
Mature read count	:	22
Loop read count	:	0
Star read count	:	0

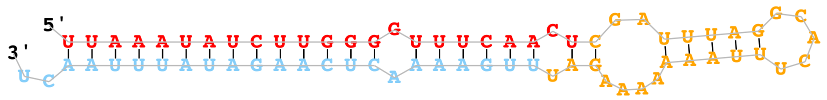

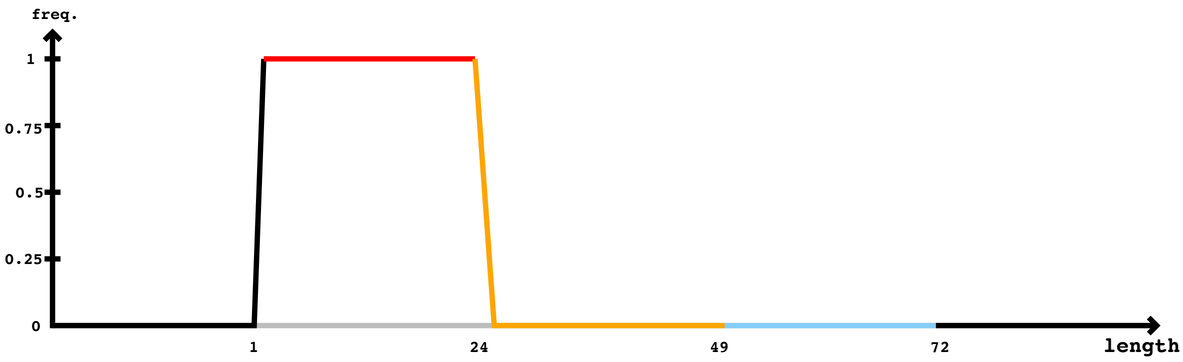


```
                        Mature                                          Star
5'- ucgaauuuuggguaaacaaguuaaauaucuuggggguuucaacucgauuuaggcacuuuaaaaaaaagauuugaaaacucaagauauuuaacuuguaaaaccaaauuagacggug   -3'   exp
    ((....(((((....(((((((((((((((((((.((((((.((..(((((.....)))))....)).)))))).)))))))))))))))))))....)))))...)).....   reads   mm   sample
    ....................uuaaauaucuuggggguuucaacu......................................................................   22      0    seq
```

Supplementary Figure 14. Stem loop structure, read counts and miRNA annotation as determined by miRDeep2 software of novel miRNA candidates.

Provisional ID	:	III_22170
Score total	:	2.6
Score for star read(s)	:	-1.3
Score for read counts	:	0
Score for mfe	:	2.3
Score for randfold	:	1.6
Score for cons. seed	:	
Total read count	:	549
Mature read count	:	549
Loop read count	:	0
Star read count	:	0

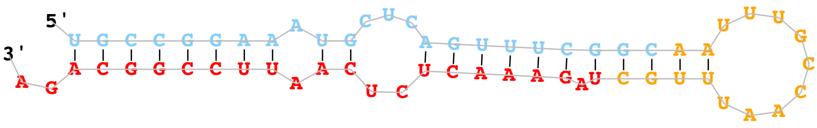

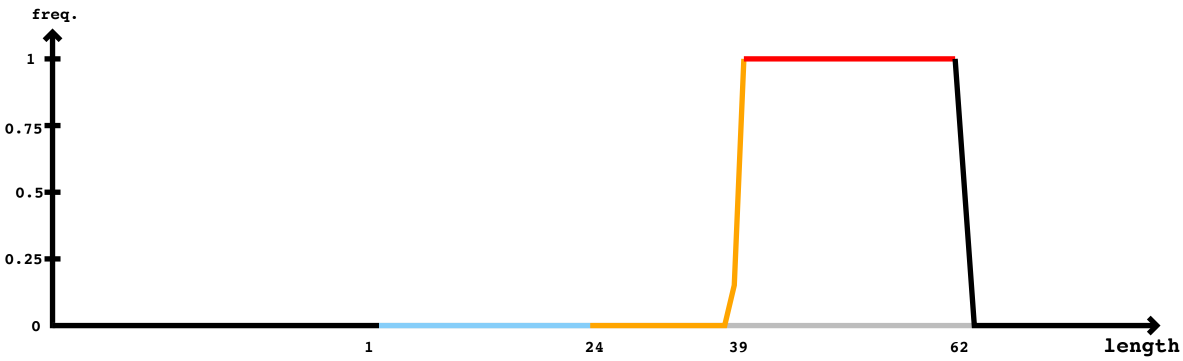


```
                                      Star                                 Mature
5'- ccggaagcuuucauuauggcaaauugccgauuugccggaaaugcucaguuucggcaauuugccaauuugcuagaaacucucaauuccggcagauuggcgauuugccgauuucc   -3'   exp
    .(....).........((((((((((((((((((((((((.((...(((((((((((.........))))).)))))))..)).)))))))))))))))))))))))))......   reads   mm   sample
    .....................................................................cuagaaacucucaauuccggcaga....................   83      0    seq
    ......................................................................uagaaacucucaauuccggcaga....................   466     0    seq
```

Supplementary Figure 14. Stem loop structure, read counts and miRNA annotation as determined by miRDeep2 software of novel miRNA candidates.

Provisional ID	:	III_21441
Score total	:	2.1
Score for star read(s)	:	-1.3
Score for read counts	:	0
Score for mfe	:	1.8
Score for randfold	:	1.6
Score for cons. seed	:	
Total read count	:	50
Mature read count	:	50
Loop read count	:	0
Star read count	:	0

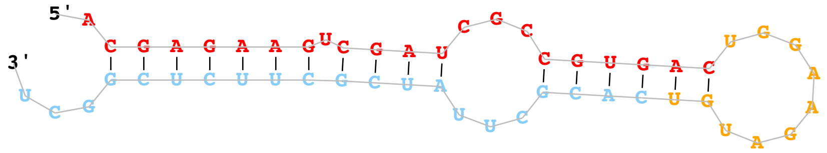

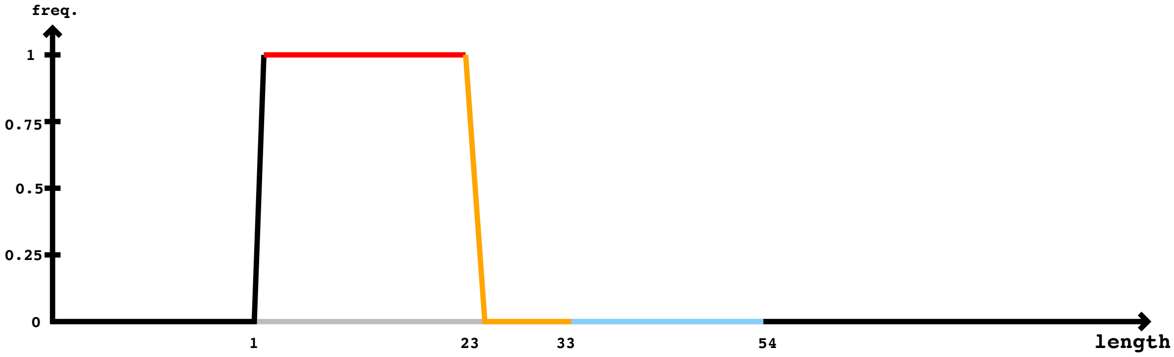

Mature Star

```
5'- acgacaccaaucuuuggguaacgagaagucgaucgccgugacuggaagaugucacgcuuaucgcuucucggcuugacggcagccguagugcuacugucgucguucugucaag   -3'    exp
    ..((((((.....)))....((((((.((((...((((((........))))))...)))))))))))....((((((((..(.....)..))))))))....))))...    reads    mm    sample
    ....................acgagaagucgaucgccgugac......................................................................    50       0     seq
```

Supplementary Figure 14. Stem loop structure, read counts and miRNA annotation as determined by miRDeep2 software of novel miRNA candidates.

Provisional ID	:	III_17499
Score total	:	2.8
Score for star read(s)	:	-1.3
Score for read counts	:	0
Score for mfe	:	2.5
Score for randfold	:	1.6
Score for cons. seed	:	
Total read count	:	172
Mature read count	:	172
Loop read count	:	0
Star read count	:	0

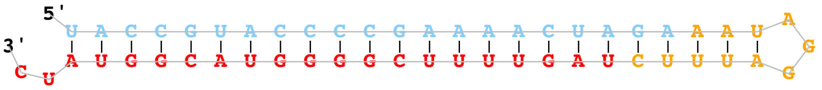

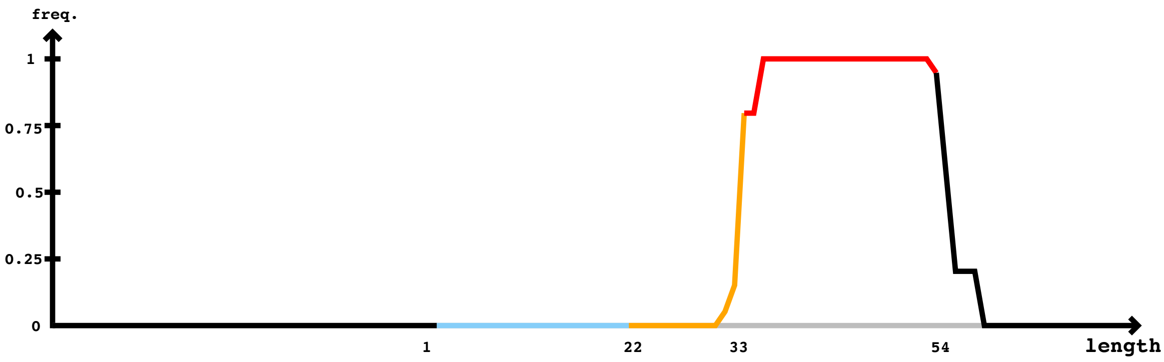

Star Mature

```
5'- guaagugauguuuacucagguacucauauucuauuagauaccguaccccgaaaacuagaaauaggauuucuaguuuucgggguacgguaucuaauagaaugucuuccagaa   -3'    exp
    (((..(((.(....))))..)))..(((((((((((((((((((((((((((((((((((((...)))))))))))))))))))))))))))))))))))))).........    reads      mm        sample
    ....................................................................ucuaguuuucgggguacgguaC.....................    9          1         seq
    .....................................................................cuaguuuucgggguacgguaucu...................    17         0         seq
    ......................................................................uaguuuucgggguacgguauc....................    49         0         seq
    ......................................................................uaguuuucgggguacgguauA....................    16         1         seq
    ......................................................................uaguuuucgggguacgguaucu...................    46         0         seq
    .........................................................................guuuucgggguacgguaucuaau................    35         0         seq
```

Supplementary Figure 14. Stem loop structure, read counts and miRNA annotation as determined by miRDeep2 software of novel miRNA candidates.

Provisional ID	:	IV_27441
Score total	:	2.7
Score for star read(s)	:	-1.3
Score for read counts	:	0
Score for mfe	:	2.4
Score for randfold	:	1.6
Score for cons. seed	:	
Total read count	:	27
Mature read count	:	27
Loop read count	:	0
Star read count	:	0

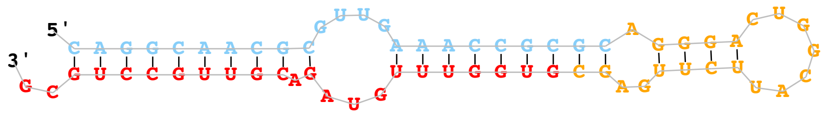

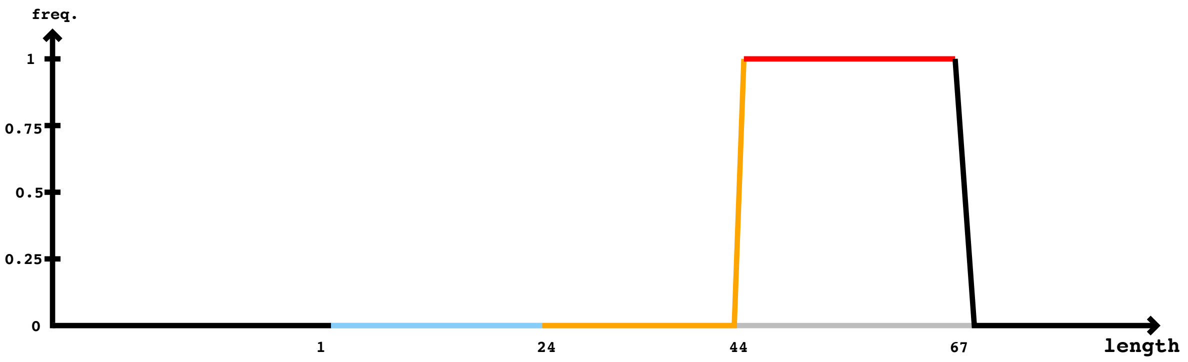


```
                                   Star                       Mature
5'- gcgaacugccauugcugcaagaccacgcaggcaacgcguugaaaccgcgcagggacuggcauucuugagcgugguuuguagacguugccugcgugguuuggagcggcgccugc   -3'   exp
    (((...((((...(((.((.((((((((((((((((((....((((((((.((((.......))))..)))))))))...).))))))))))))))))))).)))))))..)))    reads      mm        sample
    ......................................................................gugguuuguagacguugccugcg....................    27         0         seq
```

Supplementary Figure 14. Stem loop structure, read counts and miRNA annotation as determined by miRDeep2 software of novel miRNA candidates.

Provisional ID	:	IV_31230
Score total	:	2.5
Score for star read(s)	:	-1.3
Score for read counts	:	0
Score for mfe	:	2.2
Score for randfold	:	1.6
Score for cons. seed	:	
Total read count	:	48
Mature read count	:	48
Loop read count	:	0
Star read count	:	0

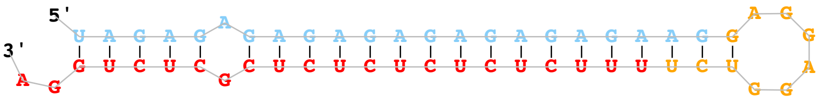

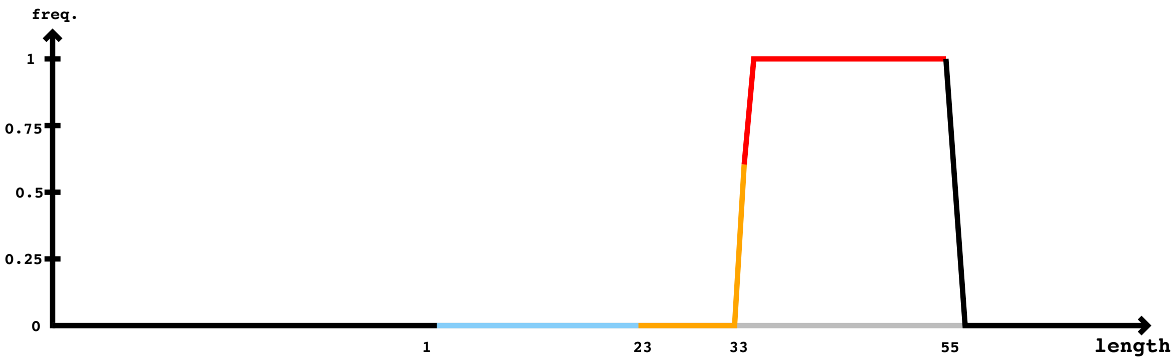

Star Mature

```
5'- gcgacgagggcggagucauuucucauuugggcaggugguagagagagagagagagagaaggaggaggucuuuucucucucucucgcucuggauuagacugccauggugccua  -3'   exp
    .......((((.(((......))).....(((((.((((((((.(((((((((((((((((......))))))))))))))))).))))..))))..))))).....)))).   reads   mm   sample
    ......................................................................uuucucucucucucgcucugga....................   29      0    seq
    .......................................................................uucucucucucucgcucuggau...................   19      0    seq
```

Supplementary Figure 14. Stem loop structure, read counts and miRNA annotation as determined by miRDeep2 software of novel miRNA candidates.

Provisional ID	:	IV_31091
Score total	:	1.7
Score for star read(s)	:	-1.3
Score for read counts	:	0
Score for mfe	:	1.4
Score for randfold	:	1.6
Score for cons. seed	:	
Total read count	:	1707
Mature read count	:	1707
Loop read count	:	0
Star read count	:	0

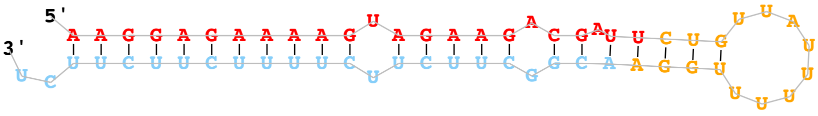

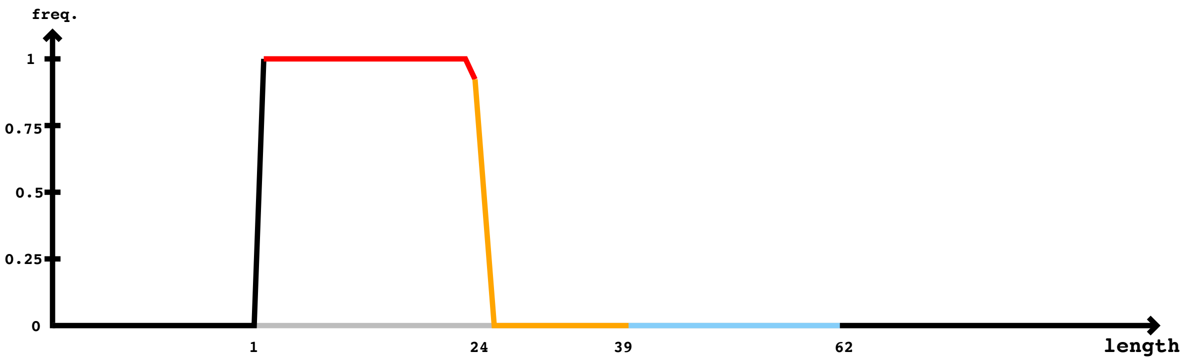

Mature Star

```
5'- uucugagcauugcaaaaaagaaggagaaaaguagaagacgauucuguuauuuuuuggaacggcuucuucuuuucuucuucuuguugagcuuuuucucacuuauuugcuucugu   -3'    exp
    ......(((..(((((.((((((((((((((.(((((.((.(((((........))))))).))))).))))))))))))))..((((......))))....)))))...)))    reads     mm        sample
    ....................aaggagaaaaguagaagacgau.......................................................................    119       0         seq
    ....................aaggagaaaaguagaagacgaC.......................................................................    12        1         seq
    ....................aaggagaaaaguagaagacgauu......................................................................    1558      0         seq
    ....................aaggagaaaaguagaagacgauuc.....................................................................    18        0         seq
```

Supplementary Figure 14. Stem loop structure, read counts and miRNA annotation as determined by miRDeep2 software of novel miRNA candidates.

Provisional ID	:	IV_29322
Score total	:	2.1
Score for star read(s)	:	-1.3
Score for read counts	:	0
Score for mfe	:	1.8
Score for randfold	:	1.6
Score for cons. seed	:	
Total read count	:	97
Mature read count	:	97
Loop read count	:	0
Star read count	:	0

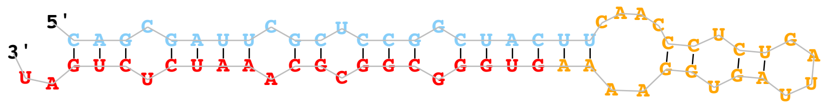

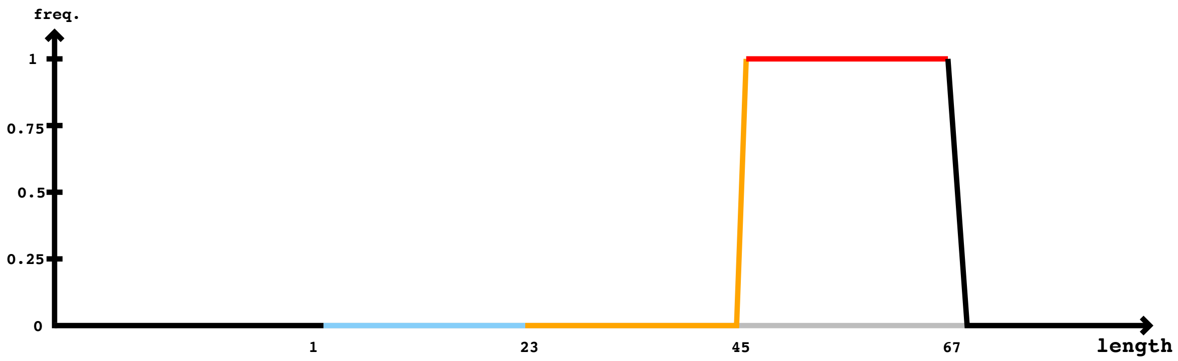

Star Mature

```
5'- cuccgaaagugagagcugggaccaaucagcgauucgcuccggcuacuucaacccucugauuaguggaaaagugggcggcgcaaaucucugauuuguugaauaucucacauug   -3'    exp
    ........((((((.....(((.((((((.((((.((.(((.((((((....((.((....)).))..)))))).))).)).)))).)))))).))).....))))))....   reads      mm         sample
    ......................................................................gugggcggcgcaaaucucugau....................   56         0          seq
    ......................................................................gugggcggcgcaaaucucugauu...................   41         0          seq
```

Supplementary Figure 14. Stem loop structure, read counts and miRNA annotation as determined by miRDeep2 software of novel miRNA candidates.

Provisional ID	:	IV_23520
Score total	:	1.8
Score for star read(s)	:	-1.3
Score for read counts	:	0
Score for mfe	:	1.5
Score for randfold	:	1.6
Score for cons. seed	:	
Total read count	:	96
Mature read count	:	96
Loop read count	:	0
Star read count	:	0

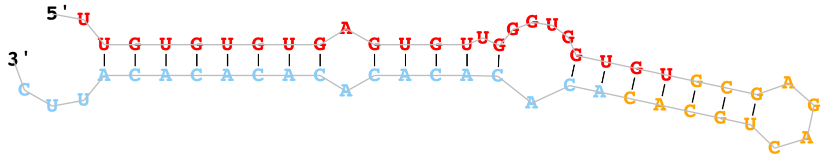

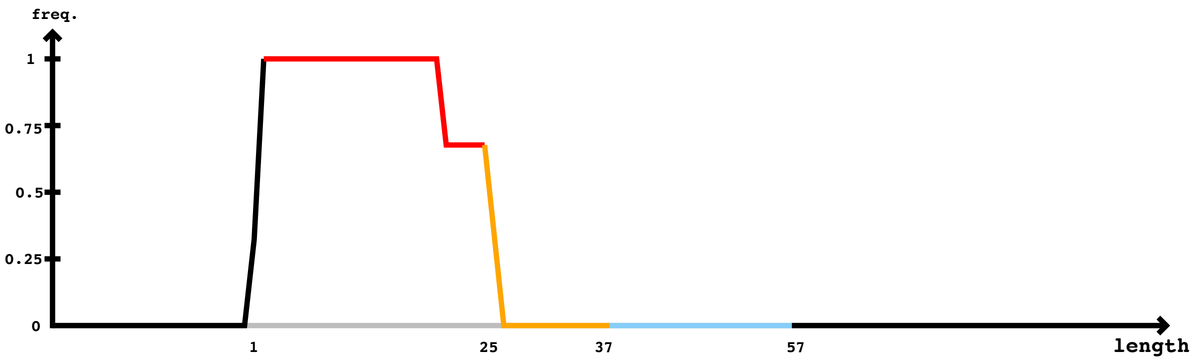

Mature Star

```
5'- gcuucuucucuuuucucuuauugugugugaguguuggguggugugcgagacugcacacacacacacacacacauucucauagugagcucucacgucgucuggagcccugcggca   -3'   exp
    (((.(..((((...(......((((((((.((((.(....(((((((....))))))).))))).))))))))........((((....))))...)...))))....).))).    reads   mm   sample
    ...................auugugugugaguguugggu...........................................................................    31      0    seq
    ....................uugugugugaguguuggguggugu......................................................................    65      0    seq
```

Supplementary Figure 14. Stem loop structure, read counts and miRNA annotation as determined by miRDeep2 software of novel miRNA candidates.

Provisional ID	:	IV_28960
Score total	:	2.3
Score for star read(s)	:	-1.3
Score for read counts	:	0
Score for mfe	:	2
Score for randfold	:	1.6
Score for cons. seed	:	
Total read count	:	134
Mature read count	:	68
Loop read count	:	0
Star read count	:	66

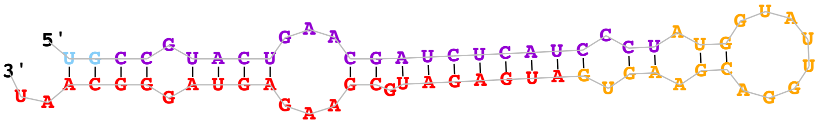

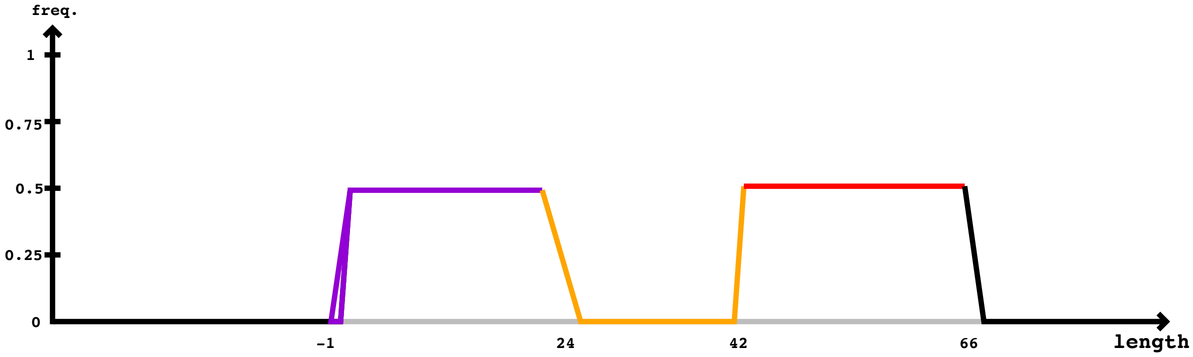

Star Mature

```
5'- aucagaggucacucuagcucaguccauugccguacugaacgaucucaucccuaugguauuggacgaagugaugagaugcgaagaguagggcaauggauaugacgagcaucgggg  -3'   obs
    aucagaggucacucuagcucaguccauugccguacugaacgaucucaucccuaugguauuggacgaagugaugagaugcgaagaguagggcaauggauaugacgagcaucgggg        exp
    ...((((....)))).((((.(((((((((.((((...(((((((((.((.((........)).)).)))))))).))...)))).))))))))).....))))......   reads   mm   sample
    ............................ccguacugaacgaucucaucccu.............................................................   66      0    seq
    ..................................................................augagaugcgaagaguagggcaau...................   68      0    seq
```

Supplementary Figure 14. Stem loop structure, read counts and miRNA annotation as determined by miRDeep2 software of novel miRNA candidates.

Provisional ID	:	IV_23346
Score total	:	1.7
Score for star read(s)	:	-1.3
Score for read counts	:	0
Score for mfe	:	1.4
Score for randfold	:	1.6
Score for cons. seed	:	
Total read count	:	155
Mature read count	:	155
Loop read count	:	0
Star read count	:	0

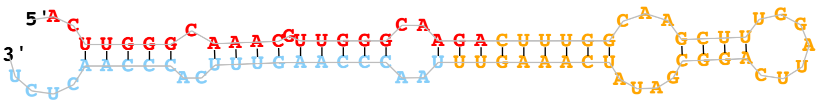

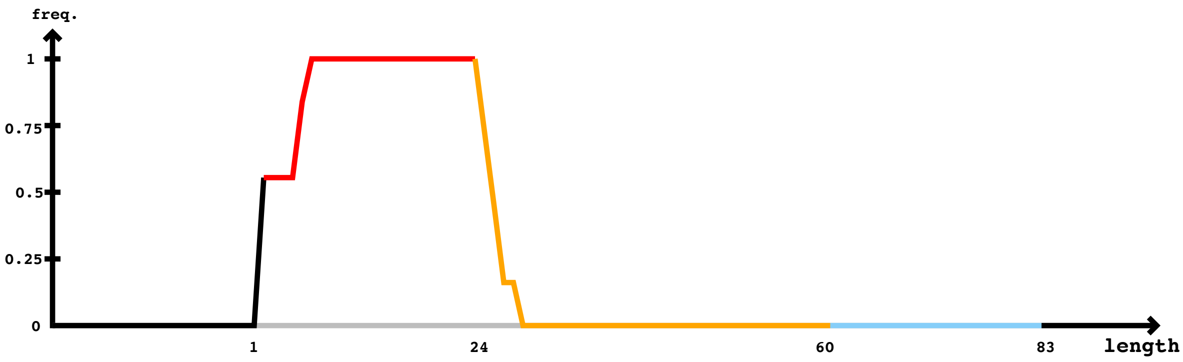


```
                    Mature                                                  Star
5'- guugacuaaacuuuggcuaaacuugggcaaacguugggcaagacuuuggcaagcuuuggauucaggcgauaucaaaguuuaacccaaguuucacccaacucuugccaaacguu   -3'    exp
    ........(((((((((.....(((((.((((.(((((..(((((((((...((((.......))))....)))))))))..)))))))))..))))).....)))))).)))   reads     mm      sample
    ....................acuugggcaaacguugggcaaga..........................................................................   86        0       seq
    ........................gggcaaaAguugggcaagacu........................................................................   44        1       seq
    .........................ggcaaaAguugggcaagacuuu......................................................................   25        1       seq
```

Supplementary Figure 14. Stem loop structure, read counts and miRNA annotation as determined by miRDeep2 software of novel miRNA candidates.

Provisional ID	:	IV_22999
Score total	:	2.2
Score for star read(s)	:	-1.3
Score for read counts	:	0
Score for mfe	:	1.9
Score for randfold	:	1.6
Score for cons. seed	:	
Total read count	:	86
Mature read count	:	86
Loop read count	:	0
Star read count	:	0

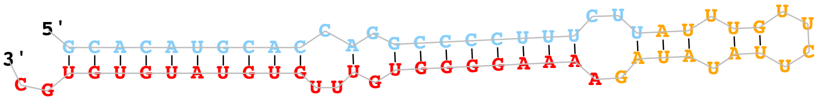

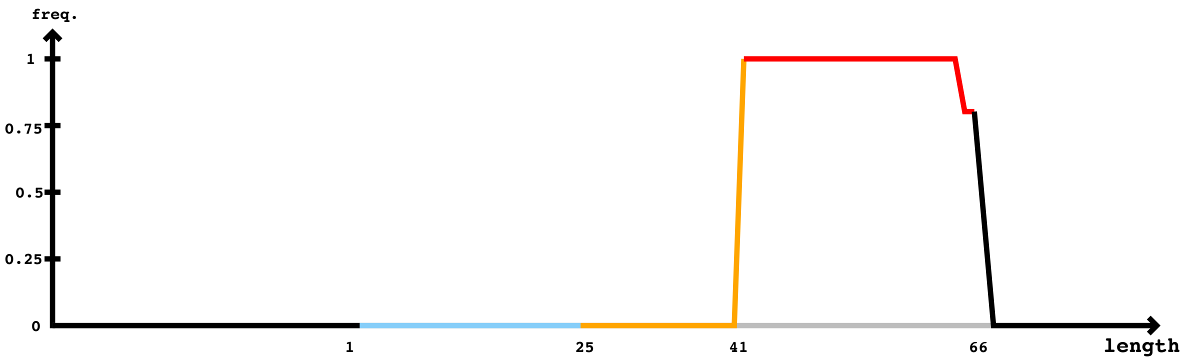

Star Mature

```
5'- gacggagcagaagaagacguugagcacagagcacaugcaccaggccccuuucuuauuuguucuuauauagaaaagggguguuuguguaugugugcuccgggauuccgggagga   -3'    exp
    ..((((((..........((.....))...((((((((((.(.((((((((..(((.((....)).)))..)))))))).)..))))))))))))))))....(((....)))   reads      mm        sample
    ......................................................................aaaagggguguuuguguaugugu....................   17         0         seq
    ......................................................................aaaagggguguuuguguaugugugc..................   69         0         seq
```

Supplementary Figure 14. Stem loop structure, read counts and miRNA annotation as determined by miRDeep2 software of novel miRNA candidates.

Provisional ID	:	V_36591
Score total	:	1.7
Score for star read(s)	:	-1.3
Score for read counts	:	0
Score for mfe	:	1.4
Score for randfold	:	1.6
Score for cons. seed	:	
Total read count	:	82
Mature read count	:	82
Loop read count	:	0
Star read count	:	0

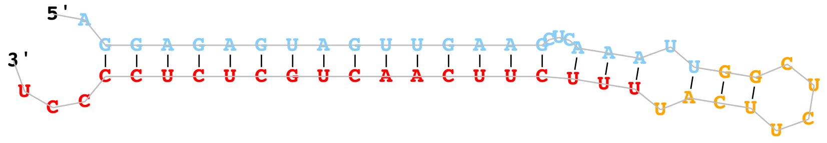

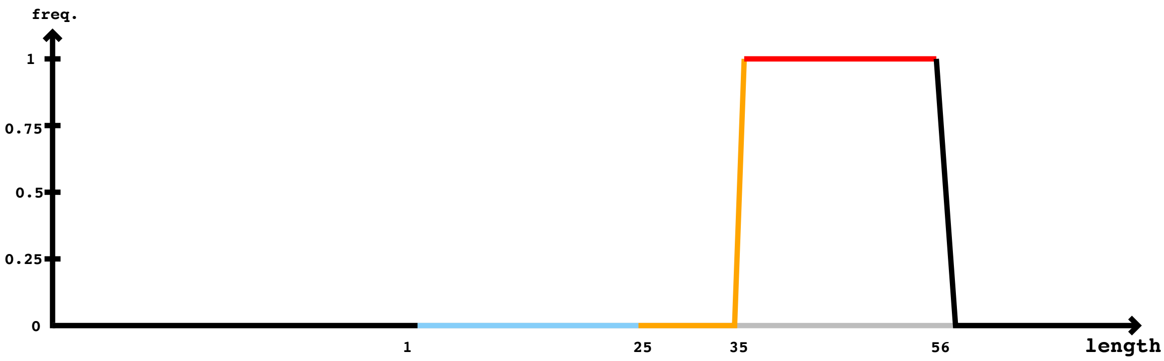

Star Mature

```
5'- ucucucucuuaauuucucccucccacauugaagauuaggagaguaguugaagcucaaauuggcucuucauuuucuucaacugcucuccccuuuuuacgggguggaaucgag  -3'   exp
    .......((..(((((.((((........((((....((((((((((((((...(((.(((....))).)))))))))))))))))).))))....)))).)))))..))   reads   mm   sample
    ......................................................................uuucuucaacugcucuccccu....................   82      0    seq
```

Supplementary Figure 14. Stem loop structure, read counts and miRNA annotation as determined by miRDeep2 software of novel miRNA candidates.

Provisional ID	:	V_39446
Score total	:	1.3
Score for star read(s)	:	-1.3
Score for read counts	:	0
Score for mfe	:	1
Score for randfold	:	1.6
Score for cons. seed	:	
Total read count	:	348
Mature read count	:	348
Loop read count	:	0
Star read count	:	0

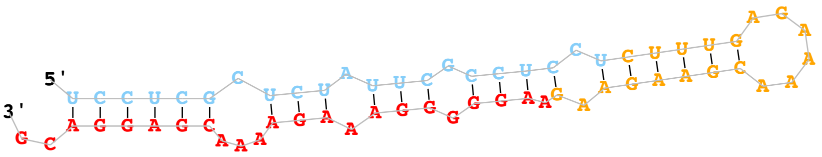

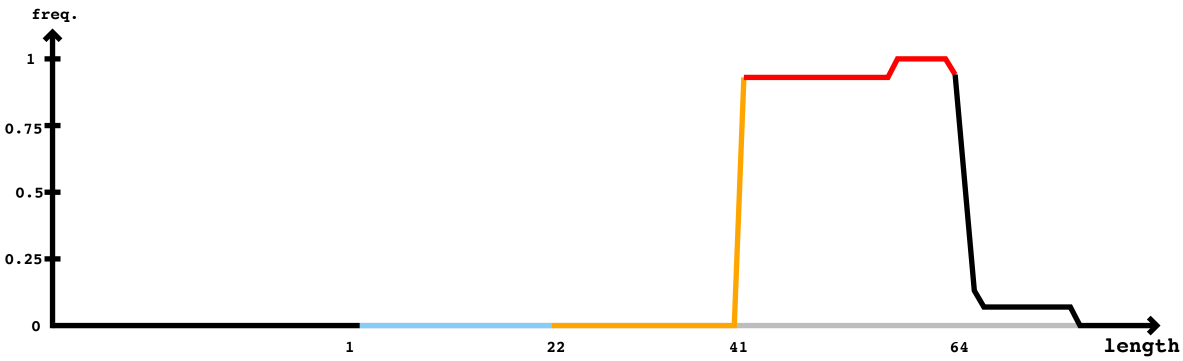


```
                                   Star                                   Mature
5'- auaguccucagcagucgucguuucgcuuauuccucgcucuauucgccuccucuuugagaaaacgaagaagaagggggaaagaaaacgaggacgaaaggagaagacaaacgucu   -3'    exp
    .............(((.((.(((((.....((((((.(((.(((.((((.((((((......)))))).).))).))).)))...))))))))))).))...)))........   reads   mm   sample
    ......................................................................aagggggaaagaaaacgaggac.....................   22      0    seq
    ......................................................................aagggggaaagaaaacgaggacg....................   192     0    seq
    ......................................................................aagggggaaagaaaacgaggacga...................   111     0    seq
    ......................................................................aagggggaaagaaaacgaggacgaa..................   23      0    seq
    ......................................................................................gaggacgaaaggagaagac........   26      0    seq
```

Supplementary Figure 14. Stem loop structure, read counts and miRNA annotation as determined by miRDeep2 software of novel miRNA candidates.

Provisional ID	:	V_38830
Score total	:	1.9
Score for star read(s)	:	-1.3
Score for read counts	:	0
Score for mfe	:	1.6
Score for randfold	:	1.6
Score for cons. seed	:	
Total read count	:	263
Mature read count	:	263
Loop read count	:	0
Star read count	:	0

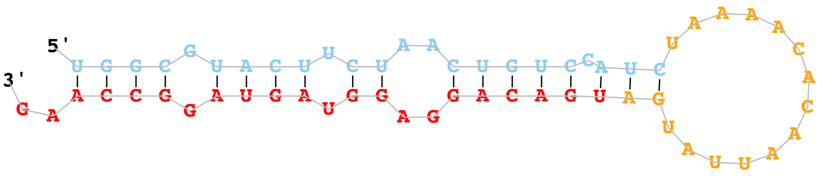

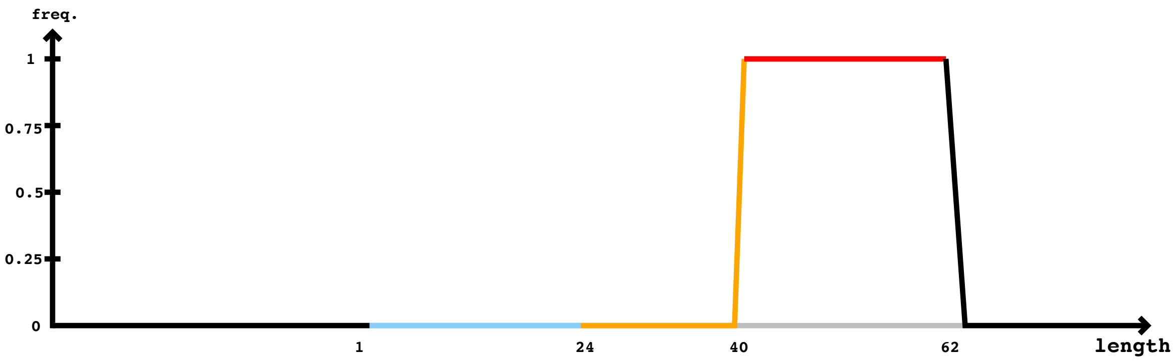

Star Mature

```
5'- guaauacuuuuuuaaagcaucaacuacuacuuggcguacuucuaacuguccaucuaaaacacaauuaugaugacaggagguaguaggccaaguagucugaagcaaaaguuuc  -3'   exp
    .....((((((.....((.(((...(((((((((.((((.((..(((((.(((...............))))))))..)).)))).))))))))).))).))))))))...   reads   mm   sample
    .................................................................ugacaggagguaguaggccaag...................   263     0    seq
```

Supplementary Figure 14. Stem loop structure, read counts and miRNA annotation as determined by miRDeep2 software of novel miRNA candidates.

Provisional ID	:	V_37758
Score total	:	3.1
Score for star read(s)	:	-1.3
Score for read counts	:	0
Score for mfe	:	2.8
Score for randfold	:	1.6
Score for cons. seed	:	
Total read count	:	79
Mature read count	:	79
Loop read count	:	0
Star read count	:	0

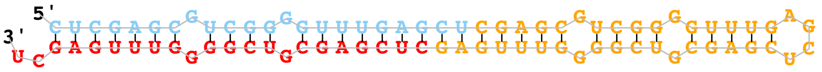

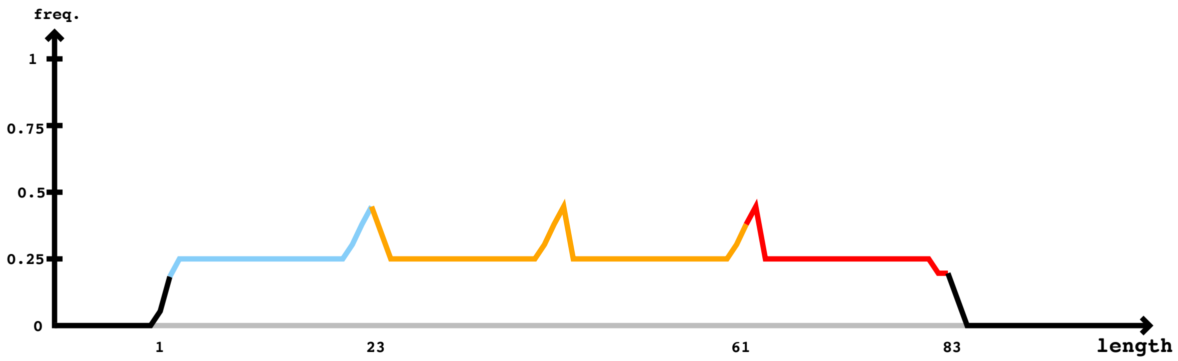


```
                Star                                                      Mature
5'- ggggcuugagcucgagcgucgggguuugagcucgagcgucgggguuugagcucgagcgucgggguuugagcucgagcgucgggguuugagcucgagcgucggggcgcuugcg   -3'    exp
    ((.(((((((((((((.((((.(((((((((((((((.((((.(((((....))))).)))).))))))))))))))).)))).))))))))))))).))...((....)).    reads   mm   sample
    .........gcucgagcgucgggguuugag..................................................................................    68      0    seq
    ..........cucgagcgucgggguuugagcu................................................................................    164     0    seq
    ...........ucgagcgucgggguuugagcu................................................................................    84      0    seq
    .............................gcucgagcgucgggguuugag..............................................................    68      0    seq
    ..............................cucgagcgucgggguuugagcu............................................................    164     0    seq
    ...............................ucgagcgucgggguuugagcu............................................................    84      0    seq
    .................................................gcucgagcgucgggguuugag..........................................    68      0    seq
    ..................................................cucgagcgucgggguuugagcu........................................    164     0    seq
    ...................................................ucgagcgucgggguuugagcu........................................    84      0    seq
    .....................................................................gcucgagcgucgggguuugag......................    68      0    seq
    ......................................................................cucgagcgucgggguuugagcu....................    164     0    seq
    .......................................................................ucgagcgucgggguuugagcu....................    84      0    seq
```

Supplementary Figure 14. Stem loop structure, read counts and miRNA annotation as determined by miRDeep2 software of novel miRNA candidates.

Provisional ID	:	V_37551
Score total	:	1.9
Score for star read(s)	:	-1.3
Score for read counts	:	0
Score for mfe	:	1.6
Score for randfold	:	1.6
Score for cons. seed	:	
Total read count	:	45
Mature read count	:	45
Loop read count	:	0
Star read count	:	0

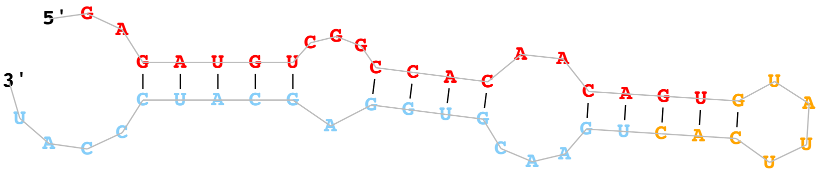

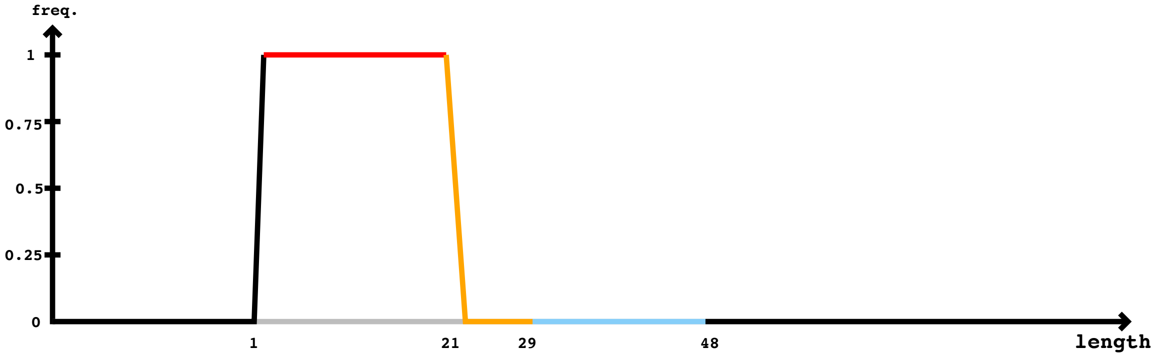

Mature Star

```
5'- cuggauucacuuugcacagugagaugucggccacaacaguguauucacugaacguggagcaucccauaaaauggaacaacugcuuuccguugcaugugaaucucgaugaa  -3'   exp
    (.((((((((..(((...(((.(((((...((((..(((((....)))))...)))).))))).)))..((((((((........))))))))).)))))))).).....   reads   mm   sample
    ...................gagaugucggccacaacagu.....................................................................   45      0    seq
```

Supplementary Figure 14. Stem loop structure, read counts and miRNA annotation as determined by miRDeep2 software of novel miRNA candidates.

Provisional ID	:	X_43454
Score total	:	1.4
Score for star read(s)	:	-1.3
Score for read counts	:	0
Score for mfe	:	1.1
Score for randfold	:	1.6
Score for cons. seed	:	
Total read count	:	84
Mature read count	:	84
Loop read count	:	0
Star read count	:	0

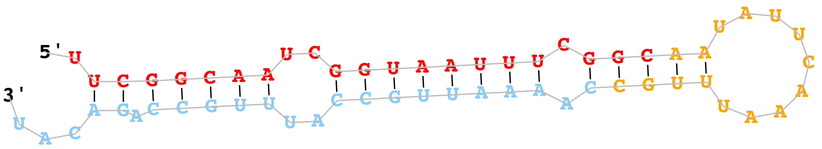

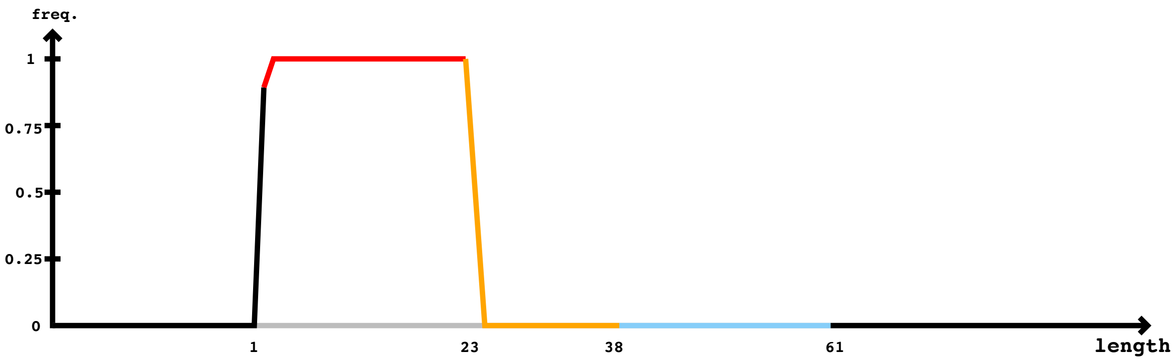

Mature Star

```
5'- acuugccgguuuugggaaauuucggcaaucgguaauuucggcaauauucaaauuugccaaaauugccauuugccagacauuuucggcaauuuucaaacccuauauuucggaa  -3'   exp
    ..((((((.......(((((.(((((((..((((((((.(((((.........))))).))))))))..))))).)).))))))))))).........((........))..    reads   mm   sample
    ....................uucggcaaucgguaauuucggc......................................................................    75      0    seq
    .....................ucggcaaucgguaauuucggc......................................................................    9       0    seq
```

Supplementary Figure 14. Stem loop structure, read counts and miRNA annotation as determined by miRDeep2 software of novel miRNA candidates.

Provisional ID	:	X_43200
Score total	:	2.6
Score for star read(s)	:	-1.3
Score for read counts	:	0
Score for mfe	:	2.3
Score for randfold	:	1.6
Score for cons. seed	:	
Total read count	:	71
Mature read count	:	71
Loop read count	:	0
Star read count	:	0

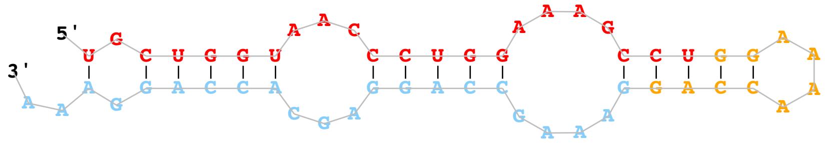

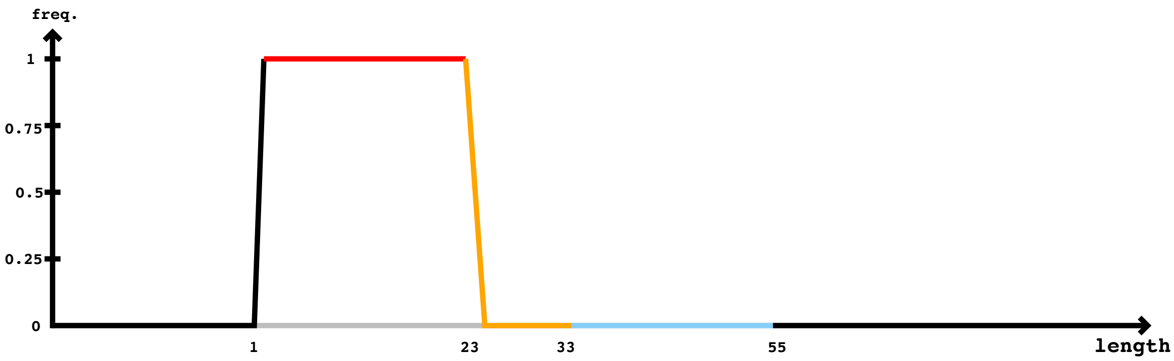

Mature Star

```
5'- aauccagguccaccaggaguugcugguaacccuggaaagccuggaaaaccaggaaagccaggagcaccaggaaacccgggugcaccaggaaaaggagcagcuguuccaugcg  -3'  exp
    ..(((.(((.((((.((..((.(((((...(((((....(((((....)))))....)))))...))))).))..)).)))).))).)))...(((((....)))))..... reads  mm  sample
    ...................ugcugguaacccuggaaagccu........................................................................ 71     0   seq
```

Supplementary Figure 14. Stem loop structure, read counts and miRNA annotation as determined by miRDeep2 software of novel miRNA candidates.

Provisional ID	:	X_42835
Score total	:	1.5
Score for star read(s)	:	-1.3
Score for read counts	:	0
Score for mfe	:	1.2
Score for randfold	:	1.6
Score for cons. seed	:	
Total read count	:	15
Mature read count	:	15
Loop read count	:	0
Star read count	:	0

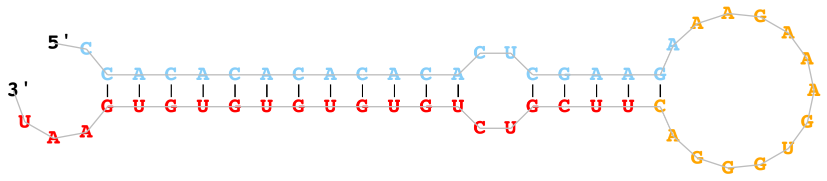

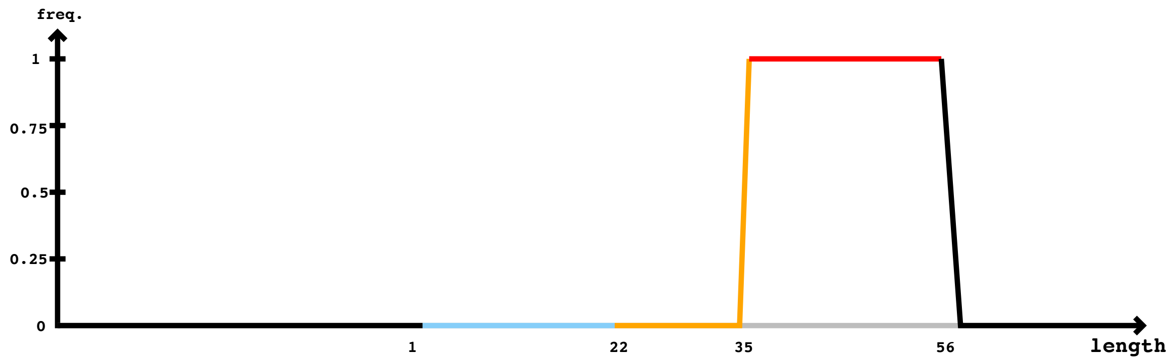

Star Mature

```
5'- gagaagagagcaacaugauuuuuuaaaauuguuacaccacacacacacacucgaagaaagaaaguggacuucgucugugugugugugaauguugcgaaauacacuuuuuc   -3'   exp
    .........((((((((((((....))))).......((((((((((((..(((((.............)))))..)))))))))))).)))))))..............   reads   mm   sample
    .....................................................................uucgucugugugugugugaau....................   15      0    seq
```

Supplementary Figure 14. Stem loop structure, read counts and miRNA annotation as determined by miRDeep2 software of novel miRNA candidates.

Provisional ID	:	X_42247
Score total	:	1.2
Score for star read(s)	:	-1.3
Score for read counts	:	0
Score for mfe	:	0.9
Score for randfold	:	1.6
Score for cons. seed	:	
Total read count	:	119
Mature read count	:	119
Loop read count	:	0
Star read count	:	0

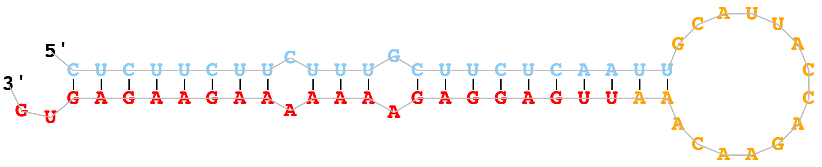

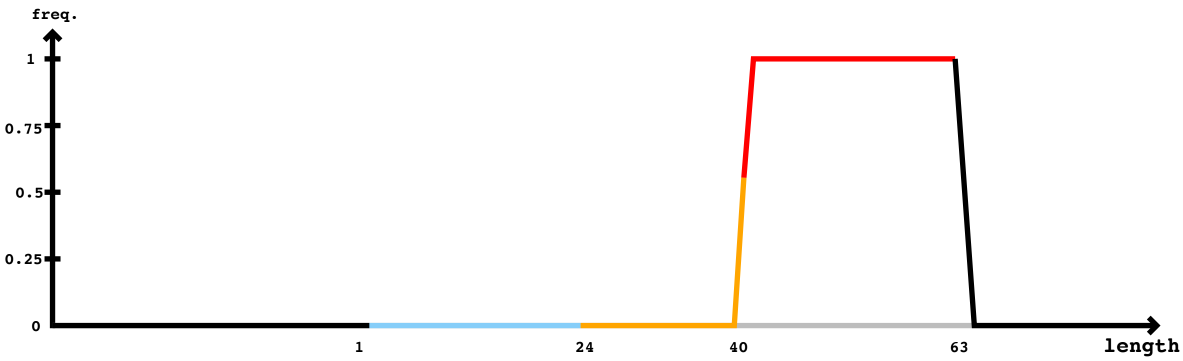

Star Mature

```
5'- gagguggucucgcugacacuuuuucucugaccucuucuucuuugcuucucaauugcauuaccagaacaaauugaggagaaaaaaagaagagugcggaaaaggaaaaaaaaaca  -3'   exp
    ((((((.((.....))))))))...((((..((((((((.(((.((((((((((..............))))))))))).))).))))))))..))))................    reads    mm    sample
    ......................................................................uugaggagaaaaaaagaagagug....................    66       0     seq
    .......................................................................ugaggagaaaaaaagaagagugc...................    53       0     seq
```

Supplementary Figure 14. Stem loop structure, read counts and miRNA annotation as determined by miRDeep2 software of novel miRNA candidates.

Provisional ID	:	X_44338
Score total	:	2.1
Score for star read(s)	:	-1.3
Score for read counts	:	0
Score for mfe	:	1.8
Score for randfold	:	1.6
Score for cons. seed	:	
Total read count	:	108
Mature read count	:	108
Loop read count	:	0
Star read count	:	0

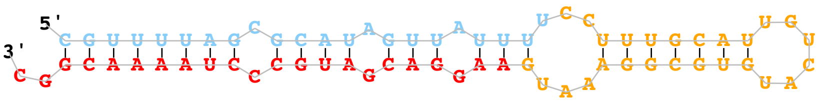

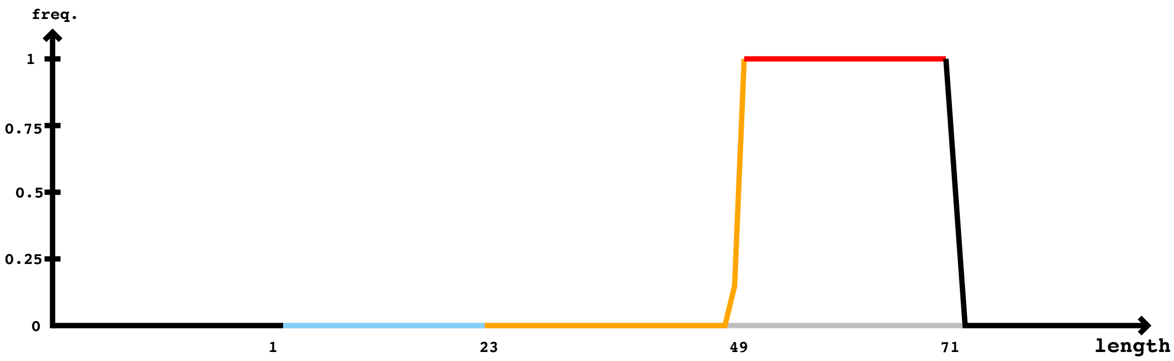

Star Mature

```
5'- guucaguuggcauguuuaagucguuuuagcgcauaguuauuuuccuuugcauugucaugugcggaaaugaaggacgaugcccuaaaacggcuggaggugucgggaggcugu   -3'    exp
    (((((...((((((.((((.((((((((((.((((.(((.(((...(((((((......)))))))...))).))).)))).)))))))))))))).))))))).)))))...     reads      mm        sample
    ...................................................................gaaggacgaugcccuaaaacggc.....................     16         0         seq
    ....................................................................aaggacgaugcccuaaaacggU.....................     15         1         seq
    ....................................................................aaggacgaugcccuaaaacggc.....................     53         0         seq
    ....................................................................aaggacgaugcccuaaaacggcu....................     24         0         seq
```

Supplementary Figure 14. Stem loop structure, read counts and miRNA annotation as determined by miRDeep2 software of novel miRNA candidates.

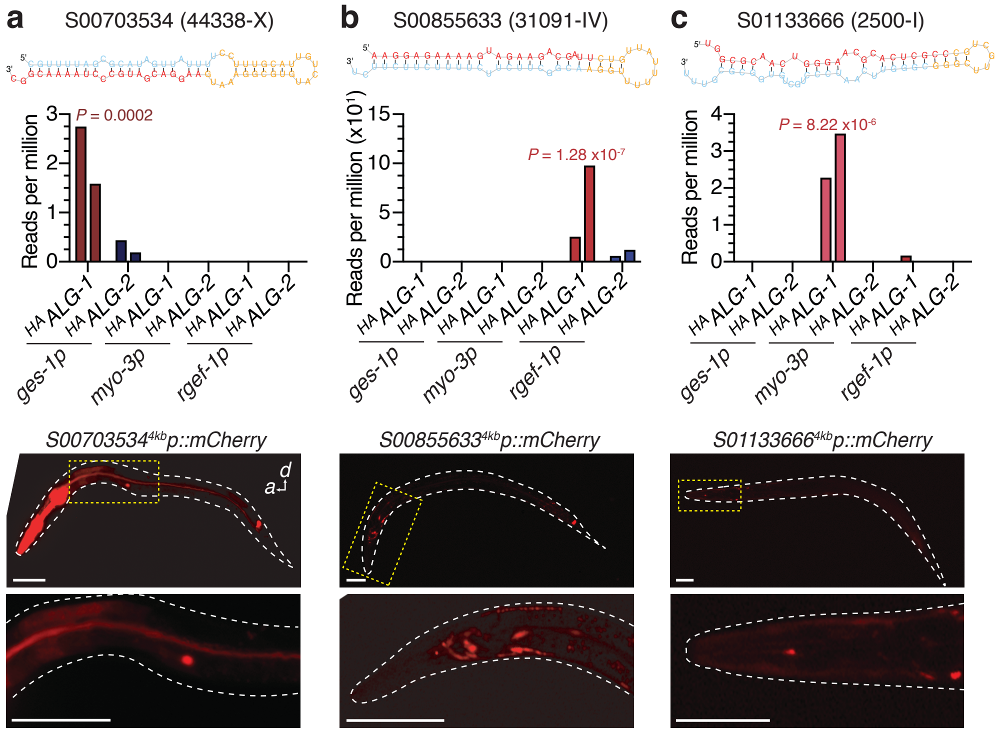

Supplementary Figure 15. Examples of novel miRNA candidate cell-type specific expression and loading. (a-c) Predicted secondary structures of pre-miRNAs, read counts per tissue- and AGO-type loading, and representative images of promoter mCherry fusion strains for (**a**) intestine-specific (**b**) neuronal-specific, and (**c**) body wall muscle-specific novel miRNAs. For (**a**) to (**c**), graph shows reads per million in indicated cell types of two biological replicate experiments, two bars. Scale bars, 50 µm.

Supplementary Table 1: List of the generated transgenic strains used in this study

Strain Name	**Genotype**
SJZ284	*foxSi62[ges-1p::HA::ALG-1::SL2::HIS-58::GFP::tbb-2 3'UTR (oxti177)IV]*
SJZ285	*foxSi63[ges-1p::HA::ALG-2::SL2::HIS-58::GFP::tbb-2 3'UTR (oxti177)IV]*
SJZ286	*foxSi64[rgef-1p::HA::ALG-1::SL2::HIS-58::GFP::tbb-2 3'UTR (oxti177)IV]*
SJZ287	*foxSi65[rgef-1p::HA::ALG-2::SL2::HIS-58::GFP::tbb-2 3'UTR (oxti177)IV]*
SJZ290	*foxSi68[myo-3p::HA::ALG-1::SL2::HIS-58::GFP::tbb-2 3'UTR (oxti177)IV]*
SJZ291	*foxSi69[myo-3p::HA::ALG-2::SL2::HIS-58::GFP::tbb-2 3'UTR (oxti177)IV]*
SJZ1001	*mir-60(n4947)II; foxSi166[elt-2p::mir-60::SL2::HIS-58::GFP::tbb-2 3'UTR (oxti177)IV]*
SJZ1002	*mir-60(n4947)II; foxSi167[rgef-1p::mir-60::SL2::HIS-58::GFP::tbb-2 3'UTR (oxti177)IV]*
SJZ1003	*mir-60(n4947)II; foxSi168[myo-3p::mir-60::SL2::HIS-58::GFP::tbb-2 3'UTR (oxti177)IV]*
SJZ845	*fox30[3xFLAG::GFP::alg-1]*
SJZ846	*alg-2(ok304)II; fox30[3xFLAG::GFP::alg-1]X*
SJZ844	*ap43[3xFLAG::mKate2::alg-2]II;alg-1(gk214)]X*
SJZ1048	*foxEx276[mir-77p::mir-77::SL2:HIS-58::GFP::tbb-2 3'UTR]*
SJZ1051	*foxEx279[mir-238p::mir-238::SL2:HIS-58::GFP::tbb-2 3'UTR]*
SJZ1036	*alg-1(gk314)X; foxEx253[eft-3p::HA::ALG-2::SL2::HIS-58::GFP::tbb-2 3;UTR]*
SJZ1037	*alg-1(gk314)X; foxEx255[ges-1p::HA::ALG-1::SL2::HIS-58::GFP::tbb-2 3'UTR]*
SJZ1038	*foxSi63[ges-1p::HA::ALG-2::SL2::HIS-58::GFP::tbb-2 3'UTR (oxti177)IV]; alg-1(gk314)X*
SJZ998	*foxSi134[rgef-1p::FLAG::RPL-18::SL2::HIS-58::GFP::tbb-2 3'UTR (oxti177)IV]*
SJZ1000	*alg-2(ok304)II;foxSi134[rgef-1p::HA::RPL-18::SL2::HIS-58::GFP::tbb-2 3'UTR (oxti177)IV]*
SJZ832	*foxEx230(pS00703534(4kb)::mCherry)*
SJZ783	*foxEx212(pS00855633(4kb)::mCherry)*
SJZ786	*foxEx215(pS01133666(4kb)::mCherry)*

Supplementary Table 2: List of primers used for this study

	Name	Sequence (5' to 3')
Genotyping	**sz6-oCFJ1529**	TATCGTAAATCGGCGCGAGC
	**sz13-oCFJ1491**	gtcactcaaaccgatgcaga
	**sz14-oCFJ1492**	gcaatttcggcaatttcagt
	**miR-60-F**	ATGATGCATGCAACTAAGTC
	**miR-60-F1**	CACAATTCTCTTTTATAGTG
	**miR-60-R**	AGCCTTTGTCAATCCGTTGC
	**RB574-F**	GACTTAGCCTAGGCAAGCCC
	**RB574-F1**	CGATGCCAGTCATCGAATTCG
	**RB574-R**	GCTTACTAGCTTACAAATCTC
	**VC446-F**	CCAAAAATGCATGACGATC
	**VC446-F1**	TGATCTAGAAGTAAGTTTTCATCG
	**VC446-R**	GAACATCGCTAAATGTTTGTGG
	**miR-60-F**	ATGATGCATGCAACTAAGTC
	**miR-60-F1**	CACAATTCTCTTTTATAGTG
	**miR-60-R**	AGCCTTTGTCAATCCGTTGC
Probes	**miR-2**	GCACATCAAAGCTGGCTGTGATA
	**Lin-4**	TCACACTTGAGGTCTCAGGGA
	**U6**	ATCTTCTCTGTATTGTTCC
Cloning	**EFT3p-F**	ATGGATCCGCACCTTTGGTCTTTTATTG
	**EFT3p-R**	ATCTCGAGTGAGCAAAGTGTTTCCCAACTG
	**myo-3p-F**	ATGGATCCCGGCTATAATAAGTTCTTGAA
	**myo-3p-R**	ATCTCGAGTCTAGATGGATCTAGTGGTC
	**rgef-1p-F**	ATGGATCCCGTTTCCGATACCCCCTTATA
	**rgef-1p-R**	CAGATATCCTTTACTGCTGATCGTCGTCG
	**ges-1p-F**	ATGGATCCCGGCTATAATAAGTTCTTGAA
	**ges-1p-R**	ATCTCGAGCTGAATTCAAAGATAAGATATG
	**unc-25p-F**	CAGGATCCGCATGCAAAAAACACCCACTT
	**unc-25p-R**	CACTCGAGTTTTGGCGGTGAACTGAGCTT
	**Fl-RPL18-F**	ATGGATTATAAAGACGATGACGATAAGATGGGTATTGATATCAACCAC
	**RPL18-SL2-R**	AAAGTAGGATGAGACAGCTTAGTTCTTGTAGGCTCTGG
	**Fl-F**	ATGGATTATAAAGACGATGACGATAAG
	**HA-ALG1-F**	ATGTATCCGTATGATGTTCCTGATTATGCTggaggatccggaggaATGTCCGGCGGGCCGCAATATTTG
	**ALG1-2-R**	GGGGACCACTTTGTACAAGAAAGCTGGGTcTTAAGCAAAGTACATGACGTTG
	**HA-ALG2-F**	ATGTATCCGTATGATGTTCCTGATTATGCTggaggatccggaggaATGTTCCCTCTGCCTGTACAC
	**ALG2-2-R**	GGGGACCACTTTGTACAAGAAAGCTGGGTcTTAGGCAAAATACATGACGTTG
	**allgene-HA-1-F**	GGGGACAAGTTTGTACAAAAAAGCAGGCTtaAGAAAAAATGTATCCGTATGATGTTCC
	**SL2-2-F**	GGGGCAGCTTTCTTGTACAAAGTGGGAATGCCACCAAAGCCATCTG
	**tbb2-3-R**	GGGGACAACTTTGTATAATAAAGTTGctaTGAGACTTTTTTCTTGGCGG
	**F-RPL18-F**	ATGGATTATAAAGACGATGACGATAAGATGGGTATTGATATCAACCAC
	**RPL18-SL2-R**	AAAGTAGGATGAGACAGCTTAGTTCTTGTAGGCTCTGG
	**ALG1-1**	ACGTTGTAAAACGACGGCCAGTCGCCGGCATTGATCGTGTTCATTCCGAA
	**ALG1-2**	TCCAGTGAACAATTCTTCTCCTTTACTCATGAGAGGGATTGAAAGCGCTGGGTTGG
	**ALG1-3**	CGTGATTACAAGGATGACGATGACAAGAGAATGGCTGCCGAACTGAACAAC
	**ALG1-4**	TCACACAGGAAACAGCTATGACCATGTTATCAACGTTCTCACAGACAG
	**Cas9-sg-ALG1-F**	AGCGCTTTCAATCCCTCTCAGTTTTAGAGCTAGAAATAGCAAGT
	**Cas9-sg-ALL-R**	CAAGACATCTCGCAATAGG
	**pri-miR60-F**	CTCGAAAACCGCTTGTTCTTG
	**pri-miR60-SL2-R**	GTGAAAGTAGGATGAGACAGCCGATTTCTCAAGTCTTGAAC
	**CD-3**	TTTTTCTGCTAGCTTAATTAAGCCGGCGGCCGCCTCAACTTTTCTATACAAAGTTGATAGC
	**elt2p-Not-I-F**	ATGCGGCCGCGGATCCCCCGGGCTGCAGGA
	**elt2p-NaeI-R**	ATGCCGGCTCTATAATCTATTTTCTAGT
	**miR-67p-NotI-F**	AAGCGGCCGCGGTGAGCTTCGATACTTTTTC
	**miR-67p-PacI-R**	ACTTAATTAAAGTTGGAATACCAGAGAATT
	**miR-77p-NotI-F**	ATCGCGGCCGCACTTCGAGTTTTTGAAATCCG
	**miR-77-NheI-R**	CTAGCTAGCCAAACTCTATACCAATTTGG
	**miR-238p-NotI-F**	ATCGCGGCCGCAAAAGAACGTTGAGTTAC
	**mCh-F**	ATGGTCTCAAAGGGTGAAGAAG
	**unc54-R**	AAACAGTTATGTTTGGTATATTGG
	**miR-238-NheI-R**	CTAGCTAGCCATGGCTCATAACTATCTG
	**AP4-S00855633-R**	ATCTTCTTCACCCTTTGAGACCATCGTGGAAAATCATACTTGTCCAACC
	**AP5-S00855633-F**	TTGAATGCGAGAAGAGAGATACGGA
	**AP6-S00855633-F***	CTTAAAAGGGGGAACCGCTCG
	**AP10-S01133666-R**	ATCTTCTTCACCCTTTGAGACCATGACAGGAGGTTGCTTATGAATGC
	**AP11-S01133666-F**	TGGAATGTGCCTGTAATTCTTGAG
	**AP12-S01133666-F***	TTCATGTGCGTCGTGTTGTTTCG
	**AP15-S00703534-R**	ATCTTCTTCACCCTTTGAGACCATGCTTCTTGGGGGGAAAGTCTAATAG
	**AP16-S00703534-F**	GTGTCGAAGGAAAAGTTGCTTGAG
	**AP17-S00703534-F***	CTTTCATGTTCTGTTTCGCGCAG
q-RT-PCR	**IK-44**	CAGGTCCAGTTTTTTTTTTTTTTTVN
	**miR-1-3p-F**	CGCAGTGGAATGTAAAGAAGT
	**miR-1-3p-R**	CAGGTCCAGTTTTTTTTTTTTTTTACAT
	**miR-67-3p-F**	CGCAGTCACAACCTCCTAGAAAG
	**miR-67-3p-R**	CAGGTCCAGTTTTTTTTTTTTTTTCTACT
	**miR-71-5p-F**	CGCAGCGGTACGATCGCGGCG
	**miR-71-5p-R**	CAGGTCCAGTTTTTTTTTTTTTTTGATATCC
	**miR-90-3p-F**	CGCAGTGATATGTTGTTTGAATG
	**miR-90-3p-R**	CAGGTCCAGTTTTTTTTTTTTTTTAGGGG
	**miR-791-3p-F**	CGCAGTTTGGCACTCCGCAGATAGGCAA
	**miR-791-3p-R**	CAGGTCCAGTTTTTTTTTTTTTTTGCCT
	**Lsy-6-3p-F**	CGCAGTTTTGTATGAGACGCATTTCGA
	**Lsy-6-3p-R**	CAGGTCCAGTTTTTTTTTTTTTTTCGAAA
	**miR-60-3p-F**	CGCAGTATTATGCACATTTTCT
	**miR-60-3p-R**	CAGGTCCAGTTTTTTTTTTTTTTTGAACT
	**miR-83-3p-F**	CGCAGTAGCACCATATAAATT
	**miR-83-3p-R**	CAGGTCCAGTTTTTTTTTTTTTTTACTG
	**miR-35-3p-F**	CAGTCACCGGGTGGAAACTA
	**miR-35-3p-R**	CAGGTCCAGTTTTTTTTTTTTTTTACTGC
	**miR-234-3p-F**	CGCAGTTATTGCTCGAGAATA
	**miR-234-3p-R**	CAGGTCCAGTTTTTTTTTTTTTTTAAGGG

Cell-type-specific profiling of loaded miRNAs from *Caenorhabditis elegans* reveals spatial and temporal flexibility in Argonaute loading

Christopher A Brosnan[1,2,*], Alexander Palmer[1] and Steven Zuryn[1,*].

Supplementary Note 1:

Identification of novel miRNA candidates in *C. elegans*

The sensitivity of detection combined with the cell-type-specific resolution of our approach enabled us not only to identify a large set of isomiRs that were loaded into silencing complexes, but also to discover 37 new candidate miRNA loci in this set (~15% of the total number previously identified in *C. elegans,* Supplementary Fig. 13 and 14) that satisfied stringent criteria (including nucleotide length) and mapped across all chromosomes in the *C. elegans* genome (Supplementary Data 3, Supplementary Data 5 and Supplementary Fig. 13). Importantly, aside from loading into AGO proteins, each of the mature miRNA sequences mapped to loci predicted to form low energy hairpin secondary structures typical of pri-miRNAs (Supplementary Fig. 13 and 14). Interestingly, the novel candidate miRNAs also demonstrated a high degree of cell- and AGO-type specificity, suggesting that they have specialized activities, which in addition to their relatively low read count levels may have hindered their discovery until now (Supplementary Fig. 13 and 14, Supplementary Data 3 and 5). To further validate their authenticity and determine their expression patterns, we generated transgenic strains carrying transcriptional fluorescent reporters (*mir-promoter^{4kb}::mCherry*) of three examples of novel miRNA candidates that were loaded into AGOs in either the intestine, BWM, or nervous system. Each of the miRNA reporters exhibited an expression pattern that was consistent with its spatially unique AGO interactions (Supplementary Fig. 15; intestine, neuron and BWM respectively). This confirmed that the putative *cis*-regulatory regions selected to drive the reporter were transcriptionally functional, indicatory of an expressible miRNA locus that matched their spatial loading patterns.
