# Supplementary material for: Cell-type-specific profiling of loaded miRNAs from Caenorhabditis elegans reveals spatial and temporal flexibility in Argonaute loading
Source: Nat Commun. 2021 Apr 13;12:2194. doi: 10.1038/s41467-021-22503-7 (PMC8044110; doi:10.1038/s41467-021-22503-7)
Supplement: Supplementary file 8 — Description of Additional Supplementary Files [file 41467_2021_22503_MOESM8_ESM.docx]

**Description of additional supplementary information:**

**Title: Supplementary Data 1.**

Description: List of miRNAs that were significantly associated (log_2_ fold change>2, *P*<0.05) with in one or more of either ALG-1 or ALG-2 in the intestine, BWM, or nervous system.

**Title: Supplementary Data 2.**

Description: Comparison between miRNAs loaded in ALG-1 and ALG-2 in the indicated tissue types found in this study with those found in Alberti et al.

**Title: Supplementary Data 3.**

Description: List of miRNAs (isomers) with distinguishable sequences from reference miRNA sequences.

**Title: Supplementary Data 4.**

Description: List of miR-71 miRNA sequence variants differentially loaded into ALG-1 and ALG-2 in the indicated tissue types.

**Title: Supplementary Data 5**.

Description: List of potential novel miRNA candidates identified in the present study.

**Title: Supplementary Data 6.**

Description: List of mRNAs significantly up- or downregulated in neuron specific polysome libraries of either WT or *alg-2*.
