# Supplementary material for: Cell-type-specific profiling of loaded miRNAs from Caenorhabditis elegans reveals spatial and temporal flexibility in Argonaute loading
Source: Nat Commun. 2021 Apr 13;12:2194. doi: 10.1038/s41467-021-22503-7 (PMC8044110; doi:10.1038/s41467-021-22503-7)
Supplement: Supplementary file 9 — Reporting Summary [file 41467_2021_22503_MOESM9_ESM.pdf]

nature research

Corresponding author(s): Steven Zuryn
Christopher Brosnan

Last updated by author(s): Mar 9, 2021

Reporting Summary

Nature Research wishes to improve the reproducibility of the work that we publish. This form provides structure for consistency and transparency in reporting. For further information on Nature Research policies, see our Editorial Policies and the Editorial Policy Checklist.

Statistics

For all statistical analyses, confirm that the following items are present in the figure legend, table legend, main text, or Methods section.

n/a	Confirmed	
☐	☒	The exact sample size (*n*) for each experimental group/condition, given as a discrete number and unit of measurement
☐	☒	A statement on whether measurements were taken from distinct samples or whether the same sample was measured repeatedly
☐	☒	The statistical test(s) used AND whether they are one- or two-sided<br>*Only common tests should be described solely by name; describe more complex techniques in the Methods section.*
☐	☒	A description of all covariates tested
☐	☒	A description of any assumptions or corrections, such as tests of normality and adjustment for multiple comparisons
☐	☒	A full description of the statistical parameters including central tendency (e.g. means) or other basic estimates (e.g. regression coefficient) AND variation (e.g. standard deviation) or associated estimates of uncertainty (e.g. confidence intervals)
☐	☒	For null hypothesis testing, the test statistic (e.g. *F*, *t*, *r*) with confidence intervals, effect sizes, degrees of freedom and *P* value noted<br>*Give P values as exact values whenever suitable.*
☒	☐	For Bayesian analysis, information on the choice of priors and Markov chain Monte Carlo settings
☒	☐	For hierarchical and complex designs, identification of the appropriate level for tests and full reporting of outcomes
☒	☐	Estimates of effect sizes (e.g. Cohen's *d*, Pearson's *r*), indicating how they were calculated

Our web collection on statistics for biologists contains articles on many of the points above.

Software and code

Policy information about availability of computer code

Data collection

qRT PCR data was collected using: Light Cycler 480W v1.5
Phosphoimager scans were acquired using:Typhoon FLA9500 control software v1.1
Chemiluminescence western blot images were collected using: Bio-Rad Image Lab software v2.3 or Image Studio Lite software v5.2.5
Image processing was performed in Fiji - ImageJ v2.0.0
Prism - GraphPad Software inc. v8.4.0 (145)
Wormlab (v2019.1.1)

Data analysis

Raw small-RNA-seq data were processed using cutadapt (v2.10) 75. Small-RNA reads were subsequently mapped to the C. elegans reference genome (WBcel235) with bowtie2 (v2.3.4.3) and annotated to known mature miRNAs (miRbase, v21) using FeatureCounts (Subread v2.0.0) Pairwise comparisons of miRNAs between argonautes and tissues were performed with edgeR (v3.11).
Novel candidate miRNAs were annotated using miRDeep2 (v2.0.0.8).

Raw mRNA-seq data were trimmed using cutadapt v2.10. Reads were further mapped to the C. elegans genome (WBcel235) with HISAT2 v2.10 and annotated to known mRNAs (ensembl, release 96) using FeatureCounts (Subread v2.0.0). Differential expression between samples was performed using edgeR (v3.11). Gene ontology of significant mRNAs was annotated using biomaRt (2.46.0).

All script is available at https://github.com/Alexander-Palmer/smallRNA-polymRNA

For manuscripts utilizing custom algorithms or software that are central to the research but not yet described in published literature, software must be made available to editors and reviewers. We strongly encourage code deposition in a community repository (e.g. GitHub). See the Nature Research guidelines for submitting code & software for further information.

Data

Policy information about availability of data

All manuscripts must include a data availability statement. This statement should provide the following information, where applicable:

- Accession codes, unique identifiers, or web links for publicly available datasets
- A list of figures that have associated raw data
- A description of any restrictions on data availability

All sequencing data have been uploaded to GEO (GSE156716).
Raw data have been compiled for Figure 1, Figure 3, Figure Figure S3 and are presented as a source data file.

Field-specific reporting

Please select the one below that is the best fit for your research. If you are not sure, read the appropriate sections before making your selection.

☒ Life sciences ☐ Behavioural & social sciences ☐ Ecological, evolutionary & environmental sciences

For a reference copy of the document with all sections, see nature.com/documents/nr-reporting-summary-flat.pdf

Life sciences study design

All studies must disclose on these points even when the disclosure is negative.

Sample size	No Statistical method was used to predetermine the sample size used. Large populations of animals (~5,000 to 20,000 or ~100,000) were used throughout. Behavioural assays were performed on smaller numbers randomly selected from larger populations. These numbers are in line with those previously used in the field and in the host lab to measure body area and fat staining measurements. For sequencing experiments either biological duplicates (ALG:miRNA immunoprecipitations) or biological triplicates (RPL18 polysome immunoprecipitations) were performed with each replicate containing at least 100000 animals. The number of replicated and pool size were determined base on previously conducted large scale sequencing experiements.
Data exclusions	No data was excluded except for sequencing experiments where miRNAs and mRNA with low expression (< 1 reads per million) were excluded.
Replication	All replications of experiments were successful including sequencing, small RNA detection, immunoprecipitation, qPCR, imaging and behavior. With the exception of deep sequencing all experiments were repeated independently three times with similar results seen.
Randomization	Animals were selected randomly from larger groups for every experiment. Animal groups were determined by there genetic background (e.g Wild-type, mutant, transgenic and combinations thereof), with each group considered to be isogenic. Large pools of animals were typically used and random animals selected from within these with no bias were grouped experimentally.
Blinding	The investigators were not blinded to allocation during any data collection or analysis. The genotypes used were well known to the investigator and often were obvious to differentiate based on visual phenotypes making blinding impractical.

Reporting for specific materials, systems and methods

We require information from authors about some types of materials, experimental systems and methods used in many studies. Here, indicate whether each material, system or method listed is relevant to your study. If you are not sure if a list item applies to your research, read the appropriate section before selecting a response.

Materials & experimental systems

n/a	Involved in the study	
☐	☒	Antibodies
☒	☐	Eukaryotic cell lines
☒	☐	Palaeontology and archaeology
☐	☒	Animals and other organisms
☒	☐	Human research participants
☒	☐	Clinical data
☒	☐	Dual use research of concern

Methods

n/a	Involved in the study	
☒	☐	ChIP-seq
☒	☐	Flow cytometry
☒	☐	MRI-based neuroimaging

Antibodies

Antibodies used	Anti-HA Magnetic Beads (88837 - Pierce)<br>Anti-DYKDDDDK Magnetic Beads (A36798 - Pierce)<br>Anti-HA (C29F4) rabbit (ref 3724 - Cell Signaling)

	Anti-FLAG M2 (F3165 - Sigma-Aldrich)<br>Anti-tubulin (T6074 - Sigma)<br>Goat-anti-rabbit-HRP (ab6721 - Abcam)<br>Goat-anti-rat-HRP (7077S - Cell Signaling)<br>IRDye 680RD goat-anti-mouse (Li-COR - 926-68070)<br>IRDye 800CW goat-anti-rabbit (Li-COR - 926-32211)
Validation	Anti-HA (C29F4): https://antibodyregistry.org/search.php?q=AB_1549585<br><br>Anti-FLAG M2 (F3165): https://antibodyregistry.org/search.php?q=AB_259529<br><br>Anti-tubulin (T6074): https://antibodyregistry.org/search.php?q=AB_477582<br><br>Goat-anti-rabbit-HRP (ab6721 - Abcam): https://antibodyregistry.org/search.php?q=AB_955447<br><br>Goat-anti-rat-HRP (7077S - Cell Signaling): https://antibodyregistry.org/search?q=7077S.<br><br>IRDye 680RD goat-anti-mouse (Li-COR - 926-68070): https://antibodyregistry.org/search.php?q=AB_10956588.<br><br>IRDye 800CW goat-anti-mouse (Li-COR - 926-32211): https://antibodyregistry.org/search.php?q=AB_621843

Animals and other organisms

Policy information about studies involving animals; ARRIVE guidelines recommended for reporting animal research

Laboratory animals	Caenorhabditis elegans
Wild animals	This study did not involve wild animals.
Field-collected samples	This study did not involve field-collected samples.
Ethics oversight	No ethical approval was required.

Note that full information on the approval of the study protocol must also be provided in the manuscript.
